# Gene expression profiling in a mouse model of infantile neuronal ceroid lipofuscinosis reveals upregulation of immediate early genes and mediators of the inflammatory response

**DOI:** 10.1186/1471-2202-8-95

**Published:** 2007-11-16

**Authors:** Xingwen Qiao, Jui-Yun Lu, Sandra L Hofmann

**Affiliations:** 1 Hamon Center for Therapeutic Oncology Research and the Department of Internal Medicine, University of Texas Southwestern Medical Center, Dallas, TX, 75390, USA

## Abstract

**Background:**

The infantile form of neuronal ceroid lipofuscinosis (also known as infantile Batten disease) is caused by hereditary deficiency of a lysosomal enzyme, palmitoyl-protein thioesterase-1 (PPT1), and is characterized by severe cortical degeneration with blindness and cognitive and motor dysfunction. The PPT1-deficient knockout mouse recapitulates the key features of the disorder, including seizures and death by 7–9 months of age. In the current study, we compared gene expression profiles of whole brain from PPT1 knockout and normal mice at 3, 5 and 8 months of age to identify temporal changes in molecular pathways implicated in disease pathogenesis.

**Results:**

A total of 267 genes were significantly (approximately 2-fold) up- or downregulated over the course of the disease. Immediate early genes (Arc, Cyr61, c-fos, jun-b, btg2, NR4A1) were among the first genes upregulated during the presymptomatic period whereas immune response genes dominated at later time points. Chemokine ligands and protease inhibitors were among the most transcriptionally responsive genes. Neuronal survival factors (IGF-1 and CNTF) and a negative regulator of neuronal apoptosis (DAP kinase-1) were upregulated late in the course of the disease. Few genes were downregulated; these included the α2 subunit of the GABA-A receptor, a component of cortical and hippocampal neurons, and Hes5, a transcription factor important in neuronal differentiation.

**Conclusion:**

A molecular description of gene expression changes occurring in the brain throughout the course of neuronal ceroid lipofuscinosis suggests distinct phases of disease progression, provides clues to potential markers of disease activity, and points to new targets for therapy.

## Background

An unusual group of lysosomal storage disorders, the neuronal ceroid lipofuscinoses, are characterized by retinal and cortical neurodegeneration with scant autofluorescent storage material that accumulates in the brain and peripheral tissues (reviewed in [1]). Distinct subsets of NCL are recognized based on characteristic appearance by electron microscopy that include granular osmiophilic deposits and/or various membrane profiles (curvilinear, fingerprint, and rectilinear) [2]. Autosomal recessive mutations in at least seven different genes are responsible for these disorders [3], and while the function of only a few is known, each appears to participate in some aspect of endo/lysosomal function [4]. The CLN1 (ceroid lipofuscinosis, neuronal-1) gene encodes a soluble lysosomal palmitoyl-protein thioesterase (PPT1) that functions to remove fatty acids (usually palmitate) from modified cysteine residues in proteins [5,6]. A growing number of studies implicate PPT1 in the maintenance of synaptic vesicle number [7] and function [8-10].

The PPT1 knockout mouse is an excellent model for infantile neuronal ceroid lipofuscinosis, recapitulating the major findings in the disease [11-15]. An orderly series of pathological events have been described in the central nervous system of PPT1 deficient mice [16] that includes localized astrocytosis, first detected between one and three months of age, with earliest changes detected in the thalamic relay nuclei of the visual system (dorsal lateral geniculate nucleus). The progression of neuronal loss proceeds from thalamic relay neurons to interneurons and finally, to corresponding target cortical granule neurons. The observed marked effect on thalamic nuclei in the mice is consistent with early thalamic hypointensity in MRI studies of INCL patients [17]. Loss of inhibitory interneurons corresponds temporally with onset of seizures at 7 months. Microglial (macrophage) activation is a prominent feature, first detectable during later stages of disease, between 3 and 5 months. Death occurs in most animals by 9 months of age [11,12].

Despite a great deal of understanding at the cellular level, useful information may be gained from a more detailed description of the progress of the disorder at the level of gene expression. In this study we have followed the expression of approximately 34,000 genes in the brains of normal and PPT1 deficient mice at three points during the development of the neurological disorder, and reveal a transcriptional landscape that will be of value in understanding this and other neurodegenerative conditions.

## Results

### Study design and microarray comparisons

RNA was extracted from whole brains of wild-type and PPT1 knockout mice at 3, 5, and 8 months of age (three animals in each group, total of 9 wild-type and 9 knockout animals) and hybridized to Affymetrix mouse expression array 430 2.0 chips to obtain gene expression profiles of 45,101 probe sets corresponding to 34,000 well-characterized mouse genes.

**Figure 1:**
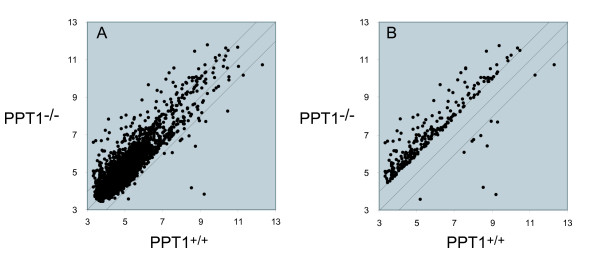
**Scatter plot of gene expression profiles of PPT1 knockout vs. wild-type mouse brain**. Each point represents a unique probe set. Y- and X-axis values are expressed as the logarithm of expression intensity for each probe set. (A) All probe sets (a total of 5236) that passed filtering as described under "Materials and Methods" are shown. (B) Probe sets (total of 267) yielding a significant difference (as determined by SAM analysis under "Methods" between the average of the knockout and wild-type classes are shown.

**Figure 2:**
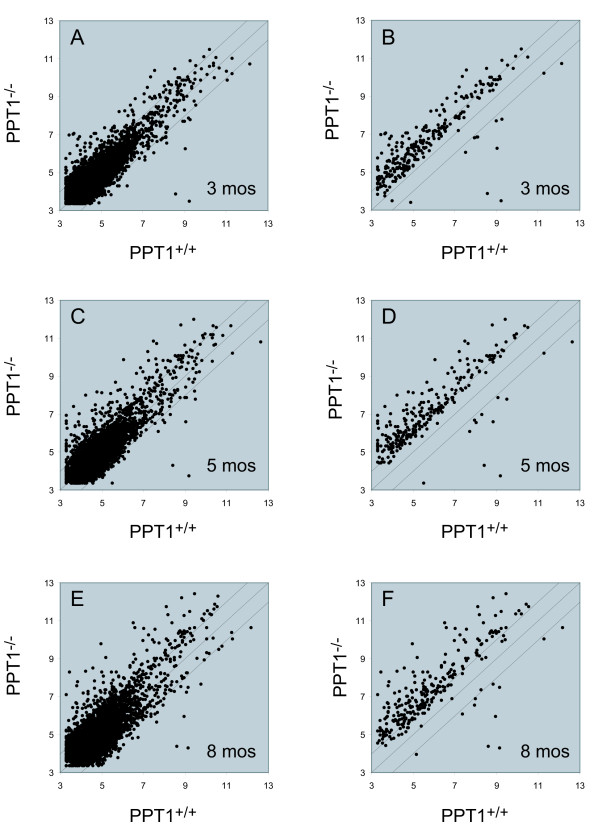
**Scatter plot of gene expression profiles at 3, 5 and 8 months of age**. Each point represents a unique probe set. Y- and X-axis values are expressed as the logarithm of expression intensity for each probe set. (A, C, E) Groupwise comparisons between wild-type and PPT1 knockout at 3, 5, and 8 months for all probe sets (5236) that passed filtering as described under "Materials and Methods". (B, D, F) Groupwise comparison between wild-type and PPT1 knockout mice showing only those probe sets selected by SAM (significance) analysis (total of 267). The scatter plot indicates an increasing number of gene expression changes with time in the knockout mice.

A gene filter was applied that excluded two classes of genes: 1) those with detection call 'Absent' that exceeded 80% of the expression data values and 2) those in which less than 20% of the expression data values had at least a 1.5-fold change in either direction from the median value of that gene. This filter will select for genes that show relatively large fold-changes (on the order of 1.5-fold or more) in knockout vs. wild-type. A total of 5236 probe sets passed filtering, and the microarray data were analyzed using the algorithm Significance Analysis of Microarrays (SAM) [18] (false discovery rate, 0.05, 1000 permutations, confidence level 90%) (see Methods). A total of 267 probe sets were identified as significantly differentially expressed between the two phenotypes, taken as a whole (Fig. 1). These probe sets represent 235 unique named genes (Additional File 1). Among these genes highly regulated genes, 227 genes were upregulated, and 8 genes were downregulated. A comparison of gene expression changes between PPT1 knockout and wild-type showed increased scatter with increasing time at 3, 5, and 8 months, as increasingly more genes were observed to be over- or underexpressed in PPT1 vs. wild-type mouse brains (Fig. 2).

**Figure 3:**
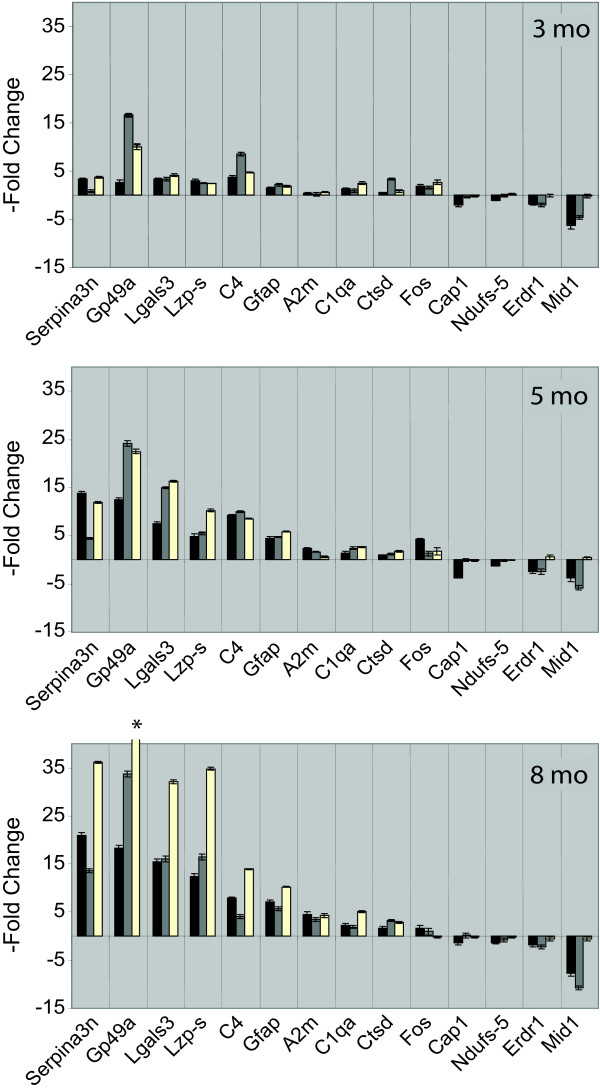
**Validation of a subset of regulated genes by quantitative PCR**. Values represent the mean ± standard deviation of the -fold change of PPT1 knockout as compared to wild-type expression (n = 3 for each value). Black bars, values obtained from microarray hybridization. Gray bars, values obtained from quantitative PCR analysis of the original 18 samples used for the microarray hybridizations. White bars, values obtained from an independent set of 18 samples analyzed by quantitative PCR. *, off-scale value is 103 ± 0.33 -fold.

A validation experiment was run using a sampling of 14 genes from the list of 267 differentially-expressed genes by quantitative real-time PCR using the fluorescent dye SYBR green I (Fig. 3). Two sets of samples were assayed-samples from the original RNA samples used for the microarray analysis (18 samples) and a set consisting of a further 18 samples from an independent set of mice. The following genes were analyzed by RT-PCR to represent both up- and down-regulated genes of varying levels of expression: Ctsd, Serpina3n, Lgals, A2m, Lzp-s, C1qa, C4, Cap1, Fos, Gp49a, Gfap, Erdr1, Ndufs-5, and Mid1 (Fig. 3). The Pearson correlation coefficient between the microarray hybridization data and the quantitative PCR data performed on the original RNA samples was 0.85, and the Pearson correlation coefficient between the original 18 samples and a second independent validation set of 18 samples was 0.83. These results compare favorably with previously published studies [19]. However, in one instance a discordant result was obtained. Mid1, identified as a highly downregulated gene in the SAM analysis, was confirmed to be significantly downregulated in the original sample replication set but not significantly changed in the separate independent sample of 18 mice derived from our colony. The explanation for this finding is not entirely clear; however, it is notable that a high frequency of spontaneous deletion and duplication events affecting the Mid1 gene (which is located in the genetically unstable pseudoautosomal region in *Mus musculus*) has been reported, affecting 20% of paternal chromosomes [20].

**Figure 4:**
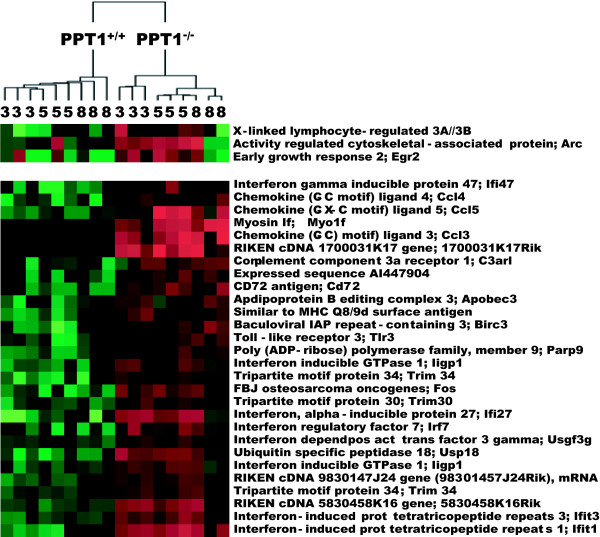
**Hierarchical clustering of significantly regulated genes**. Probe sets represent 18 brain samples from mice at 3, 5, and 8 months of age. For clarity, only representative portions are shown (55 of 255 probe sets corresponding to 227 genes and all downregulated probe sets (12 probe sets corresponding to 8 genes). Upregulated genes are shown in *red *and downregulated genes in *green*. This figure shows the upper quartile of the image. For the full image depicted in Fig. 4 please see Additional File 2. Additional File 10 contains full clustering information for all 227 genes.

Application of an unsupervised clustering algorithm to the 267 probe sets (Fig. 4) correctly distinguished wild-type and knockout groups and to a large extent found greater similarities in gene expression changes in animals with the same age range. The algorithm more sensitively distinguished age classes in the knockout animals as compared to the wild-type, not surprising given the much larger number of differentially expressed genes in the former group. There was a single minor exception in that one 8-month old knockout animal was clustered more closely with the 5-month old knockout animals. This result is consistent with the previously observed slight heterogeneity of onset of neurological signs in the knockout animals at around 5 months of age [11]. Visual inspection of the results of the clustering analysis (Fig. 4, for full version see Additional File 2) revealed several groups of genes with varying patterns of expression with time; for example, several genes peaked early and then decreased to levels below baseline (as in the first three rows of Fig. 4) while other clusters contained genes upregulated late in the course of the disease with highest expression at 8 months. (Particularly notable is the large proportion of interferon-induced genes represented among the list of significantly differentially expressed genes (see, for instance, second panel of Fig. 4)). These interesting patterns of gene expression as revealed by cluster analysis led us to develop individual lists of genes that were significantly regulated at pre-symptomatic (3 month), early symptomatic (5 month) and late symptomatic (8 month) time points.

### Time course of gene expression changes

**Figure 5:**
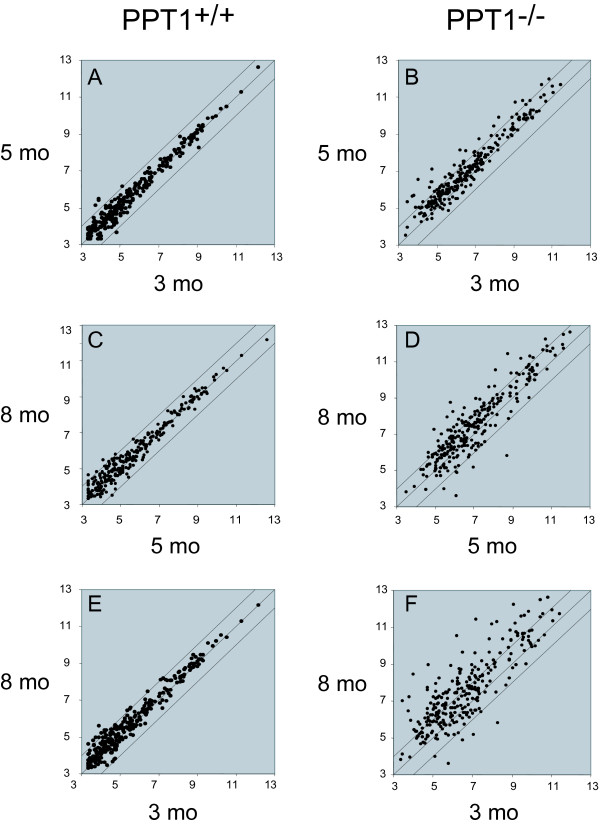
**Scatter plot of gene expression profiles as a function of age**. Pairwise comparisons of age groups within each phenotypic class (control or PPT1 knockout) are shown. Each point represents a unique probe set, and Y- and X-axis values are expressed as the logarithm of expression intensity for each probe set. The data set includes all probe sets (a total of 5236) that passed filtering as described under "Materials and Methods". Note the increased scatter of data points with age in the PPT1 knockout with age as compared to the wild type mice.

**Figure 6:**
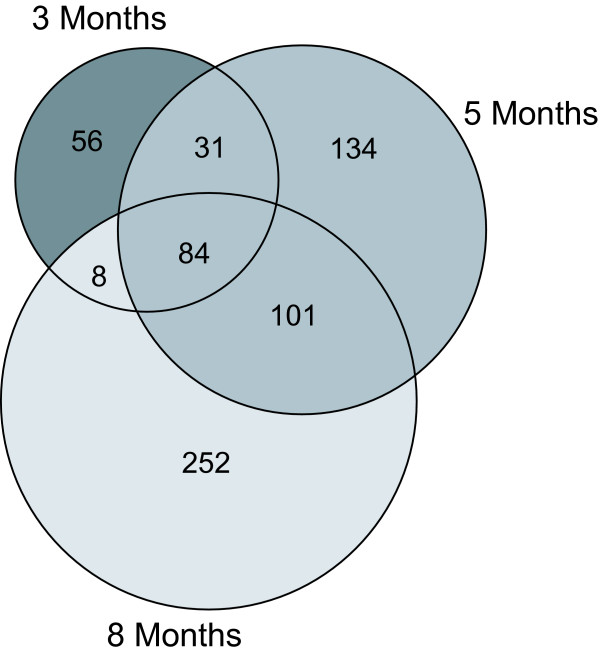
**Venn diagram of gene expression profiles as a function of age**. Comparisons between data sets from wild-type and PPT1 knockout mice at 3, 5 and 8 months of age were made and probe sets having p-values of less than 0.005 (two sample t-tests, random variance version) were considered significant. The resulting gene lists were further used to construct a Venn diagram (in GeneSpring) [57] to identify genes potentially uniquely expressed at the three different ages. Complete gene lists are available in Additional Files 4, 5, and 6.

A comparison of the expression of each differentially expressed gene between wild-type and PPT1 knockout mice as a function of age (Fig. 5) showed that while few genes changed between 3 and 8 months of age in wild-type mice (Fig. 5, panels A, C, and E), comparisons between groups of knockout animals showed a much larger set of genes that changed (particularly increased) during this time (Fig. 5, panels B, D, and F). Groupwise comparisons of wild-type vs. PPT1 knockout mice at 3, 5 and 8 months of age revealed 666 probe sets that were differentially expressed at (at least) one time point. The relationships of these probe sets with respect to age are presented in a Venn diagram (Fig. 6). A complete list of the 84 probe sets (representing 52 genes) common to all three age groups is given in Additional File 3.

We hypothesized that certain subsets as indicated in Fig. 6 might provide particular insights into biological processes affected by PPT1 deficiency. For example, a subset of 56 probe sets (indicated in Fig. 6 in dark shading and listed in full in Additional File 4) were differentially expressed (mainly upregulated) in the knockout at 3 months, but were not identified as significantly changed at either 5 or 8 months in comparison to wild-type. This list of genes was analyzed using the gene ontology function of the program DAVID (The Database for Annotation, Visualization, and Integrated Discovery, 2006) [21], which provides a list of categories of genes that are significantly enriched in an experimental group as compared to a control group. These categories, and a list of representative genes differentially regulated in the PPT1 knockout vs. wild-type mice, are listed in Table 1 (see Additional File 4 for a full list of enriched categories, including uncategorized genes). Interestingly, pattern specification and transcription factors were most highly enriched, and five of these genes have been described as immediate early genes that are induced in response to central nervous system injury (Arc, cysteine-rich protein 61, c-Fos, Jun-b, and the nuclear orphan receptor NR4A1). (A note of caution: one cannot conclude from this analysis that all genes listed in Table 1 are *exclusively *regulated at 3 months; for example, several genes (for example, Cyr61 and c-Fos show -fold changes at 5 mo and 8 mo that are similar to those at 3 months; however, there was sufficient animal-to-animal variability at the later time points that significant regulation at these points could not be confidently predicted given the limited number of animals analyzed. Inspection of the raw data (available for download in the Supplementary Material) does suggest that these changes do decrease with time with respect to Arc, c-fos, NR4A1, and to some extent, Jun-b).

**Table 1:** Enriched categories – genes significantly up- or down-regulated at 3 months (but not significant at 5 or 8 months)

Enriched Category (p-value)	Subcategory	Examples	3 mo	5 mo	8 mo
			(-fold change)		
Pattern Specification (p < 0.0023)	Immediate early genes	^*a*^Arc	3.5	2.7	1.0
		^*a*^Cysteine-rich protein 61	3.0	1.8	4.5
		Zbtb16	-2.4	-1.3	1.8
Transcription (p < 0.015)	Immediate early genes	^*a*^c-Fos	2.9	3.6	2.4
		btg2	2.7	1.2	1.1
		^*a*^NR4A1	2.3	2.1	-1.1
		^*a*^Jun-b	1.7	1.9	1.5
Response to DNA Damage Stimulus (p < 0.021)	DNA replication and stress response	hmg2	2.0	1.3	2.2
		PCNA	-1.8	1.1	1.1
		Sgk1	-2.3	1.1	2.4

^*a*^Reported to be immediate-early response genes expressed in central nervous system

Table 2 lists significantly enriched categories of all genes that were up- or downregulated at least two fold in PPT1 knockout mice at 5 months of age as compared to wild-type (for a full list of categorized and uncategorized genes, see Additional File 5). Immune response genes dominated this list, and included genes that play a role in the inflammatory response, response to pathogens, chemotaxis, antigen presentation, and positive regulation of phagocytosis. Complement components, chemokine ligands, immunoglobulin receptors were prominently upregulated. Protease inhibitors were among the most highly upregulated genes; for example, serpin3An was induced approximately 4, 14 and 20-fold at 3, 5 and 8 months in the PPT1 knockout brain. The expression of cystatin F, α2-macroglobulin, and tissue inhibitor of metalloproteinase (TIMP) were also markedly increased. Genes encoding lysosomal enzymes, such as cathepsins S, Z, and D were each increased several-fold. The one remaining significantly-enriched category, water transport, included genes encoding the water channel aquaporin 4 and podoplanin. Aquaporin 4 [22] is the major water channel in the brain, expressed predominantly in astroglial cells, and plays a major role in fluid clearance in vasogenic brain edema, whereas podoplanin is a lymphatic endothelial cell marker and has been reported to be upregulated in astrocytic tumors [23].

**Table 2:** Enriched categories – genes significantly up- or down-regulated at 5 months

Enriched Category (p-value)	Subcategory	Examples	3 mo	5 mo	8 mo
			(-fold change)		
Immune Response (p < 6.8 × 10^-28^)	Complement	^*a*^C4b	4.8	10.1	8.6
	components	^*a*^C3	1.2	6.5	14.7
		C1q	2.3	2.6	3.2
	Chemotaxis	^*a*^CXCL21	11.4	9.9	6.9
		^*a*^CXCL10	7.5	8.3	6.7
		^*a*^CXCL12	3.4	7.4	4.4
		^*a*^CXCL5	2.3	6.1	2.3
		^*a*^CXCL3	2.4	3.8	2.8
		^*d*^CXCL6	2.9	2.8	1.8
		^*a*^Integrin β2	1.6	3.3	4.6
		^*a*^Integrin αx	2.3	3.8	5.2
	Phagocytosis	^*a*^Clec7a	10.3	13.6	14.7
	Immunoglobulin	^*a*^Fc receptor, IgG	2.7	4.5	6.9
	Receptors				
	Protease inhibitors	^*a*,*b*^Serpin3An	4.3	13.8	20.3
		^*a*^Cystatin F	5.3	12.0	13.6
		α2-macroglobulin	1.3	3.1	5.0
		TIMP	1.5	2.9	6.9
Lysosome (p < 0.0034)	Cathepsins	^*a*^Cathepsin S	2.1	2.4	2.7
		^*a*,*d*^Cathepsin Z	1.7	2.3	2.3
		^*a*^Cathepsin D	1.5	2.1	2.7
Water Transport (p < 0.017)	Aquaporins and related	^*c*,*d*^Aquaporin 4	1.5	2.5	2.4
		Podoplanin	1.6	2.8	2.8

^*a*^Reported to be highly expressed in immune cells

^*b*^Reported to be highly expressed in neuronal cells

^*c*^Reported to be highly expressed in astrocytes

^*d*^Results from one probe set from two or more showing similar results

Table 3 is a list of enriched categories of genes that were significantly regulated (at least two-fold) in PPT1 knockout mouse brain at only the latest time point (8 months), when mice showed clear neurological signs (for a full list containing categorized and uncategorized genes, see Additional File 6). (It should be noted that gene expression changes in some of these genes were observed at 3 or 5 months, but did not reach statistical significance until 8 months). The most significantly enriched category (purine nucleotide metabolism) was comprised of kinases, GTPases, receptors, transporters, and kinesins, perhaps reflecting increased signaling and motility of immune cells in the brain. In support of this idea, most of the upregulated genes in this category are enriched in immune cells (for example ABC1, Gimap4), whereas the downregulated genes are enriched in neural tissue (Uhm kinase 1, EphRa3), observations consistent with the inflammatory cell infiltrate and neuronal loss that characterize late stages of neurodegeneration in neuronal ceroid lipofuscinosis.

**Table 3:** Enriched categories – genes significantly up- or down-regulated at 8 months (but not significant at 3 or 5 months)

Enriched Category (p-value)	Subcategory	Examples	3 mo	5 mo	8 mo
			(-fold change)		
Purine Nucleotide (p < 0.00025)	Kinases	^*a*^v-yes homolog	-1.1	1.5	2.7
		CaM kinase-4	1.2	-1.1	2.5
		B-Raf	1.1	1.1	2.3
		DAP-kinase 1	-1.2	1.3	2.1
		^*b*^Uhm Kinase 1	1.0	-1.3	-2.5
	GTPases	RhoC	1.3	1.5	2.4
		Era1	1.8	-1.1	2.3
		^*a*^Gimap4	2.2	1.1	2.3
	Receptors	EDG3	1.9	2.2	2.5
		^*b*^EphRa3	1.7	-1.0	-2.7
	Transporters	^*a*^ABC1	1.7	1.9	2.3
		^*b*^ABCA8a	-1.6	-1.2	-2.1
	Kinesins	Kinesin 18a	-1.2	1.7	2.1
		^*b*^Kinesin 5a/c	1.0	-1.3	-2.0
Negative Regulators Of Cellular Processes (p < 0.0037)	Transcription Factors	^*a*,*b*^C/EBP-β	1.3	1.4	2.6
		^*a*,*b*^C/EBP-α	1.1	1.4	2.2
		^*a*^Kruppel-like 4	1.3	1.8	2.1
		Midline	-1.9	-1.5	-2.2
		^*b*^Hes5	1.2	-1.1	-3.3
	Growth Factors	^*a*^IGF-1	1.6	1.6	3.2
		^*a*,*b*^CNTF	1.4	1.5	2.8
	Signaling	^*a*^RGS-1	1.2	2.0	5.0
		RGS-2	1.2	1.0	2.2
		^*b*^AA 8 lipoxygenase	-1.5	-1.1	-2.2
Intracellular Transport (p < 0.013)	Proton/Solute Carriers	^*a*,*b*^UCP2	-1.8	-1.0	3.2
		Slc1a3 (glial)	-1.3	1.2	2.6
	Nuclear Transport	Nucleoporin214	-1.0	1.1	2.6
		Ipoll	-1.0	-1.0	2.4
Positive Regulators Of Cellular Processes (p < 0.018)	Transcription Factors	^*b*^Atonal homolog-1	-2.0	1.3	-2.4
		^*a*^NFAT5	1.2	1.1	-2.8
Cell Adhesion (p < 0.018)	Cell surface	^*a*^ICAM	1.8	2.5	5.4
		^*a*^CD44	1.2	2.4	4.5
		Annexin A9	1.7	1.2	3.0
	Extracellular matrix	Procollagen ivα5	1.2	1.7	2.9
		Transglutaminase 2c	1.4	1.6	2.2

^*a*^Reported to be highly expressed in immune cells

^*b*^Reported to be highly expressed in neuronal cells

Some of the most interesting regulated genes at 8 months are those categorized as negative regulators of cellular processes (Table 3). Several of these genes negatively regulate neuronal apoptosis and serve as neuronal survival factors, and therefore may be relevant to the amelioration of Batten disease. Insulin-like growth factor-1 (IGF-1, upregulated about 3-fold) has been noted to increase in concentration in the cerebrospinal fluid of patients with infantile Batten disease [24], and has been used to delay interneuron loss in the *mnd *model of Batten disease [25]. Ciliary neurotrophic factor (CNTF, upregulated 3-fold) is a potent survival factor for neurons and may be important in reducing tissue destruction following inflammatory attacks; its expression is restricted to the nervous system [26]. Death-associated protein kinase 1 (DAPK1, up 2-fold) is expressed in neurons and overexpressed and activated in seizure disorders [27]. DAPK1 seems to have a role in neuronal apoptosis, and has attracted interest as a drug discovery target for neurodegenerative disease [28]. Other genes in this category may have effects on neurons and/or their interactions with inflammatory cells. CCAAT-enhancer binding protein-β (C/EBP-β, up 3-fold) is a transcription factor expressed in neurons that is involved in the transcriptional activation of acute-phase genes and is postulated to control the response to inflammation in neural cells in the brain [29]. It is also implicated in the activation of axonal regeneration-associated genes [30]. Its role may be complex, as it also regulates the expression of IGF-1 in macrophage-derived cell lines [31], and IGF-1 is also a potent mitogen for microglial cells [32]. Finally, Kruppel-like factor 4 (KLF4, up 2-fold) is another transcription factor has a role as a regulator of macrophage activation, but its role in microglial cells in the nervous system has not been previously reported [33].

### Pathway analysis of unfiltered gene sets

**Figure 7:**
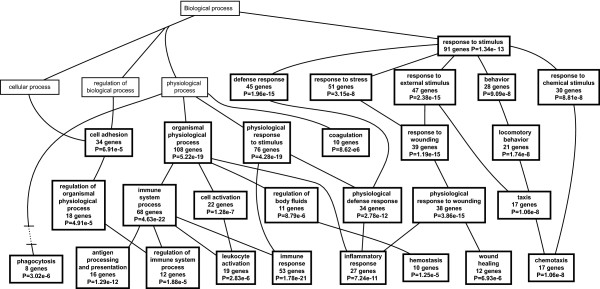
**Directed acyclic graph of gene ontology biological process categories**. A list of 490 significantly regulated genes (from 843 probe sets as determined by SAM prior to filtering) was compared to the entire 49,101 probe set with respect to gene ontology category using the Webgestalt program as described under Methods. Only the significantly enriched (p < 0.001) biological process categories are shown.

**Figure 8:**
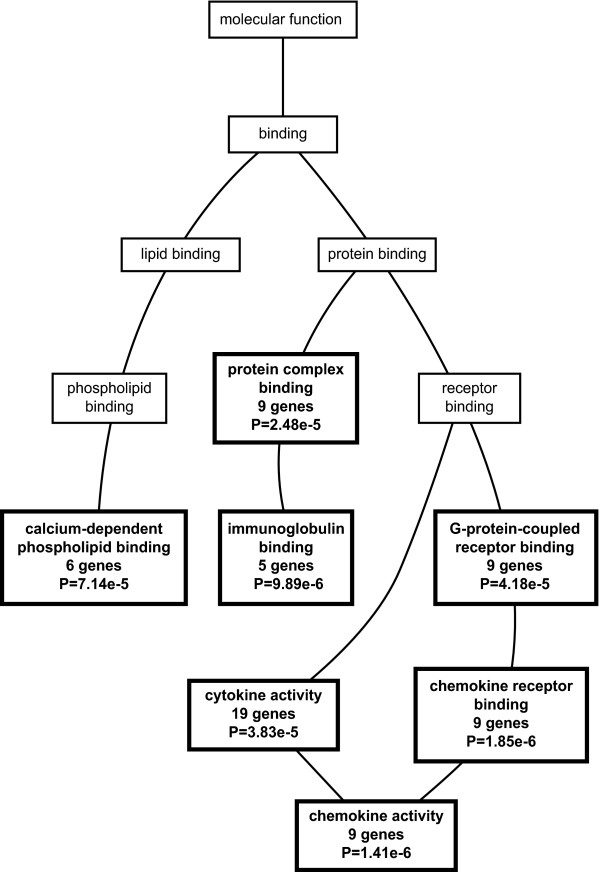
**Directed acyclic graph of gene ontology molecular function categories**. A list of 490 significantly regulated genes (from 843 probe sets as determined by SAM prior to filtering) was compared to the entire 49,101 probe set with respect to gene ontology category using the Webgestalt program as described under Methods. Only the significantly enriched (p < 0.001) molecular function categories are shown.

**Figure 9:**
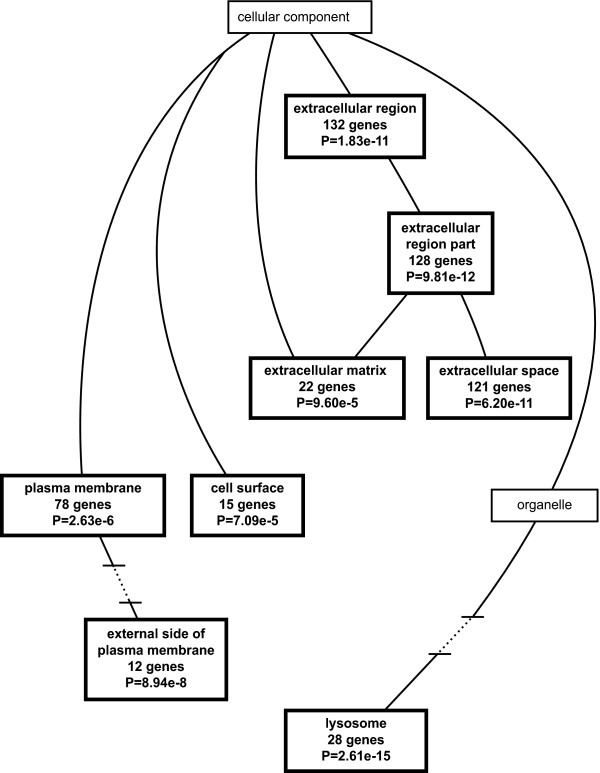
**Directed acyclic graph of gene ontology cellular component categories**. A list of 490 significantly regulated genes (from 843 probe sets as determined by SAM prior to filtering) was compared to the entire 49,101 probe set with respect to gene ontology category using the Webgestalt program as described under Methods. Only the significantly enriched (p < 0.001) cellular component categories are shown.

The genes listed in Tables 1, 2, and 3 were identified using a filtering algorithm that selected for relatively large changes in gene expression between PPT1 knockout and wild-type mice. However, for pathway analysis, smaller changes that occur over larger numbers of gene may provide a more sensitive indication of affected pathways. For this reason, we generated a list of significantly regulated genes (using SAM) that was based on all 45,0101 probe sets prior to filtering (843 probe sets representing 490 genes) and used this list for gene ontology analysis using the Webgestalt program, which displays results in a graphical format (directed acyclic graph) [34]. The results are shown in Fig. 7, 8, 9.

As shown in Fig. 7, a large number of biological processes (27) were enriched, and all of these can be related to immune or inflammatory processes. These include immune response genes (53 genes), leukocyte activation (19 genes), chemotaxis (17 genes), and phagocytosis (8 genes), among others. The molecular function ontology category (Fig. 8) identified significantly enriched categories that were dominated by cytokine and chemokine activity. One interesting category, calcium-dependent phospholipid binding (6 genes) contained five annexins which were upregulated in the brains of PPT1 deficient mice. Annexins play a variety of roles in membrane association events that link calcium signaling and membrane dynamics [35] and participate in modulating signals to phagocytes during apoptotic cell death [36]. The third ontology category, cellular component (Fig. 9), shows enrichment of regulated genes classified as belonging to the extracellular space (121 genes) and lysosome (28 genes). Prominently upregulated members of the extracellular space class included serine protease inhibitors and cytokines. Genes encoding lysosomal proteins are upregulated and are listed in Additional File 7. Enrichment of this category probably reflects recruitment of phagocytic cells (which are rich in lysosomal enzymes) into the brain, and also a compensatory increase in remaining lysosomal enzymes that has been described previously in lysosomal enzyme deficiencies [37].

## Discussion

In the current study, we present a molecular description of gene transcript changes during the course of a severely neurodegenerative disease in whole mouse brain. The changes reflect evolution of an inflammatory disease process with distinct components at early, intermediate and late stages of disease.

It was striking that genes identified as significantly upregulated at 3 months were dominated by those previously classified as immediate-early genes in the central nervous system [38,39]. In our study, these included c-fos, jun-b, btg2, the nuclear orphan receptor NRF4a, Arc, and Cyr61. Immediate early genes are defined as the first group of genes to be expressed (in this case, in neurons) following specific extracellular signals, and are operationally defined as those RNAs expressed in the presence of protein synthesis inhibitors and hence do not require protein synthesis for their transcription. They include regulatory transcription factors, structural and scaffolding proteins, signaling molecules and growth factors, and some of them (such as c-fos and Arc) are now widely used as activity markers for mapping neuronal circuitry [40]. The mechanism for their activation in PPT1 deficiency is not undefined. It has been demonstrated that immediate early gene induction is not consistently coupled to simple metabolic activity or to stress or stimulation [39], and is postulated to play an integrative role in establishing brain circuitry. It is interesting and perhaps unexpected that a lysosomal enzyme deficiency would lead to such changes, suggesting a potential role for the lysosome in the maintenance of neuronal circuitry that may go awry when lysosomal integrity is not maintained.

It is notable that few genes were significantly downregulated in the course of the disorder. One might have expected to see a more striking decline in neuron-specific proteins to parallel the observed neuronal loss described in the disease. One possible explanation is that many of these proteins are expressed only at low levels and in small populations of cells, and so downregulation of these low-level transcripts would not reach statistical significance. However, a significant decline in the transcript encoding the α2 subunit of the GABA-A receptor was noted. This major GABA receptor subunit is highly enriched in cortical and hippocampal pyramidal cells [41], which are major affected cell types in PPT1 deficiency [42]. Hes5 (downregulated 3-fold at 8 months) is a transcription factor involved in notch-mediated neuronal differentiation [43]; its loss perhaps reflects a depletion of neuronal stem cells late in the course of the disease. As expected, transcripts encoding PPT1 were severely depressed. (Unexpected results concerning the CAP1 gene (adenylate cyclase associated protein) were obtained, with up- or down-regulation observed, depending on which probe sets were used for analysis. The 3' untranslated regions of the CAP1 and PPT1 genes partially overlap, suggesting that CAP1 mRNA splicing or stability may have been affected as a consequence of the PPT1 exon 9 disruption, which covers a portion of the 3'UTR. These changes were not examined in further detail).

Gene expression profiles of diseased brain or spinal cord that have been previously reported provide important points for comparison. In a previous study of an infantile Batten disease mouse model (*PPT1*Δexon 4) [14] in which 4000 genes were examined at a single time point (6 months), an upregulation of inflammatory genes concomitant with an inflammation-associated loss of interneurons was observed. A total of 51 genes were found in common (with 5 month time point) [14], suggesting that the findings described here are quite robust. A second more limited study of mouse PPT1 knockout brain at a very early time point (6 weeks) revealed few changes, although an elevation in GFAP was reported [44]. The presence of microglial (macrophage) activation is well documented as a feature of other lysosomal storage disorders. For example, Ohmi, et al. [45] observed prominent microglial infiltration and macrophage activation in mouse models of mucopolysaccharidoses I and IIIb. Many of the most striking gene changes were also observed in our data set: for example, prominent upregulation of CD68/macrosialin, complement components C4 and C1q, lysozyme, cathepsins C, H, S, and Z, DAP12/tyrobp, Mpeg1, serpina3n, and GFAP were reported. In the Sandhoff disease mouse model [46], microglial activation was shown to precede neurodegeneration and was associated with upregulation of macrophage markers CD68/macrosialin, galectin 3, cathepsins S and C, Mpeg1, and glycoprotein49a, which we also observed. Astroglial markers reported in common with our data were seen in Niemann-Pick (acid sphingomyelinase) knockout mice [47], such as GFAP and aquaporin. In this study, serum markers for the disease process were sought, and chemokines and their ligands were considered promising.

A number of chemokines were highly upregulated in the PPT1 deficient mouse brain, with chemokine ligand 21 (Ccl21a/b/c) being upregulated 10-fold. The receptor for Ccl21 ligands, CC chemokine receptor 7 (CCR7), is selectively expressed on mature dendritic cells. Ccl21 facilitates migration of mature dendritic cells from peripheral tissues to regional lymph nodes and enhances receptor-mediated endocytosis by these cells [48]. Its use as a serum marker for disease has not been described; furthermore, it would be interesting to know the effect of specific chemokine antagonism on the neurodegenerative process and whether even non-specific immune suppressants would have an effect on the disease. This point would seem particularly important as neural stem cell therapies are currently under development and current protocols include immunosuppression as part of the treatment. Production of lysophosphatidylcholine (lyso-PC) by cPLA2 in the brain of mice lacking PPT1 has been demonstrated and was proposed as a signal for phagocyte infiltration [49]. In this regard, we note that a lysophospholipid receptor, Edg3, was highly upregulated in our samples, suggesting another avenue for blockade of the immunological response. However, whether or not immune modulation (in the absence of correction of the primary defect) would impact disease progression remains highly speculative at this point.

It should be emphasized that the gene expression changes we observed represent a global pathological process and includes not only changes in the neuronal population but also the infiltration by macrophages and an increase in glial cells. Gene expression changes in PPT1 knockout primary neuronal cultures were recently reported [50]; 106 genes upregulated and 29 downregulated genes were identified. In this study, cholesterol biosynthetic genes were modestly upregulated, a finding supported by a two-fold increase in cholesterol synthetic rate in the cultured cells. In our study, we did observe a striking increase in cholesterol 25-hydroxylase, of about 5-fold. This enzyme is involved in cholesterol metabolism by macrophages, we postulate that it may be needed for membrane turnover and cholesterol disposal in this neurodegenerative process. Cholesterol biosynthesis was not identified as a significantly enriched pathway in our analysis, and the changes in the five cholesterol biosynthetic genes observed in the in vitro study were not significant in our whole brain samples (data not shown).

Several comprehensive microarray-based datasets similar to the current study have recently become available for non-lysosomal neurodegenerative mouse models, such as Huntington disease [51], spinocerebellar ataxia [52], and epilepsy (calcium channelopathy "stargazer" mice) [53]. When we collated these data into BRB Array Tools with the same filtering criteria and normalization methods as described above, surprisingly few genes (only one or two; for example upregulation of the immediate early gene egr2 in Huntington disease and CD52 and GFAP in the "stargazer" mice) were significantly regulated in common with our lysosomal storage disease mouse model (data not shown). Future comparisons between different mouse models of neurodegenerative disease and other disease processes promise to be enlightening as more data become available.

## Conclusion

Gene expression profiling of brains from PPT1 knockout mice showed a number of significant differences from wild-type. Immediate early genes (Arc, Cyr61, c-fos, jun-b, btg2, NR4A1) were significantly up regulated at early stages of the disease. Markers of an intense inflammatory response, noted at 5 and 8 months, may be somewhat unique to PPT1 deficiency and other lysosomal storage diseases and were not shared by several other available neurodegenerative disease models. Chemokine ligands were highly upregulated, and may provide potential serum markers for disease activity. Neuronal survival factors (IGF-1 and CNTF) and a negative regulator of neuronal apoptosis (DAP kinase-1) were upregulated. Few genes were significantly downregulated, which may seem inconsistent with the observed neuronal loss described in the disease. However, significant downregulation of the GABA-A2 receptor was observed, a finding consistent with the loss of GABA-ergic cortical and hippocampal neurons previously described in the disorder. Identification of key negative regulatory molecules in the process of neurodegeneration provides a number of avenues for further exploration and may therefore eventually lead to new therapies.

## Methods

### Brain samples and RNA purification

Homozygous *Ppt1*^*tm*1*Hof*-/- ^mice were maintained in the animal facility at the University of Texas Southwestern Medical Center, Dallas, Texas, USA, as described previously [7]. The knockout was produced through targeted deletion of exon 9 of *PPT1*, which contains a portion of the enzyme active site, and results in no detectable enzyme activity or immunoreactivity [11]. The mice were bred for more than 17 generations onto the C57BL6/J background strain. C57BL6/J mice were from Harlan Sprague Dawley, Inc. (Indianapolis, IN). Sex-matched (except for one discordant pair, see below) knockout and wild-type mice were sacrificed under CO_2 _at 3, 5 and 8 months of age and whole brains were collected in liquid nitrogen and stored at -80°C until RNA extraction was performed. Total RNA was isolated using the RNeasy Lipid Tissue Midi kit (Qiagen, Valencia, CA) and quantitated following the manufacture's guidelines. Three mouse brains were analyzed for each data point (total of 18 determinations).

### cDNA microarrays

Microarrays were hybridized by arrangements through the NIH Neuroscience Microarray Consortium [54]. Affymetrix mouse expression array 430 2.0 chips were used and data was acquired using GCOS v1.4 software (Affymetrix). Signal intensities were scaled to a target intensity of 150 to facilitate interarray comparisons. The intensity level of each gene and a detection call ('Present', 'Absent', or 'Marginally' expressed) were generated using the Statistical Algorithm [55]. All .CEL, .CHP, and signal call files are available for download in Additional File 8, and all raw data are available in the Gene Expression Omnibus [51], record no. GSE6678.

### Filtering and normalization

Probe-set level data generated by GCOS v1.4 software (Affymetrix) was collated into the software package BRB Array Tools, developed by the Biometric Research Branch of the U.S. National Cancer Institute [56]. Spot filter, normalization, and gene filter algorithms were applied to the microarray signal data sequentially. Spot filter threshold values were set to a minimum value (10) if the intensity fell below the minimum. Array normalization was performed by using the median array as the reference array. For certain analysis as indicated in the text, a gene filter was applied that excluded two classes of genes: 1) those with detection call 'Absent' that exceeded 80% of the expression data values and 2) those in which less than 20% of the expression data values had at least a 1.5-fold change in either direction from the median value of that gene.

### Data analysis and statistics

Microarray data analyses were performed using the software package BRB Array Tools [56], which included Cluster 3.0, and TreeView programs. BRB Array Tools was implemented for statistical analysis of microarray data, whereas Cluster 3.0 and TreeView were used for cluster analysis, using the "average linkage" clustering algorithm (similarity of correlation-uncentered). Venn diagrams were generated using the GeneSpring GX 7.3.1 Technology Platform [57]. Functional enriched categories analysis was carried out using DAVID [21] and Webgestalt [34]. Pathway analysis was performed using the MetaCore software by GeneGo [58] and DAVID.

Microarray data were analyzed by Significance Analysis of Microarrays (SAM) (false discovery rate, 0.05, 1000 permutations, confidence level 90%) to identify probe sets differentially expressed in PPT1 knockout as compared to wild-type mice. SAM was applied using either all 45,101 probe sets (resulting in a list of 490 significantly regulated genes) or using 5326 probe sets after filtering (resulting in 267 probe sets representing genes with a higher degree (greater than about 1.5-fold) of regulation. Pathway analysis for the more selective SAM list was performed using the MetaCore software by GeneGo [58]. In addition, two sample t-tests were performed between wild-type and PPT1 knockout mice at 3, 5 and 8 months of age. Probe sets having p-values of less than 0.005 were considered significant. The resulting gene lists were further used to construct a Venn diagram (in GeneSpring) [57] to identify genes overlapping or non-overlapping gene sets significantly expressed at the three different ages.

Unsupervised clustering of the 5326 probe sets from 18 arrays revealed 18 gender-specific genes (all previously reported as sex-linked) and further revealed that one of the nine pairs of animals (from the 8 month group) was discordant for sex. (Each experimental group consisted of one male and two females, with the exception of the 8 month knockout, which had two males and one female). This finding prompted a more detailed analysis to determine the effect of this one instance of gender discrepancy. A t-test using a "leave out one sample" analysis was performed on all combinations for 8-month old mice. In all, six t-test results were generated in this way. The t-test results (yielding lists of significantly regulated genes) were compared by Venn diagram, and were found to be indistinguishable from similar "leave one out" analyses performed using data sets from 3-month-old and 5-month-old mice, suggesting that the one instance of gender mismatch did not have a discernible impact on the overall results.

An initial analysis of functional enriched categories was carried out on the filtered gene set based on the Gene Ontology (GO) database terms using DAVID [21]. Categories with an enrichment score yielding a p-value < 0.05 were considered significant. Analysis of the unfiltered SAM gene set (843 probe sets representing 490 genes), used in the construction of the directed acyclic graphs, was performed using Webgestalt [34] to identify enriched pathways at a significance level of p < 0.001.

### Validation of gene expression changes by quantitative real-time PCR

A sampling of 14 genes from the SAM list of 267 differentially-expressed genes were assayed by quantitative real-time PCR using the fluorescent dye SYBR green I. Two sets of samples were assayed-samples from the original RNA samples used for the microarray analysis (18 samples) and a validation set consisting of a further 18 samples. Total RNA (4 μg) was reverse transcribed with random hexamer primers (Invitrogen) and Superscript II (Invitrogen) according to the directions supplied by the manufacturer. The following genes were analyzed by RT-PCR: Ctsd, Serpina3n, Lgals, A2m, Lzp-s, C1qa, C4, Cap1, Fos, Gp49a, Gfap, ErdrI, Ndufs-3, and MidI. Primers sequences are shown in Additional File 9. The reactions were performed using the 2× Power SYBR^® ^Green PCR Master Mix (Applied Biosystems) and 100–300 nM of primer (see Additional File 9 for details). Assays were performed in triplicate, and analyzed using an ABI 7300 instrument (Applied Biosystems). The primers were either designed using a commercially available program (Primer Express, Applied Biosystems) or obtained from an online database (Primer Bank) [59]. Primer specificity was assessed by analyzing amplicon dissociation curves for each sample. The relative mRNA level was calculated using either the comparative Ct method (if the PCR amplification efficiency of the gene and β-Actin were equivalent) or the relative standard curve method (if the PCR efficiency of the gene and β-Actin were not equivalent), and normalized against one housekeeping gene (β-Actin).

## Authors' contributions

JY and SH designed the study, JY carried out the mice genotyping and brain RNA preparation, XQ designed primers, carried out quantitative real-time PCR validation and performed the data analysis. XQ and SH wrote the paper. All authors read and approved the final manuscript.

## Supplementary Material

Additional File 1 A Microsoft Word table of 267 probe sets representing genes differentially expressed in PPT1 knockout mice. Click here for file

Additional File 2 A pdf file showing the full version of Fig. 4. Hierarchical clustering of significantly regulated genes. Click here for file

Additional File 3 A Microsoft Word table of 84 probe sets representing 52 genes differentially expressed in PPT1 knockout mice at all three time points (3, 5, and 8 months). Click here for file

Additional File 4 **Enriched GO (Gene Ontology) categories (and enrichment scores) for genes significantly regulated at 3 months (but not significant at 5 and 8 months) (Microsoft Word table)**. All genes showing a statistically significant difference between knockout and wild-type at 3 months but not at 5 and 8 months are shown. Genes are first grouped according to Gene Ontology category, and uncategorized genes are also shown. Of note, several genes show evidence of regulation at 5 and 8 months (as well as 3 months), but in these instances, the difference reached statistical significance only at 3 months. Click here for file

Additional File 5 **Enriched GO (Gene Ontology) categories (and enrichment scores) for genes regulated at 5 months (Microsoft Word table)**. For genes represented by more than one probe set, the average of all probe sets was used to calculate the fold change. All genes with expression levels that changed at least two-fold are listed. Genes are first grouped according to Gene Ontology category, and uncategorized genes are also shown. Click here for file

Additional File 6 **Enriched GO (Gene Ontology) categories (and enrichment scores) for genes significantly regulated at 8 months (but not significant at 3 and 5 months) (Microsoft Word table)**. All genes showing a statistically significant difference between knockout and wild-type at 8 months but not at 3 and 5 months are shown. Genes are first grouped according to Gene Ontology category, and uncategorized genes are also shown. Of note, several genes show evidence of regulation at 3 and 5 months (as well as 8 months), but in these instances, the difference reached statistical significance only at 8 months. Click here for file

Additional File 7 Genes encoding lysosomal proteins upregulated in PPT1 knockout brain (Microsoft Word table). Click here for file

Additional File 8 **Signal calls of the microarray generated by GCOS (.zip)**. A compressed file containing the "signalcalls" file generated by GCOS from the microarray. All .CEL and .CHP files from the current study are available for download from the NIH Neuroscience Microarray Consortium (Project ID 370000). Further analyses can be performed by collating data into any available microarray data analysis tool (for example, GCOS, GeneSpring, or the BRB Array tool). The BRB Array tool [56] was used in the current study. Click here for file

Additional File 9 Primer sequences used for validation of microarray data (A Microsoft Word Table). Click here for file

Additional File 10 **Clustering data for significantly regulated genes (.zip)**. Data is viewed with maximum functionality using the free program Java TreeView (available for download [60] under 'File Releases'). The view shown in Fig. 4 was constructed using "TreeView-1.1.0-win.zip". Once Java TreeView is installed, download the attached file Additional File 9, extract all files to a folder, then view the SAM hierarchical clustering by opening the file "SAM genes and samples clustering.cdt". Click here for file
