# Supplementary material for: Gene expression profiling in a mouse model of infantile neuronal ceroid lipofuscinosis reveals upregulation of immediate early genes and mediators of the inflammatory response
Source: BMC Neurosci. 2007 Nov 16;8:95. doi: 10.1186/1471-2202-8-95 (PMC2204004; doi:10.1186/1471-2202-8-95)
Supplement: Additional File 1 — A Microsoft Word table of 267 probe sets representing genes differentially expressed in PPT1 knockout mice. [file 1471-2202-8-95-S1.doc]

| Additional File 1. 267 probe sets representing genes differentially expressed in PPT1 knockout mice by SAM analysis | | | |
| --- | --- | --- | --- |
|  | | | |
| Probe set ID | Gene symbol | Gene Name | Fold-  change |
|  |  |  |  |
| 1420699_at | Clec7a | C-type lectin domain family 7, member a | 12.66 |
| 1419100_at | Serpina3n | serine (or cysteine) peptidase inhibitor, clade A, member 3N | 10.63 |
| 1419202_at | Cst7 | cystatin F (leukocystatin) | 9.52 |
| 1420394_s_at | Gp49a | glycoprotein 49 A | 9.49 |
| 1419426_s_at | Ccl21 | chemokine (C-C motif) ligand 21 | 9.13 |
| 1426808_at | Lgals3 | lectin, galactose binding, soluble 3 | 8.49 |
| 1448303_at | Gpnmb | glycoprotein (transmembrane) nmb | 7.66 |
| 1417461_at | Cap1 | CAP, adenylate cyclase-associated protein 1 (yeast) | 7.63 |
| 1418892_at | Rhoj | ras homolog gene family, member J | 7.49 |
| 1418021_at | C4 | complement component 4 (within H-2S) | 7.47 |
| 1418930_at | Cxcl10 | chemokine (C-X-C motif) ligand 10 | 7.47 |
| 1436996_x_at | Lzp-s | P lysozyme structural | 7.44 |
| 1439426_x_at | Lzp-s | P lysozyme structural | 7.42 |
| 1417462_at | Cap1 | CAP, adenylate cyclase-associated protein 1 (yeast) | 6.54 |
| 1426509_s_at | Gfap | glial fibrillary acidic protein | 6.52 |
| 1427747_a_at | Lcn2 | lipocalin 2 | 6.19 |
| 1423547_at | Lyzs | lysozyme | 6.01 |
| 1447927_at | Mpa2l | macrophage activation 2 like | 5.43 |
| 1424542_at | S100a4 | S100 calcium binding protein A4 | 5.09 |
| 1426508_at | Gfap | glial fibrillary acidic protein | 5.06 |
| 1460218_at | Cd52 | CD52 antigen | 4.98 |
| 1427076_at | Mpeg1 | macrophage expressed gene 1 | 4.88 |
| 1418674_at | Osmr | oncostatin M receptor | 4.88 |
| 1423954_at | C3 | complement component 3 | 4.82 |
| 1419282_at | Ccl12 | chemokine (C-C motif) ligand 12 | 4.78 |
| 1424754_at | Ms4a7 | membrane-spanning 4-domains, subfamily A, member 7 | 4.55 |
| 1452426_x_at |  |  | 4.54 |
| 1440142_s_at | Gfap | Glial fibrillary acidic protein | 4.53 |
| 1438676_at | Mpa2l | macrophage activation 2 like | 4.46 |
| 1447937_a_at | 4933409K07Rik |  | 4.44 |
| 1435477_s_at | Fcgr2b | Fc receptor, IgG, low affinity IIb | 4.33 |
| 1447939_a_at | 4933409K07Rik | RIKEN cDNA 4933409K07 gene | 4.19 |
| 1449363_at | Atf3 | activating transcription factor 3 | 4.10 |
| 1426278_at | Ifi27 | interferon, alpha-inducible protein 27 | 3.96 |
| 1449164_at | Cd68 | CD68 antigen | 3.90 |
| 1450783_at | Ifit1 | interferon-induced protein w/tetratricopeptide repeats 1 | 3.89 |
| 1418240_at | Gbp2 | guanylate nucleotide binding protein 2 | 3.73 |
| 1419128_at | Itgax | integrin alpha X | 3.58 |
| 1450792_at | Tyrobp | TYRO protein tyrosine kinase binding protein | 3.55 |
| 1453196_a_at | Oasl2 | 2'-5' oligoadenylate synthetase-like 2 | 3.54 |
| 1418483_a_at | Ggta1 | glycoprotein galactosyltransferase alpha 1, 3 | 3.45 |
| 1448380_at | Lgals3bp | lectin, galactoside-binding, soluble, 3 binding protein | 3.41 |
| 1427301_at | Cd48 | CD48 antigen | 3.41 |
| 1418191_at | Usp18 | ubiquitin specific peptidase 18 | 3.37 |
| 1436530_at |  | CDNA clone MGC:107680 IMAGE:6766535 | 3.33 |
| 1435906_x_at | Gbp2 | guanylate nucleotide binding protein 2 | 3.31 |
| 1424948_x_at | H2-D1/K1/L | histocompatibility 2, D region locus 1 | 3.31 |
| 1416039_x_at | Cyr61 | cysteine rich protein 61 | 3.29 |
| 1427746_x_at | H2-K1 | histocompatibility 2, K1, K region | 3.29 |
| 1454314_at | 1700031K17Rik | RIKEN cDNA 1700031K17 gene | 3.27 |
| 1442118_at |  | Transcribed locus | 3.27 |
| 1420697_at | Slc15a3 | solute carrier family 15, member 3 | 3.24 |
| 1419004_s_at | Bcl2a1 | B-cell leukemia/lymphoma 2 related protein A1 | 3.19 |
| 1436778_at | Cybb | cytochrome b-245, beta polypeptide | 3.19 |
| 1419728_at | Cxcl5 | chemokine (C-X-C motif) ligand 5 | 3.18 |
| 1455869_at | Camk2b | Calcium/calmodulin-dependent protein kinase II, beta | 3.10 |
| 1423233_at | Cebpd | CCAAT/enhancer binding protein (C/EBP), delta | 3.10 |
| 1460227_at | Timp1 | tissue inhibitor of metalloproteinase 1 | 3.08 |
| 1456292_a_at | Vim | vimentin | 3.08 |
| 1421375_a_at | S100a6 | S100 calcium binding protein A6 (calcyclin) | 3.06 |
| 1438658_a_at | Edg3 | Edg3 | 3.06 |
| 1435036_at | A530050D06Rik | RIKEN cDNA A530050D06 gene | 3.03 |
| 1423555_a_at | Ifi44 | interferon-induced protein 44 | 3.00 |
| 1418126_at | Ccl5 | chemokine (C-C motif) ligand 5 | 3.00 |
| 1418580_at | 5830458K16Rik | RIKEN cDNA 5830458K16 gene | 3.00 |
| 1423760_at | Cd44 | CD44 antigen | 3.00 |
| 1450641_at | Vim | vimentin | 2.98 |
| 1419561_at | Ccl3 | chemokine (C-C motif) ligand 3 | 2.97 |
| 1433935_at | AU020206 | expressed sequence AU020206 | 2.91 |
| 1424067_at | Icam1 | intercellular adhesion molecule | 2.91 |
| 1423100_at | Fos | FBJ osteosarcoma oncogene | 2.91 |
| 1451537_at | Chi3l1 | chitinase 3-like 1 | 2.90 |
| 1452349_x_at | Ifi205 | interferon activated gene 205 | 2.89 |
| 1419132_at | Tlr2 | toll-like receptor 2 | 2.88 |
| 1450678_at | Itgb2 | integrin beta 2 | 2.87 |
| 1447938_at | LOC545608 | similar to RIKEN cDNA 4933409K07 | 2.87 |
| 1438133_a_at | Cyr61 | cysteine rich protein 61 | 2.86 |
| 1421792_s_at | Trem2 | triggering receptor expressed on myeloid cells 2 | 2.83 |
| 1448239_at | Hmox1 | heme oxygenase (decycling) 1 | 2.82 |
| 1456212_x_at | Socs3 | suppressor of cytokine signaling 3 | 2.81 |
| 1425336_x_at | H2-K1 | histocompatibility 2, K1, K region | 2.81 |
| 1437173_at | Edg3 | Edg3 | 2.79 |
| 1429524_at | Myo1f | myosin IF | 2.78 |
| 1434719_at | A2m | alpha-2-macroglobulin | 2.74 |
| 1422875_at | Cd84 | CD84 antigen | 2.73 |
| 1438118_x_at | Vim | vimentin | 2.73 |
| 1418340_at | Fcer1g | Fc receptor, IgE, high affinity I, gamma polypeptide | 2.71 |
| 1424921_at | Bst2 | bone marrow stromal cell antigen 2 | 2.70 |
| 1451931_x_at | H2-D1 | histocompatibility 2, D region locus 1 | 2.70 |
| 1434376_at | Cd44 | CD44 antigen | 2.69 |
| 1423754_at | Ifitm3 | interferon induced transmembrane protein 3 | 2.68 |
| 1418666_at | Ptx3 | pentraxin related gene | 2.67 |
| 1417381_at | C1qa | complement component 1, q subcomponent, alpha polypeptide | 2.67 |
| 1426210_x_at | Parp3 | poly (ADP-ribose) polymerase family, member 3 | 2.66 |
| 1451941_a_at | Fcgr2b | Fc receptor, IgG, low affinity IIb | 2.64 |
| 1449401_at | C1qg | complement component 1, q subcomponent, gamma polypeptide | 2.63 |
| 1442082_at | C3ar1 | complement component 3a receptor 1 | 2.63 |
| 1418675_at | Osmr | oncostatin M receptor | 2.63 |
| 1419599_s_at | Ms4a11 | membrane-spanning 4-domains, subfamily A, member 11 | 2.60 |
| 1422903_at | Ly86 | lymphocyte antigen 86 | 2.60 |
| 1419315_at | Slamf9 | SLAM family member 9 | 2.59 |
| 1449193_at | Cd5l | CD5 antigen-like | 2.55 |
| 1422124_a_at | Ptprc | protein tyrosine phosphatase, receptor type, C | 2.54 |
| 1418392_a_at | Gbp4 | guanylate nucleotide binding protein 4 | 2.52 |
| 1419483_at | C3ar1 | complement component 3a receptor 1 | 2.52 |
| 1421392_a_at | Birc3 | baculoviral IAP repeat-containing 3 | 2.52 |
| 1416295_a_at | Il2rg | interleukin 2 receptor, gamma chain | 2.50 |
| 1449025_at | Ifit3 | interferon-induced protein w/ tetratricopeptide repeat 3 | 2.50 |
| 1416066_at | Cd9 | CD9 antigen | 2.50 |
| 1419598_at | Ms4a6d | membrane-spanning 4-domains, subfamily A, member 6D | 2.49 |
| 1450355_a_at | Capg | capping protein (actin filament), gelsolin-like | 2.47 |
| 1418204_s_at | Aif1 | allograft inflammatory factor 1 | 2.44 |
| 1417244_a_at | Irf7 | interferon regulatory factor 7 | 2.44 |
| 1441926_x_at | Tmie | transmembrane inner ear | 2.42 |
| 1417266_at | Ccl6 | chemokine (C-C motif) ligand 6 | 2.42 |
| 1435144_at |  | Transcribed locus | 2.41 |
| 1429525_s_at | Myo1f | myosin IF | 2.40 |
| 1456251_x_at | Bzrp | benzodiazepine receptor, peripheral | 2.40 |
| 1436058_at | Rsad2 | radical S-adenosyl methionine domain containing 2 | 2.39 |
| 1437726_x_at | C1qb | complement component 1, q beta | 2.39 |
| 1431008_at | 0610037M15Rik | RIKEN cDNA 0610037M15 gene | 2.38 |
| 1417063_at | C1qb | complement component 1, q beta | 2.37 |
| 1448591_at | Ctss | cathepsin S | 2.37 |
| 1427682_a_at | Egr2 | early growth response 2 | 2.37 |
| 1438858_x_at | H2-Aa | Histocompatibility 2, class II antigen A, alpha | 2.37 |
| 1420361_at | Slc11a1 | solute carrier family 11, member 1 | 2.36 |
| 1452483_a_at | Cd44 | CD44 antigen | 2.35 |
| 1449289_a_at | B2m | beta-2 microglobulin | 2.35 |
| 1424965_at | Lpxn | leupaxin | 2.35 |
| 1435290_x_at | H2-Aa | histocompatibility 2, class II antigen A, alpha | 2.34 |
| 1427994_at | Cd300lf | CD300 antigen like family member F | 2.33 |
| 1419209_at | Cxcl1 | chemokine (C-X-C motif) ligand 1 | 2.32 |
| 1419309_at | Pdpn | podoplanin | 2.31 |
| 1455899_x_at | Socs3 | suppressor of cytokine signaling 3 | 2.31 |
| 1436779_at | Cybb | cytochrome b-245, beta polypeptide | 2.30 |
| 1449388_at | Thbs4 | thrombospondin 4 | 2.30 |
| 1418536_at | LOC386462 | similar to MHC Q8/9d surface antigen | 2.29 |
| 1426112_a_at | Cd72 | CD72 antigen | 2.27 |
| 1454268_a_at | Cyba | cytochrome b-245, alpha polypeptide | 2.27 |
| 1451860_a_at | Trim30 | tripartite motif protein 30 | 2.25 |
| 1417601_at | Rgs1 | regulator of G-protein signaling 1 | 2.25 |
| 1418365_at | Ctsh | cathepsin H | 2.24 |
| 1421550_a_at | Trim34 | tripartite motif protein 34 | 2.23 |
| 1451683_x_at | H2-D1 | Histocompatibility 2, D region | 2.22 |
| 1452428_a_at | B2m | beta-2 microglobulin | 2.22 |
| 1435665_at | AI451617 | expressed sequence AI451617 | 2.22 |
| 1445247_at | C530044C16Rik | RIKEN cDNA C530044C16 gene | 2.19 |
| 1422476_at | Ifi30 | interferon gamma inducible protein 30 | 2.19 |
| 1460661_at | Edg3 | Edg3 | 2.18 |
| 1448640_at | Slc14a1 | solute carrier family 14 (urea transporter), member 1 | 2.18 |
| 1438980_x_at | 4732466D17Rik | RIKEN cDNA 4732466D17 gene | 2.17 |
| 1426260_a_at | Ugt1locus |  | 2.16 |
| 1443814_x_at | Ctsh | cathepsin H | 2.14 |
| 1448160_at | Lcp1 | lymphocyte cytosolic protein 1 | 2.14 |
| 1422781_at | Tlr3 | toll-like receptor 3 | 2.13 |
| 1448891_at | Msr2 | macrophage scavenger receptor 2 | 2.12 |
| 1425382_a_at | Aqp4 | aquaporin 4 | 2.12 |
| 1418687_at | Arc | activity regulated cytoskeletal-associated protein | 2.11 |
| 1450637_a_at | Aebp1 | AE binding protein 1 | 2.11 |
| 1417928_at | Pdlim4 | PDZ and LIM domain 4 | 2.10 |
| 1419042_at | Iigp1 | interferon inducible GTPase 1 | 2.10 |
| 1419043_a_at | Iigp1 | interferon inducible GTPase 1 | 2.10 |
| 1426936_at | LOC433593 | hypothetical LOC433593 | 2.10 |
| 1420357_s_at | Xlr3a /// Xlr3b | X-linked lymphocyte-regulated 3A ///3B | 2.10 |
| 1425964_x_at | Hspb1 | heat shock protein 1 | 2.09 |
| 1417870_x_at | Ctsz | cathepsin Z | 2.09 |
| 1416625_at | Serping1 | serine (or cysteine) peptidase inhibitor G1 | 2.09 |
| 1456567_x_at | Grn | granulin | 2.08 |
| 1436332_at | Hspb6 | heat shock protein, alpha-crystallin-related, B6 | 2.07 |
| 1415983_at | Lcp1 | lymphocyte cytosolic protein 1 | 2.07 |
| 1439831_at |  | Transcribed locus | 2.06 |
| 1424176_a_at | Anxa4 | annexin A4 | 2.06 |
| 1448118_a_at | Ctsd | cathepsin D | 2.06 |
| 1451777_at | BC013672 | cDNA sequence BC013672 | 2.05 |
| 1428114_at | Slc14a1 | solute carrier family 14 (urea transporter), member 1 | 2.05 |
| 1421374_a_at | Fxyd1 | FXYD domain-containing ion transport regulator 1 | 2.05 |
| 1438948_x_at | Bzrp | benzodiazepine receptor, peripheral | 2.04 |
| 1417523_at | Plek | pleckstrin | 2.04 |
| 1417868_a_at | Ctsz | cathepsin Z | 2.04 |
| 1426169_a_at | Lat2 | linker for activation of T cells family, member 2 | 2.04 |
| 1421578_at | Ccl4 | chemokine (C-C motif) ligand 4 | 2.03 |
| 1422808_s_at | Dock2 | dedicator of cyto-kinesis 2 | 2.03 |
| 1417292_at | Ifi47 | interferon gamma inducible protein 47 | 2.03 |
| 1455332_x_at | Fcgr2b | Fc receptor, IgG, low affinity IIb | 2.02 |
| 1455050_at | E130203B14Rik | RIKEN cDNA E130203B14 gene | 2.02 |
| 1435330_at | AI447904 | expressed sequence AI447904 | 2.02 |
| 1438855_x_at | Tnfaip2 | tumor necrosis factor, alpha-induced protein 2 | 2.02 |
| 1452014_a_at | Igf1 | insulin-like growth factor 1 | 2.01 |
| 1449347_a_at | Xlr4b /// Xlr4a | X-linked lymphocyte-regulated 4B / 4A | 2.01 |
| 1441811_x_at | 0610011I04Rik | RIKEN cDNA 0610011I04 gene | 2.00 |
| 1443698_at | Fbxo39 | F-box protein 39 | 2.00 |
| 1419814_s_at | S100a1 | S100 calcium binding protein A1 | 2.00 |
| 1451784_x_at | H2-D1 /K1 /L | histocompatibility 2, D region locus 1 / | 2.00 |
| 1459961_a_at |  |  | 2.00 |
| 1434380_at | 9830147J24Rik | RIKEN cDNA 9830147J24 gene | 1.99 |
| 1416897_at | Parp9 | poly (ADP-ribose) polymerase family, member 9 | 1.99 |
| 1416226_at | Arpc1b | actin related protein 2/3 complex, subunit 1B | 1.99 |
| 1432331_a_at | Prrx2 | paired related homeobox 2 | 1.99 |
| 1434503_s_at | Lamp2 | lysosomal membrane glycoprotein 2 | 1.99 |
| 1425214_at | P2ry6 | pyrimidinergic receptor P2Y, G-protein coupled, 6 | 1.99 |
| 1454757_s_at | D12Ertd647e | DNA segment, Chr 12, ERATO Doi 647, expressed | 1.99 |
| 1417961_a_at | Trim30 | tripartite motif protein 30 | 1.98 |
| 1425603_at | 0610011I04Rik | RIKEN cDNA 0610011I04 gene | 1.98 |
| 1451905_a_at | Mx1 | myxovirus (influenza virus) resistance 1 | 1.98 |
| 1434449_at | Aqp4 | aquaporin 4 | 1.98 |
| 1418932_at | Nfil3 | nuclear factor, interleukin 3, regulated | 1.98 |
| 1417470_at | Apobec3 | apolipoprotein B editing complex 3 | 1.98 |
| 1451969_s_at | Parp3 | poly (ADP-ribose) polymerase family, member 3 | 1.98 |
| 1450107_a_at | Renbp | renin binding protein | 1.98 |
| 1425545_x_at | H2-D1 /H2-L | histocompatibility 2, D region locus 1 | 1.97 |
| 1417869_s_at | Ctsz | cathepsin Z | 1.97 |
| 1439066_at | Angpt1 | angiopoietin 1 | 1.97 |
| 1443253_at |  |  | 1.96 |
| 1456494_a_at | Trim30 | tripartite motif protein 30 | 1.96 |
| 1451755_a_at | Apobec1 | apolipoprotein B editing complex 1 | 1.96 |
| 1430295_at | Gna13 | guanine nucleotide binding protein, alpha 13 | 1.96 |
| 1419768_at | Cd22 | CD22 antigen | 1.96 |
| 1419482_at | C3ar1 | complement component 3a receptor 1 | 1.96 |
| 1436905_x_at | Laptm5 | lysosomal-associated protein transmembrane 5 | 1.96 |
| 1448620_at | Fcgr3 | Fc receptor, IgG, low affinity III | 1.96 |
| 1448710_at | Cxcr4 | chemokine (C-X-C motif) receptor 4 | 1.95 |
| 1449297_at | Casp12 | caspase 12 | 1.95 |
| 1438936_s_at | Ang1 | angiogenin, ribonuclease A family, member 1 | 1.95 |
| 1448021_at |  | Transcribed locus | 1.94 |
| 1422962_a_at | Psmb8 | proteosome (prosome, macropain) subunit, beta type 8 | 1.94 |
| 1421840_at | Abca1 | ATP-binding cassette, sub-family A (ABC1), member 1 | 1.94 |
| 1417104_at | Emp3 | epithelial membrane protein 3 | 1.94 |
| 1451416_a_at | Tgm1 | transglutaminase 1, K polypeptide | 1.94 |
| 1427140_at | Pvt1 | plasmacytoma variant translocation 1 | 1.93 |
| 1421839_at | Abca1 | ATP-binding cassette, sub-family A (ABC1), member 1 | 1.93 |
| 1418825_at | Irgm | immunity-related GTPase family, M | 1.93 |
| 1430390_x_at | 4930444G20Rik | RIKEN cDNA 4930444G20 gene | 1.93 |
| 1445897_s_at | Ifi35 | interferon-induced protein 35 | 1.92 |
| 1447745_at | Aqp4 | aquaporin 4 | 1.92 |
| 1437155_a_at | Wwtr1 | WW domain containing transcription regulator 1 | 1.91 |
| 1453145_at | 4933439C20Rik | RIKEN cDNA 4933439C20 gene | 1.91 |
| 1452231_x_at | LOC545386 | similar to Interferon-activatable protein 205 (IFI-205) | 1.91 |
| 1460351_at | S100a11 | S100 calcium binding protein A11 (calizzarin) | 1.91 |
| 1452956_a_at | D12Ertd647e | DNA segment, Chr 12, ERATO Doi 647, expressed | 1.90 |
| 1455768_at | Npc2 | Niemann Pick type C2 | 1.90 |
| 1416695_at | Bzrp | benzodiazepine receptor, peripheral | 1.90 |
| 1453011_at | Dhrs6 | dehydrogenase/reductase (SDR family) member 6 | 1.90 |
| 1434366_x_at | C1qb | complement component 1, q beta | 1.89 |
| 1418981_at | Casp12 | caspase 12 | 1.88 |
| 1417821_at | D17H6S56E-5 | DNA segment, Chr 17, human D6S56E 5 | 1.88 |
| 1429235_at | Galntl2 | UDP-N-acetyl-alpha-D-galactosamine:polypeptide N-acetylgalactosaminyltransferase-like 2 | 1.87 |
| 1424857_a_at | Trim34 | tripartite motif protein 34 | 1.87 |
| 1422571_at | Thbs2 | thrombospondin 2 | 1.87 |
| 1448748_at | Plek | pleckstrin | 1.85 |
| 1420413_at | Slc7a11 | solute carrier family 7 member 11 | 1.85 |
| 1423909_at | 0610011I04Rik | RIKEN cDNA 0610011I04 gene | 1.84 |
| 1416382_at | Ctsc | cathepsin C | 1.83 |
| 1421322_a_at | Isgf3g | interferon dependent positive acting transcription factor 3 gamma | 1.82 |
| 1450379_at | Msn | moesin | 1.82 |
| 1421217_a_at | Lgals9 | lectin, galactose binding, soluble 9 | 1.81 |
| 1416494_at | Ndufs5 | NADH dehydrogenase (ubiquinone) Fe-S protein 5 | 0.45 |
| 1455444_at | Gabra2 | GABA-A receptor, subunit alpha 2 | 0.42 |
| 1421738_at | Gabra2 | GABA-A receptor, subunit alpha 2 | 0.40 |
| 1443865_at | Gabra2 | GABA-A receptor, subunit alpha 2 | 0.40 |
| 1452406_x_at | Edr1 | erythroid differentiation regulator 1 | 0.33 |
| 1427820_at |  | Mus musculus, clone IMAGE:3983821 | 0.32 |
| 1443141_at | Cap1 | CAP, adenylate cyclase-associated protein 1 (yeast) | 0.31 |
| 1438239_at | Mid1 | midline 1 | 0.19 |
| 1422468_at | Ppt1 | palmitoyl-protein thioesterase 1 | 0.05 |
| 1422467_at | Ppt1 | palmitoyl-protein thioesterase 1 | 0.02 |
