# Supplementary material for: Gene expression profiling in a mouse model of infantile neuronal ceroid lipofuscinosis reveals upregulation of immediate early genes and mediators of the inflammatory response
Source: BMC Neurosci. 2007 Nov 16;8:95. doi: 10.1186/1471-2202-8-95 (PMC2204004; doi:10.1186/1471-2202-8-95)
Supplement: Additional File 3 — A Microsoft Word table of 84 probe sets representing 52 genes differentially expressed in PPT1 knockout mice at all three time points (3, 5, and 8 months). [file 1471-2202-8-95-S3.doc]

| Additional File 3. A list of genes significantly regulated in PPT1 knockout brain at all three time points (3, 5, and 8 months). | | | | | |
| --- | --- | --- | --- | --- | --- |
| Gene ID | Gene Symbol | Gene Name | Fold-change | | |
|  |  |  | 3 mo | 5 mo | 8 mo |
| [20716](http://bioinfo.vanderbilt.edu/webgestalt/llid_info.php?llid=20716) | Serpina3n | serine (or cysteine) peptidase inhibitor, clade A, member 3N | 4.3 | 13.6 | 20.3 |
| [13011](http://bioinfo.vanderbilt.edu/webgestalt/llid_info.php?llid=13011) | Cst7 | cystatin F (leukocystatin) | 5.3 | 12.1 | 13.6 |
| [80837](http://bioinfo.vanderbilt.edu/webgestalt/llid_info.php?llid=80837) | Rhoj | ras homolog gene family, member J | 5.6 | 5.4 | 12.3 |
| [14580](http://bioinfo.vanderbilt.edu/webgestalt/llid_info.php?llid=14580) | Gfap | glial fibrillary acidic protein | 3.6 | 7.1 | 10.2 |
| [18414](http://bioinfo.vanderbilt.edu/webgestalt/llid_info.php?llid=18414) | Osmr | oncostatin M receptor | 2.7 | 4.5 | 9.1 |
| [12331](http://bioinfo.vanderbilt.edu/webgestalt/llid_info.php?llid=12331) | Cap1 | *CAP, adenylate cyclase-associated protein 1 (yeast) | 6.5 | 8.3 | 7.8 |
| [14130](http://bioinfo.vanderbilt.edu/webgestalt/llid_info.php?llid=14130) | Fcgr2b | Fc receptor, IgG, low affinity IIb | 2.6 | 4.5 | 6.9 |
| [15945](http://bioinfo.vanderbilt.edu/webgestalt/llid_info.php?llid=15945) | Cxcl10 | chemokine (C-X-C motif) ligand 10 | 7.5 | 8.3 | 6.7 |
| [22352](http://bioinfo.vanderbilt.edu/webgestalt/llid_info.php?llid=22352) | Vim | vimentin | 1.8 | 3.1 | 5.6 |
| [16411](http://bioinfo.vanderbilt.edu/webgestalt/llid_info.php?llid=16411) | Itgax | integrin alpha X | 2.3 | 3.8 | 5.2 |
| [12505](http://bioinfo.vanderbilt.edu/webgestalt/llid_info.php?llid=12505) | Cd44 | CD44 antigen | 1.6 | 3.3 | 4.9 |
| [12514](http://bioinfo.vanderbilt.edu/webgestalt/llid_info.php?llid=12514) | Cd68 | CD68 antigen | 2.4 | 5.1 | 4.8 |
| [12654](http://bioinfo.vanderbilt.edu/webgestalt/llid_info.php?llid=12654) | Chi3l1 | chitinase 3-like 1 | 2.0 | 2.7 | 4.6 |
| [20200](http://bioinfo.vanderbilt.edu/webgestalt/llid_info.php?llid=20200) | S100a6 | S100 calcium binding protein A6 (calcyclin) | 1.8 | 3.4 | 4.6 |
| [23833](http://bioinfo.vanderbilt.edu/webgestalt/llid_info.php?llid=23833) | Cd52 | CD52 antigen | 4.5 | 6.1 | 4.5 |
| [20293](http://bioinfo.vanderbilt.edu/webgestalt/llid_info.php?llid=20293) | Ccl12 | chemokine (C-C motif) ligand 12 | 3.4 | 7.4 | 4.4 |
| [11910](http://bioinfo.vanderbilt.edu/webgestalt/llid_info.php?llid=11910) | Atf3 | activating transcription factor 3 | 3.8 | 4.9 | 3.7 |
| [22177](http://bioinfo.vanderbilt.edu/webgestalt/llid_info.php?llid=22177) | Tyrobp | TYRO protein tyrosine kinase binding protein | 3.1 | 3.9 | 3.7 |
| [235587](http://bioinfo.vanderbilt.edu/webgestalt/llid_info.php?llid=235587) | Parp3 | poly (ADP-ribose) polymerase family, member 3 | 2.3 | 2.3 | 3.6 |
| [19039](http://bioinfo.vanderbilt.edu/webgestalt/llid_info.php?llid=19039) | Lgals3bp | lectin, galactoside-binding, soluble, 3 binding protein | 3.1 | 3.8 | 3.4 |
| [12259](http://bioinfo.vanderbilt.edu/webgestalt/llid_info.php?llid=12259) | C1qa | complement component 1, q subcomponent, alpha polypeptide | 2.3 | 2.6 | 3.3 |
| [12262](http://bioinfo.vanderbilt.edu/webgestalt/llid_info.php?llid=12262) | C1qc | complement component 1, q subcomponent, C chain | 2.2 | 2.5 | 3.3 |
| [14127](http://bioinfo.vanderbilt.edu/webgestalt/llid_info.php?llid=14127) | Fcer1g | Fc receptor, IgE, high affinity I, gamma polypeptide | 2.2 | 2.6 | 3.3 |
| [12527](http://bioinfo.vanderbilt.edu/webgestalt/llid_info.php?llid=12527) | Cd9 | CD9 antigen | 1.8 | 2.7 | 3.2 |
| [14594](http://bioinfo.vanderbilt.edu/webgestalt/llid_info.php?llid=14594) | Ggta1 | glycoprotein galactosyltransferase alpha 1, 3 | 2.8 | 4.7 | 3.2 |
| [12260](http://bioinfo.vanderbilt.edu/webgestalt/llid_info.php?llid=12260) | C1qb | complement component 1, q subcomponent, beta polypeptide | 2.0 | 2.4 | 3.1 |
| [65221](http://bioinfo.vanderbilt.edu/webgestalt/llid_info.php?llid=65221) | Slc15a3 | solute carrier family 15, member 3 | 3.3 | 3.3 | 3.1 |
| [66058](http://bioinfo.vanderbilt.edu/webgestalt/llid_info.php?llid=66058) | Tmem176a | transmembrane protein 176A | 1.6 | 2.1 | 3.1 |
| [14469](http://bioinfo.vanderbilt.edu/webgestalt/llid_info.php?llid=14469) | Gbp2 | guanylate nucleotide binding protein 2 | 4.3 | 4.3 | 3.0 |
| [12258](http://bioinfo.vanderbilt.edu/webgestalt/llid_info.php?llid=12258) | Serping1 | serine (or cysteine) peptidase inhibitor, clade G, member 1 | 1.7 | 1.9 | 2.9 |
| [11303](http://bioinfo.vanderbilt.edu/webgestalt/llid_info.php?llid=11303) | Abca1 | ATP-binding cassette, sub-family A (ABC1), member 1 | 1.4 | 1.9 | 2.8 |
| [13036](http://bioinfo.vanderbilt.edu/webgestalt/llid_info.php?llid=13036) | Ctsh | cathepsin H | 2.0 | 2.0 | 2.8 |
| [14726](http://bioinfo.vanderbilt.edu/webgestalt/llid_info.php?llid=14726) | Pdpn | podoplanin | 1.6 | 2.7 | 2.8 |
| [13033](http://bioinfo.vanderbilt.edu/webgestalt/llid_info.php?llid=13033) | Ctsd | cathepsin D | 1.5 | 2.1 | 2.7 |
| [13040](http://bioinfo.vanderbilt.edu/webgestalt/llid_info.php?llid=13040) | Ctss | cathepsin S | 2.1 | 2.4 | 2.7 |
| [66141](http://bioinfo.vanderbilt.edu/webgestalt/llid_info.php?llid=66141) | Ifitm3 | interferon induced transmembrane protein 3 | 2.6 | 2.7 | 2.7 |
| [240327](http://bioinfo.vanderbilt.edu/webgestalt/llid_info.php?llid=240327) | EG240327 | predicted gene, EG240327 | 1.7 | 2.0 | 2.6 |
| [56188](http://bioinfo.vanderbilt.edu/webgestalt/llid_info.php?llid=56188) | Fxyd1 | FXYD domain-containing ion transport regulator 1 | 1.5 | 2.3 | 2.6 |
| [64138](http://bioinfo.vanderbilt.edu/webgestalt/llid_info.php?llid=64138) | Ctsz | cathepsin Z | 1.8 | 2.3 | 2.6 |
| [12870](http://bioinfo.vanderbilt.edu/webgestalt/llid_info.php?llid=12870) | Cp | ceruloplasmin | 2.5 | 1.9 | 2.5 |
| [14824](http://bioinfo.vanderbilt.edu/webgestalt/llid_info.php?llid=14824) | Grn | granulin | 1.7 | 2.1 | 2.5 |
| [20193](http://bioinfo.vanderbilt.edu/webgestalt/llid_info.php?llid=20193) | S100a1 | S100 calcium binding protein A1 | 1.6 | 1.9 | 2.5 |
| [11758](http://bioinfo.vanderbilt.edu/webgestalt/llid_info.php?llid=11758) | Prdx6 | peroxiredoxin 6 | 1.3 | 1.7 | 2.3 |
| [12010](http://bioinfo.vanderbilt.edu/webgestalt/llid_info.php?llid=12010) | B2m | beta-2 microglobulin | 2.4 | 2.4 | 2.3 |
| [30794](http://bioinfo.vanderbilt.edu/webgestalt/llid_info.php?llid=30794) | Pdlim4 | PDZ and LIM domain 4 | 2.0 | 2.1 | 2.2 |
| [17084](http://bioinfo.vanderbilt.edu/webgestalt/llid_info.php?llid=17084) | Ly86 | lymphocyte antigen 86 | 2.7 | 3.1 | 2.1 |
| [320736](http://bioinfo.vanderbilt.edu/webgestalt/llid_info.php?llid=320736) | E130203B14Rik | RIKEN cDNA E130203B14 gene | 2.0 | 2.0 | 2.0 |
| [16852](http://bioinfo.vanderbilt.edu/webgestalt/llid_info.php?llid=16852) | Lgals1 | lectin, galactose binding, soluble 1 | 1.6 | 1.9 | 1.9 |
| [70110](http://bioinfo.vanderbilt.edu/webgestalt/llid_info.php?llid=70110) | Ifi35 | interferon-induced protein 35 | 2.0 | 2.0 | 1.8 |
| [17698](http://bioinfo.vanderbilt.edu/webgestalt/llid_info.php?llid=17698) | Msn | moesin | 1.9 | 1.8 | 1.7 |
| [14395](http://bioinfo.vanderbilt.edu/webgestalt/llid_info.php?llid=14395) | Gabra2 | gamma-aminobutyric acid (GABA-A) receptor, subunit alpha 2 | 0.44 | 0.39 | .36 |
| [19063](http://bioinfo.vanderbilt.edu/webgestalt/llid_info.php?llid=19063) | Ppt1 | palmitoyl-protein thioesterase 1 | .03 | .039 | .053 |
