# Supplementary material for: Gene expression profiling in a mouse model of infantile neuronal ceroid lipofuscinosis reveals upregulation of immediate early genes and mediators of the inflammatory response
Source: BMC Neurosci. 2007 Nov 16;8:95. doi: 10.1186/1471-2202-8-95 (PMC2204004; doi:10.1186/1471-2202-8-95)
Supplement: Additional File 4 — Enriched GO (Gene Ontology) categories (and enrichment scores) for genes significantly regulated at 3 months (but not significant at 5 and 8 months) (Microsoft Word table). All genes showing a statistically significant difference between knockout and wild-type at 3 months but not at 5 and 8 months are shown. Genes are first grouped according to Gene Ontology category, and uncategorized genes are also shown. Of note, several genes show evidence of regulation at 5 and 8 months (as well as 3 months), but in these instances, the difference reached statistical significance only at 3 months. [file 1471-2202-8-95-S4.doc]

| Additional File 4 |  |  |  |  |
| --- | --- | --- | --- | --- |
| **Enriched GO (Gene Ontology) categories (and enrichment scores) for genes significantly regulated at 3 months (but not significant at 5 and 8 months)** | | | | |
| ***Gene family and gene name*** | ***Affymetrix-ID*** | ***Fold change KO:WT*** | | |
|  |  | ***3 mo*** | ***5 mo*** | ***8 mo*** |
| ***Transcription (1.5E-2)*** | |  |  |  |
| [fbj osteosarcoma oncogene](http://niaid.abcc.ncifcrf.gov/geneReportFull.jsp?rowids=3873) | 1423100_at | ***2.85*** | 3.57 | 2.42 |
| [b-cell translocation gene 2, anti-proliferative](http://niaid.abcc.ncifcrf.gov/geneReportFull.jsp?rowids=14999) | 1416250_at | ***2.73*** | 1.22 | 1.11 |
| [nuclear receptor subfamily 4, group a, member 1](http://niaid.abcc.ncifcrf.gov/geneReportFull.jsp?rowids=37717) | 1416505_at | ***2.31*** | 2.08 | -1.07 |
| [high mobility group box 2](http://niaid.abcc.ncifcrf.gov/geneReportFull.jsp?rowids=5296) | 1452534_a_at | ***2.02*** | 1.34 | 2.19 |
| [myeloid ecotropic viral integration site 1](http://niaid.abcc.ncifcrf.gov/geneReportFull.jsp?rowids=149112) | 1445773_at | ***2.00*** | 1.06 | 0.72 |
| [jun-b oncogene](http://niaid.abcc.ncifcrf.gov/geneReportFull.jsp?rowids=6767) | 1415899_at | ***1.74*** | 1.89 | 1.49 |
| [zinc finger protein 108](http://niaid.abcc.ncifcrf.gov/geneReportFull.jsp?rowids=148015) | 1421454_at | ***-1.38*** | -1.61 | -1.28 |
| [sex comb on midleg homolog 1](http://niaid.abcc.ncifcrf.gov/geneReportFull.jsp?rowids=4264) | 1441573_at | ***-1.53*** | -1.71 | -1.41 |
| [bromodomain containing 7](http://niaid.abcc.ncifcrf.gov/geneReportFull.jsp?rowids=144008) | 1436469_at | ***-1.66*** | -1.32 | 1.79 |
| [tsc22 domain family 3](http://niaid.abcc.ncifcrf.gov/geneReportFull.jsp?rowids=27494) | 1420772_a_at | ***-2.00*** | -1.57 | 1.31 |
| [period homolog 2 (drosophila)](http://niaid.abcc.ncifcrf.gov/geneReportFull.jsp?rowids=39617) | 1417603_at | ***-2.12*** | -1.32 | -1.65 |
| [bobby sox homolog (drosophila)](http://niaid.abcc.ncifcrf.gov/geneReportFull.jsp?rowids=144137) | 1458602_at | ***-2.22*** | -1.04 | -1.60 |
| [zinc finger and btb domain containing 16](http://niaid.abcc.ncifcrf.gov/geneReportFull.jsp?rowids=149025) | 1442025_a_at | ***-2.38*** | -1.33 | 1.80 |
| ***Pattern Specification (2.3E-3) (embryonic pattern specification)*** | |  |  |  |
| [activity regulated cytoskeletal-associated protein](http://niaid.abcc.ncifcrf.gov/geneReportFull.jsp?rowids=144448) | 1418687_at | ***3.46*** | 2.73 | 1.00 |
| [cysteine rich protein 61](http://niaid.abcc.ncifcrf.gov/geneReportFull.jsp?rowids=24861) | 1438133_a_at | ***2.96*** | 1.75 |  |
| [b-cell translocation gene 2, anti-proliferative](http://niaid.abcc.ncifcrf.gov/geneReportFull.jsp?rowids=14999) | 1416250_at | ***2.73*** | 1.22 | 1.11 |
| [chemokine (c-x-c motif) receptor 4](http://niaid.abcc.ncifcrf.gov/geneReportFull.jsp?rowids=37712) | 1448710_at | ***2.56*** | 1.78 | 1.64 |
| [zinc finger and btb domain containing 16](http://niaid.abcc.ncifcrf.gov/geneReportFull.jsp?rowids=149025) | 1442025_a_at | ***-2.38*** | -1.33 | 1.80 |
| ***Response to DNA Damage Stimulus (2.1E-2)*** | |  |  |  |
| [b-cell translocation gene 2, anti-proliferative](http://niaid.abcc.ncifcrf.gov/geneReportFull.jsp?rowids=14999) | 1416250_at | ***2.73*** | 1.22 | 1.11 |
| [high mobility group box 2](http://niaid.abcc.ncifcrf.gov/geneReportFull.jsp?rowids=5296) | 1452534_a_at | ***2.02*** | 1.34 | 2.19 |
| [proliferating cell nuclear antigen](http://niaid.abcc.ncifcrf.gov/geneReportFull.jsp?rowids=147183) | 1442433_at | ***-1.79*** | 1.14 | 1.12 |
| [serum/glucocorticoid regulated kinase](http://niaid.abcc.ncifcrf.gov/geneReportFull.jsp?rowids=4122) | 1416041_at | ***-2.28*** | 1.12 | 2.42 |
| ***Nervous System Development (2.0E-1)*** | |  |  |  |
| [fbj osteosarcoma oncogene](http://niaid.abcc.ncifcrf.gov/geneReportFull.jsp?rowids=3873) | 1423100_at | ***2.85*** | 3.57 | 2.42 |
| [b-cell translocation gene 2, anti-proliferative](http://niaid.abcc.ncifcrf.gov/geneReportFull.jsp?rowids=14999) | 1416250_at | ***2.73*** | 1.22 | 1.11 |
| [chemokine (c-x-c motif) receptor 4](http://niaid.abcc.ncifcrf.gov/geneReportFull.jsp?rowids=37712) | 1448710_at | ***2.56*** | 1.78 | 1.64 |
| [zinc finger and btb domain containing 16](http://niaid.abcc.ncifcrf.gov/geneReportFull.jsp?rowids=149025) | 1442025_a_at | ***-2.38*** | -1.33 | 1.80 |
| ***Uncategorized*** | |  |  |  |
| [similar to mhc q8/9d surface antigen](http://niaid.abcc.ncifcrf.gov/geneReportFull.jsp?rowids=136418) | 1418536_at | ***3.62*** | 2.04 | 1.62 |
| [interferon inducible gtpase 1](http://niaid.abcc.ncifcrf.gov/geneReportFull.jsp?rowids=26341) | 1419042_at | ***2.87*** | 2.16 | 1.50 |
| [prostaglandin d2 synthase 2, hematopoietic](http://niaid.abcc.ncifcrf.gov/geneReportFull.jsp?rowids=37483) | 1421492_at | ***2.86*** | 1.46 | 1.34 |
| [t-cell specific gtpase](http://niaid.abcc.ncifcrf.gov/geneReportFull.jsp?rowids=37757) | 1449009_at | ***2.60*** | 1.88 | 1.07 |
| [atpase, h+ transporting, lysosomal v1 subunit b2](http://niaid.abcc.ncifcrf.gov/geneReportFull.jsp?rowids=143784) | 1449649_at | ***2.54*** | -1.68 | 1.65 |
| [ceruloplasmin](http://niaid.abcc.ncifcrf.gov/geneReportFull.jsp?rowids=145690) | 1417497_at | ***2.49*** | 1.23 | 1.36 |
| [riken cdna a330033j07 gene](http://niaid.abcc.ncifcrf.gov/geneReportFull.jsp?rowids=119946) | 1458799_at | ***2.40*** | -1.23 | 1.20 |
| [pleckstrin](http://niaid.abcc.ncifcrf.gov/geneReportFull.jsp?rowids=146193) | 1417523_at | ***2.37*** | 1.50 | 2.41 |
| [cdna sequence bc013672](http://niaid.abcc.ncifcrf.gov/geneReportFull.jsp?rowids=147718) | 1451777_at | ***2.24*** | 1.93 | 2.01 |
| [dual specificity phosphatase 1](http://niaid.abcc.ncifcrf.gov/geneReportFull.jsp?rowids=27750) | 1448830_at | ***2.21*** | 1.58 | 1.43 |
| [poly (adp-ribose) polymerase family, member 9](http://niaid.abcc.ncifcrf.gov/geneReportFull.jsp?rowids=28026) | 1416897_at | ***2.17*** | 2.52 | 1.45 |
| [,gb:bf462648 /db_xref=gi:11531831 /db_xref=ui-m-cg0p-bng-a-09-0-ui.s1 /clone=ui-m-cg0p-bng-a-09-0-ui /fea=est /cnt=4 /tid=mm.59110.1 /tier=consend /stk=3 /ug=mm.59110 /ug_title=ests](http://niaid.abcc.ncifcrf.gov/geneReportFull.jsp?rowids=3166816) | 1442881_at | ***2.15*** | -1.18 | -1.04 |
| [tax1 (human t-cell leukemia virus type i) binding protein 3](http://niaid.abcc.ncifcrf.gov/geneReportFull.jsp?rowids=13314) | 1424169_at | ***1.94*** | -1.10 | 1.07 |
| [solute carrier family 39 (iron-regulated transporter), member 1](http://niaid.abcc.ncifcrf.gov/geneReportFull.jsp?rowids=15116) | 1448566_at | ***1.79*** | -1.11 | -1.51 |
| [cytochrome p450, family 4, subfamily f, polypeptide 14](http://niaid.abcc.ncifcrf.gov/geneReportFull.jsp?rowids=19282) | 1419559_at | ***1.66*** | -1.26 | 1.63 |
| [gtpase, very large interferon inducible 1](http://niaid.abcc.ncifcrf.gov/geneReportFull.jsp?rowids=12510) | 1429184_at | ***1.66*** | 1.53 | -1.27 |
| [riken cdna 5830404h04 gene](http://niaid.abcc.ncifcrf.gov/geneReportFull.jsp?rowids=145094) | 1430544_at | ***1.62*** | -1.03 | -1.42 |
| [expressed sequence c79673](http://niaid.abcc.ncifcrf.gov/geneReportFull.jsp?rowids=27725) | 1433963_a_at | ***1.61*** | 1.23 | 1.96 |
| [riken cdna 2810487a22 gene](http://niaid.abcc.ncifcrf.gov/geneReportFull.jsp?rowids=38847) | 1455292_x_at | ***1.60*** | 1.20 | -1.84 |
| [zinc finger protein 36, c3h type-like 2](http://niaid.abcc.ncifcrf.gov/geneReportFull.jsp?rowids=4200) | 1437626_at | ***1.52*** | 1.35 | 1.42 |
| [expressed sequence c80998](http://niaid.abcc.ncifcrf.gov/geneReportFull.jsp?rowids=124041) | 1456050_at | ***1.51*** | 1.45 | -1.30 |
| [g protein-coupled receptor 17](http://niaid.abcc.ncifcrf.gov/geneReportFull.jsp?rowids=27575) | 1456833_at | ***-1.35*** | 1.17 | -1.19 |
| [riken cdna c920011g20 gene](http://niaid.abcc.ncifcrf.gov/geneReportFull.jsp?rowids=138723) | 1440978_at | ***-1.42*** | -2.41 | 1.14 |
| [riken cdna 4631426h08 gene](http://niaid.abcc.ncifcrf.gov/geneReportFull.jsp?rowids=16129) | 1418173_at | ***-1.53*** | 1.26 | -1.58 |
| [ubiquitin specific peptidase 40](http://niaid.abcc.ncifcrf.gov/geneReportFull.jsp?rowids=33202) | 1438242_at | ***-1.60*** | -1.02 | -1.05 |
| [expressed sequence ai426953](http://niaid.abcc.ncifcrf.gov/geneReportFull.jsp?rowids=99719) | 1442018_at | ***-1.64*** | 1.25 | 2.72 |
| [sh3 domain containing ring finger 1](http://niaid.abcc.ncifcrf.gov/geneReportFull.jsp?rowids=11254) | 1445178_at | ***-1.65*** | 1.16 | -1.11 |
| [,gb:bg068983 /db_xref=gi:12551552 /db_xref=h3071d11-3 /clone=h3071d11 /fea=est /cnt=2 /tid=mm.173721.1 /tier=consend /stk=2 /ug=mm.173721 /ug_title=ests](http://niaid.abcc.ncifcrf.gov/geneReportFull.jsp?rowids=3168455) | 1459176_at | ***-1.86*** | -1.46 | -1.14 |
| [riken cdna 4921519l13 gene](http://niaid.abcc.ncifcrf.gov/geneReportFull.jsp?rowids=23800) | 1425205_at | ***-2.09*** | 1.16 | 2.04 |
| [angiotensin receptor-like 1](http://niaid.abcc.ncifcrf.gov/geneReportFull.jsp?rowids=28297) | 1423037_at | ***-2.09*** | 1.10 | 1.73 |
| [protein tyrosine phosphatase 4a3](http://niaid.abcc.ncifcrf.gov/geneReportFull.jsp?rowids=19401) | 1418182_at | ***-2.18*** | 1.34 | -1.14 |
| [arrestin domain containing 2](http://niaid.abcc.ncifcrf.gov/geneReportFull.jsp?rowids=26328) | 1428352_at | ***-2.20*** | -1.12 | 1.68 |
| [riken cdna 2310039h15 gene](http://niaid.abcc.ncifcrf.gov/geneReportFull.jsp?rowids=117374) | 1458678_at | ***-2.20*** | 1.15 | 1.12 |
| [riken cdna 9630031f12 gene](http://niaid.abcc.ncifcrf.gov/geneReportFull.jsp?rowids=36920) | 1447269_at | ***-2.22*** | -1.08 | 1.05 |
| [riken cdna 4921527h02 gene](http://niaid.abcc.ncifcrf.gov/geneReportFull.jsp?rowids=87641) | 1432695_at | ***-2.26*** | 1.21 | -1.29 |
| [acyl-coa synthetase long-chain family member 1](http://niaid.abcc.ncifcrf.gov/geneReportFull.jsp?rowids=5059) | 1460316_at | ***-2.26*** | 1.11 | 1.04 |
| [elastin microfibril interfacer 2](http://niaid.abcc.ncifcrf.gov/geneReportFull.jsp?rowids=3759) | 1435264_at | ***-2.27*** | -1.02 | 1.49 |
| [riken cdna c330006p03 gene](http://niaid.abcc.ncifcrf.gov/geneReportFull.jsp?rowids=119961) | 1436387_at | ***-2.57*** | 1.01 | 2.11 |
|  |  |  |  |  |
