# Supplementary material for: Gene expression profiling in a mouse model of infantile neuronal ceroid lipofuscinosis reveals upregulation of immediate early genes and mediators of the inflammatory response
Source: BMC Neurosci. 2007 Nov 16;8:95. doi: 10.1186/1471-2202-8-95 (PMC2204004; doi:10.1186/1471-2202-8-95)
Supplement: Additional File 5 — Enriched GO (Gene Ontology) categories (and enrichment scores) for genes regulated at 5 months (Microsoft Word table). For genes represented by more than one probe set, the average of all probe sets was used to calculate the fold change. All genes with expression levels that changed at least two-fold are listed. Genes are first grouped according to Gene Ontology category, and uncategorized genes are also shown. [file 1471-2202-8-95-S5.doc]

| Additional File 5 |  | |  | |  | |  |
| --- | --- | --- | --- | --- | --- | --- | --- |
| **Enriched GO (Gene Ontology) categories (and enrichment scores) for genes regulated at 5 months (expression level up- or down- regulated at least 2 fold). For genes represented by more than one probe set, the average of all probe sets was used to calculate the fold change.** | | | | | | | |
| ***Gene family and gene name*** | ***Affy_ID*** | ***Fold change (KO:WT)*** | | | | | |
|  |  | ***3 month*** | | ***5 month*** | | **8 month** | |
| ***Immune response (6.8E-28)*** | |  | |  | |  | |
| [serine (or cysteine) peptidase inhibitor, clade a, member 3n](http://niaid.abcc.ncifcrf.gov/geneReportFull.jsp?rowids=5270) | 1419100_at | 4.27 | | ***13.83*** | | 20.32 | |
| [c-type lectin domain family 7, member a](http://niaid.abcc.ncifcrf.gov/geneReportFull.jsp?rowids=146079) | 1420699_at | 10.25 | | ***13.58*** | | 14.66 | |
| [leukocyte immunoglobulin-like receptor, subfamily b, member 4](http://niaid.abcc.ncifcrf.gov/geneReportFull.jsp?rowids=8586) | 1420394_s_at | 3.84 | | ***13.43*** | | 16.71 | |
| [complement component 4b (childo blood group)](http://niaid.abcc.ncifcrf.gov/geneReportFull.jsp?rowids=28988) | 1418021_at | 4.79 | | ***10.13*** | | 8.61 | |
| [chemokine (c-c motif) ligand 21b](http://niaid.abcc.ncifcrf.gov/geneReportFull.jsp?rowids=19671) | 1419426_s_at | 11.35 | | ***9.91*** | | 6.85 | |
| [chemokine (c-c motif) ligand 21a](http://niaid.abcc.ncifcrf.gov/geneReportFull.jsp?rowids=56305) | 1419426_s_at | 11.35 | | ***9.91*** | | 6.85 | |
| [chemokine (c-x-c motif) ligand 10](http://niaid.abcc.ncifcrf.gov/geneReportFull.jsp?rowids=3288) | 1418930_at | 7.46 | | ***8.3*** | | 6.69 | |
| [p lysozyme structural](http://niaid.abcc.ncifcrf.gov/geneReportFull.jsp?rowids=20271) | 1436996_x_at, 1439426_x_at | 3.65 | | ***8.17*** | | 14.03 | |
| [chemokine (c-c motif) ligand 12](http://niaid.abcc.ncifcrf.gov/geneReportFull.jsp?rowids=37153) | 1419282_at | 3.36 | | ***7.41*** | | 4.42 | |
| [complement component 3](http://niaid.abcc.ncifcrf.gov/geneReportFull.jsp?rowids=16973) | 1423954_at | 1.18 | | ***6.49*** | | 14.72 | |
| [chemokine (c-x-c motif) ligand 5](http://niaid.abcc.ncifcrf.gov/geneReportFull.jsp?rowids=11501) | 1419728_at | 2.34 | | ***6.09*** | | 2.28 | |
| [macrophage activation 2 like](http://niaid.abcc.ncifcrf.gov/geneReportFull.jsp?rowids=145781) | 1438676_at, 1447927_at | 5.47 | | ***5.94*** | | 3.77 | |
| [lysozyme](http://niaid.abcc.ncifcrf.gov/geneReportFull.jsp?rowids=36909) | 1423547_at | 3.22 | | ***5.79*** | | 11.6 | |
| [interferon-induced protein with tetratricopeptide repeats 1](http://niaid.abcc.ncifcrf.gov/geneReportFull.jsp?rowids=148879) | 1450783_at | 4.91 | | ***5.69*** | | 2.11 | |
| [fc receptor, igg, low affinity iib](http://niaid.abcc.ncifcrf.gov/geneReportFull.jsp?rowids=26631) | 1435477_s_at | 2.65 | | ***4.45*** | | 6.88 | |
| [2'-5' oligoadenylate synthetase-like 2](http://niaid.abcc.ncifcrf.gov/geneReportFull.jsp?rowids=13979) | 1453196_a_at | 5.1 | | ***4*** | | 2.17 | |
| [guanylate nucleotide binding protein 2](http://niaid.abcc.ncifcrf.gov/geneReportFull.jsp?rowids=19413) | 1418240_at, 1435906_x_at | 4.08 | | ***3.93*** | | 2.75 | |
| [endothelial differentiation, sphingolipid g-protein-coupled receptor, 3](http://niaid.abcc.ncifcrf.gov/geneReportFull.jsp?rowids=3654) | 1437173_at, 1438658_a_at | 1.64 | | ***3.89*** | | 4 | |
| [chemokine (c-c motif) ligand 3](http://niaid.abcc.ncifcrf.gov/geneReportFull.jsp?rowids=6055) | 1419561_at | 2.43 | | ***3.84*** | | 2.84 | |
| [integrin alpha x](http://niaid.abcc.ncifcrf.gov/geneReportFull.jsp?rowids=26979) | 1419128_at | 2.31 | | ***3.8*** | | 5.18 | |
| [integrin beta 2](http://niaid.abcc.ncifcrf.gov/geneReportFull.jsp?rowids=23787) | 1450678_at | 1.55 | | ***3.29*** | | 4.63 | |
| [mhc (a.ca/j(h-2k-f) class i antigen](http://niaid.abcc.ncifcrf.gov/geneReportFull.jsp?rowids=146545) | 1424948_x_at, 1451784_x_at | 3.21 | | ***3.09*** | | 1.89 | |
| [lymphocyte antigen 86](http://niaid.abcc.ncifcrf.gov/geneReportFull.jsp?rowids=13741) | 1422903_at | 2.71 | | ***3.05*** | | 2.12 | |
| [histocompatibility 2, k1, k region](http://niaid.abcc.ncifcrf.gov/geneReportFull.jsp?rowids=8197) | 1427746_x_at, 1424948_x_at, 1425336_x_at, 1451784_x_at | 4.12 | | ***2.96*** | | 2.07 | |
| [interferon-induced protein with tetratricopeptide repeats 3](http://niaid.abcc.ncifcrf.gov/geneReportFull.jsp?rowids=134904) | 1449025_at | 3.15 | | ***2.84*** | | 1.75 | |
| [chemokine (c-c motif) ligand 5](http://niaid.abcc.ncifcrf.gov/geneReportFull.jsp?rowids=25442) | 1418126_at | 2.63 | | ***2.77*** | | 3.76 | |
| [protein tyrosine phosphatase, receptor type, c](http://niaid.abcc.ncifcrf.gov/geneReportFull.jsp?rowids=10409) | 1422124_a_at | 2.8 | | ***2.75*** | | 2.12 | |
| [histocompatibility 2, d region locus 1](http://niaid.abcc.ncifcrf.gov/geneReportFull.jsp?rowids=13267) | 1424948_x_at, 1452548_x_at, 1451931_x_at, 1451784_x_at, 1451683_x_at, 1425545_x_at | 2.64 | | ***2.68*** | | 2.1 | |
| [fc receptor, ige, high affinity i, gamma polypeptide](http://niaid.abcc.ncifcrf.gov/geneReportFull.jsp?rowids=7045) | 1418340_at | 2.24 | | ***2.65*** | | 3.34 | |
| [complement component 3a receptor 1](http://niaid.abcc.ncifcrf.gov/geneReportFull.jsp?rowids=4503) | 1442082_at | 2.12 | | ***2.62*** | | 3.26 | |
| [dead (asp-glu-ala-asp) box polypeptide 58](http://niaid.abcc.ncifcrf.gov/geneReportFull.jsp?rowids=12911) | 1456890_at | 1.53 | | ***2.58*** | | 1.25 | |
| [complement component 1, q subcomponent, alpha polypeptide](http://niaid.abcc.ncifcrf.gov/geneReportFull.jsp?rowids=35852) | 1417381_at | 2.32 | | ***2.57*** | | 3.2 | |
| [complement component 1, q subcomponent, c chain](http://niaid.abcc.ncifcrf.gov/geneReportFull.jsp?rowids=6708) | 1449401_at | 2.17 | | ***2.55*** | | 3.28 | |
| [histocompatibility 2, class ii antigen a, alpha](http://niaid.abcc.ncifcrf.gov/geneReportFull.jsp?rowids=20507) | 1435290_x_at | 2.42 | | ***2.51*** | | 2.11 | |
| [chemokine (c-x-c motif) ligand 1](http://niaid.abcc.ncifcrf.gov/geneReportFull.jsp?rowids=146753) | 1419209_at | 1.75 | | ***2.47*** | | 2.88 | |
| [guanylate nucleotide binding protein 4](http://niaid.abcc.ncifcrf.gov/geneReportFull.jsp?rowids=146307) | 1418392_a_at | 2.22 | | ***2.46*** | | 2.92 | |
| [chemokine (c-c motif) ligand 6](http://niaid.abcc.ncifcrf.gov/geneReportFull.jsp?rowids=16920) | 1417266_at, 1420249_s_at | 2.03 | | ***2.4*** | | 2.01 | |
| [beta-2 microglobulin](http://niaid.abcc.ncifcrf.gov/geneReportFull.jsp?rowids=10256) | 1449289_a_at, 1452428_a_at | 2.28 | | ***2.36*** | | 2.22 | |
| [proteosome (prosome, macropain) subunit, beta type 8 (large multifunctional peptidase 7)](http://niaid.abcc.ncifcrf.gov/geneReportFull.jsp?rowids=34173) | 1422962_a_at | 1.76 | | ***2.35*** | | 1.77 | |
| [riken cdna 0610037m15 gene](http://niaid.abcc.ncifcrf.gov/geneReportFull.jsp?rowids=33619) | 1431008_at | 2.52 | | ***2.32*** | | 2.31 | |
| [complement component 1, q subcomponent, beta polypeptide](http://niaid.abcc.ncifcrf.gov/geneReportFull.jsp?rowids=13238) | 1417063_at, 1437726_x_at | 2.02 | | ***2.3*** | | 2.93 | |
| [chemokine (c-c motif) ligand 9](http://niaid.abcc.ncifcrf.gov/geneReportFull.jsp?rowids=37134) | 1417936_at | 1.59 | | ***2.27*** | | 1.12 | |
| [fyn binding protein](http://niaid.abcc.ncifcrf.gov/geneReportFull.jsp?rowids=30200) | 1435144_at | 1.69 | | ***2.25*** | | 3.69 | |
| [riken cdna 9830147j24 gene](http://niaid.abcc.ncifcrf.gov/geneReportFull.jsp?rowids=5889) | 1434380_at | 2.66 | | ***2.23*** | | 1.34 | |
| [integrin alpha m](http://niaid.abcc.ncifcrf.gov/geneReportFull.jsp?rowids=145076) | 1422046_at | 1.46 | | ***2.18*** | | 1.04 | |
| [interferon gamma inducible protein 30](http://niaid.abcc.ncifcrf.gov/geneReportFull.jsp?rowids=145219) | 1422476_at | 2.16 | | ***2.03*** | | 2.39 | |
| [mhc class i antigen](http://niaid.abcc.ncifcrf.gov/geneReportFull.jsp?rowids=37674) | 1452548_x_at | -1.48 | | ***-2.14*** | | -1.06 | |
| [similar to histocompatibility 2, t region locus 3](http://niaid.abcc.ncifcrf.gov/geneReportFull.jsp?rowids=9640) | 1452548_x_at | -1.48 | | ***-2.14*** | | -1.06 | |
| [histocompatibility 2, t region locus 3](http://niaid.abcc.ncifcrf.gov/geneReportFull.jsp?rowids=145074) | 1452548_x_at | -1.48 | | ***-2.14*** | | -1.06 | |
| ***Response to pest, pathogen or parasite (3.3E-14)*** | |  | |  | |  | |
| [serine (or cysteine) peptidase inhibitor, clade a, member 3n](http://niaid.abcc.ncifcrf.gov/geneReportFull.jsp?rowids=5270) | 1419100_at | 4.27 | | ***13.83*** | | 20.32 | |
| [complement component 4b (childo blood group)](http://niaid.abcc.ncifcrf.gov/geneReportFull.jsp?rowids=28988) | 1418021_at | 4.79 | | ***10.13*** | | 8.61 | |
| [chemokine (c-c motif) ligand 21b](http://niaid.abcc.ncifcrf.gov/geneReportFull.jsp?rowids=19671) | 1419426_s_at | 11.35 | | ***9.91*** | | 6.85 | |
| [chemokine (c-c motif) ligand 21a](http://niaid.abcc.ncifcrf.gov/geneReportFull.jsp?rowids=56305) | 1419426_s_at | 11.35 | | ***9.91*** | | 6.85 | |
| [chemokine (c-x-c motif) ligand 10](http://niaid.abcc.ncifcrf.gov/geneReportFull.jsp?rowids=3288) | 1418930_at | 7.46 | | ***8.3*** | | 6.69 | |
| [p lysozyme structural](http://niaid.abcc.ncifcrf.gov/geneReportFull.jsp?rowids=20271) | 1436996_x_at, 1439426_x_at | 3.65 | | ***8.17*** | | 14.03 | |
| [chemokine (c-c motif) ligand 12](http://niaid.abcc.ncifcrf.gov/geneReportFull.jsp?rowids=37153) | 1419282_at | 3.36 | | ***7.41*** | | 4.42 | |
| [complement component 3](http://niaid.abcc.ncifcrf.gov/geneReportFull.jsp?rowids=16973) | 1423954_at | 1.18 | | ***6.49*** | | 14.72 | |
| [chemokine (c-x-c motif) ligand 5](http://niaid.abcc.ncifcrf.gov/geneReportFull.jsp?rowids=11501) | 1419728_at | 2.34 | | ***6.09*** | | 2.28 | |
| [lysozyme](http://niaid.abcc.ncifcrf.gov/geneReportFull.jsp?rowids=36909) | 1423547_at | 3.22 | | ***5.79*** | | 11.6 | |
| [fc receptor, igg, low affinity iib](http://niaid.abcc.ncifcrf.gov/geneReportFull.jsp?rowids=26631) | 1435477_s_at | 2.65 | | ***4.45*** | | 6.88 | |
| [endothelial differentiation, sphingolipid g-protein-coupled receptor, 3](http://niaid.abcc.ncifcrf.gov/geneReportFull.jsp?rowids=3654) | 1437173_at, 1438658_a_at | 1.64 | | ***3.89*** | | 4 | |
| [chemokine (c-c motif) ligand 3](http://niaid.abcc.ncifcrf.gov/geneReportFull.jsp?rowids=6055) | 1419561_at | 2.43 | | ***3.84*** | | 2.84 | |
| [interferon, alpha-inducible protein 27](http://niaid.abcc.ncifcrf.gov/geneReportFull.jsp?rowids=38144) | 1426278_at | 10.18 | | ***3.37*** | | 1.81 | |
| [integrin beta 2](http://niaid.abcc.ncifcrf.gov/geneReportFull.jsp?rowids=23787) | 1450678_at | 1.55 | | ***3.29*** | | 4.63 | |
| [lymphocyte antigen 86](http://niaid.abcc.ncifcrf.gov/geneReportFull.jsp?rowids=13741) | 1422903_at | 2.71 | | ***3.05*** | | 2.12 | |
| [chemokine (c-c motif) ligand 5](http://niaid.abcc.ncifcrf.gov/geneReportFull.jsp?rowids=25442) | 1418126_at | 2.63 | | ***2.77*** | | 3.76 | |
| [protein tyrosine phosphatase, receptor type, c](http://niaid.abcc.ncifcrf.gov/geneReportFull.jsp?rowids=10409) | 1422124_a_at | 2.8 | | ***2.75*** | | 2.12 | |
| [fc receptor, ige, high affinity i, gamma polypeptide](http://niaid.abcc.ncifcrf.gov/geneReportFull.jsp?rowids=7045) | 1418340_at | 2.24 | | ***2.65*** | | 3.34 | |
| [complement component 3a receptor 1](http://niaid.abcc.ncifcrf.gov/geneReportFull.jsp?rowids=4503) | 1442082_at | 2.12 | | ***2.62*** | | 3.26 | |
| [dead (asp-glu-ala-asp) box polypeptide 58](http://niaid.abcc.ncifcrf.gov/geneReportFull.jsp?rowids=12911) | 1456890_at | 1.53 | | ***2.58*** | | 1.25 | |
| [complement component 1, q subcomponent, alpha polypeptide](http://niaid.abcc.ncifcrf.gov/geneReportFull.jsp?rowids=35852) | 1417381_at | 2.32 | | ***2.57*** | | 3.2 | |
| [complement component 1, q subcomponent, c chain](http://niaid.abcc.ncifcrf.gov/geneReportFull.jsp?rowids=6708) | 1449401_at | 2.17 | | ***2.55*** | | 3.28 | |
| [chemokine (c-x-c motif) ligand 1](http://niaid.abcc.ncifcrf.gov/geneReportFull.jsp?rowids=146753) | 1419209_at | 1.75 | | ***2.47*** | | 2.88 | |
| [beta-2 microglobulin](http://niaid.abcc.ncifcrf.gov/geneReportFull.jsp?rowids=10256) | 1449289_a_at, 1452428_a_at | 2.28 | | ***2.36*** | | 2.22 | |
| [complement component 1, q subcomponent, beta polypeptide](http://niaid.abcc.ncifcrf.gov/geneReportFull.jsp?rowids=13238) | 1417063_at, 1437726_x_at | 2.02 | | ***2.3*** | | 2.93 | |
| [integrin alpha m](http://niaid.abcc.ncifcrf.gov/geneReportFull.jsp?rowids=145076) | 1422046_at | 1.46 | | ***2.18*** | | 1.04 | |
| [dna segment, chr 12, erato doi 647, expressed](http://niaid.abcc.ncifcrf.gov/geneReportFull.jsp?rowids=16205) | 1454757_s_at, 1452956_a_at | 2.37 | | ***2.06*** | | 1.51 | |
| ***Inflammatory repsonse (5.8E-10)*** | |  | |  | |  | |
| [complement component 4b (childo blood group)](http://niaid.abcc.ncifcrf.gov/geneReportFull.jsp?rowids=28988) | 1418021_at | 4.79 | | ***10.13*** | | 8.61 | |
| [chemokine (c-c motif) ligand 21b](http://niaid.abcc.ncifcrf.gov/geneReportFull.jsp?rowids=19671) | 1419426_s_at | 11.35 | | ***9.91*** | | 6.85 | |
| [chemokine (c-c motif) ligand 21a](http://niaid.abcc.ncifcrf.gov/geneReportFull.jsp?rowids=56305) | 1419426_s_at | 11.35 | | ***9.91*** | | 6.85 | |
| [chemokine (c-x-c motif) ligand 10](http://niaid.abcc.ncifcrf.gov/geneReportFull.jsp?rowids=3288) | 1418930_at | 7.46 | | ***8.3*** | | 6.69 | |
| [chemokine (c-c motif) ligand 12](http://niaid.abcc.ncifcrf.gov/geneReportFull.jsp?rowids=37153) | 1419282_at | 3.36 | | ***7.41*** | | 4.42 | |
| [complement component 3](http://niaid.abcc.ncifcrf.gov/geneReportFull.jsp?rowids=16973) | 1423954_at | 1.18 | | ***6.49*** | | 14.72 | |
| [chemokine (c-x-c motif) ligand 5](http://niaid.abcc.ncifcrf.gov/geneReportFull.jsp?rowids=11501) | 1419728_at | 2.34 | | ***6.09*** | | 2.28 | |
| [fc receptor, igg, low affinity iib](http://niaid.abcc.ncifcrf.gov/geneReportFull.jsp?rowids=26631) | 1435477_s_at | 2.65 | | ***4.45*** | | 6.88 | |
| [endothelial differentiation, sphingolipid g-protein-coupled receptor, 3](http://niaid.abcc.ncifcrf.gov/geneReportFull.jsp?rowids=3654) | 1437173_at, 1438658_a_at | 1.64 | | ***3.89*** | | 4 | |
| [chemokine (c-c motif) ligand 3](http://niaid.abcc.ncifcrf.gov/geneReportFull.jsp?rowids=6055) | 1419561_at | 2.43 | | ***3.84*** | | 2.84 | |
| [integrin beta 2](http://niaid.abcc.ncifcrf.gov/geneReportFull.jsp?rowids=23787) | 1450678_at | 1.55 | | ***3.29*** | | 4.63 | |
| [lymphocyte antigen 86](http://niaid.abcc.ncifcrf.gov/geneReportFull.jsp?rowids=13741) | 1422903_at | 2.71 | | ***3.05*** | | 2.12 | |
| [chemokine (c-c motif) ligand 5](http://niaid.abcc.ncifcrf.gov/geneReportFull.jsp?rowids=25442) | 1418126_at | 2.63 | | ***2.77*** | | 3.76 | |
| [fc receptor, ige, high affinity i, gamma polypeptide](http://niaid.abcc.ncifcrf.gov/geneReportFull.jsp?rowids=7045) | 1418340_at | 2.24 | | ***2.65*** | | 3.34 | |
| [chemokine (c-x-c motif) ligand 1](http://niaid.abcc.ncifcrf.gov/geneReportFull.jsp?rowids=146753) | 1419209_at | 1.75 | | ***2.47*** | | 2.88 | |
| [integrin alpha m](http://niaid.abcc.ncifcrf.gov/geneReportFull.jsp?rowids=145076) | 1422046_at | 1.46 | | ***2.18*** | | 1.04 | |
| ***Antigen Presentation (1.3E-10)*** | |  | |  | |  | |
| [fc receptor, igg, low affinity iib](http://niaid.abcc.ncifcrf.gov/geneReportFull.jsp?rowids=26631) | 1435477_s_at | 2.65 | | ***4.45*** | | 6.88 | |
| [mhc (a.ca/j(h-2k-f) class i antigen](http://niaid.abcc.ncifcrf.gov/geneReportFull.jsp?rowids=146545) | 1424948_x_at, 1451784_x_at | 3.21 | | ***3.09*** | | 1.89 | |
| [histocompatibility 2, k1, k region](http://niaid.abcc.ncifcrf.gov/geneReportFull.jsp?rowids=8197) | 1427746_x_at, 1424948_x_at, 1425336_x_at, 1451784_x_at | 4.12 | | ***2.96*** | | 2.07 | |
| [histocompatibility 2, d region locus 1](http://niaid.abcc.ncifcrf.gov/geneReportFull.jsp?rowids=13267) | 1424948_x_at, 1452548_x_at, 1451931_x_at, 1451784_x_at, 1451683_x_at, 1425545_x_at | 2.64 | | ***2.68*** | | 2.1 | |
| [fc receptor, ige, high affinity i, gamma polypeptide](http://niaid.abcc.ncifcrf.gov/geneReportFull.jsp?rowids=7045) | 1418340_at | 2.24 | | ***2.65*** | | 3.34 | |
| [histocompatibility 2, class ii antigen a, alpha](http://niaid.abcc.ncifcrf.gov/geneReportFull.jsp?rowids=20507) | 1435290_x_at | 2.42 | | ***2.51*** | | 2.11 | |
| [beta-2 microglobulin](http://niaid.abcc.ncifcrf.gov/geneReportFull.jsp?rowids=10256) | 1449289_a_at, 1452428_a_at | 2.28 | | ***2.36*** | | 2.22 | |
| [proteosome (prosome, macropain) subunit, beta type 8 (large multifunctional peptidase 7)](http://niaid.abcc.ncifcrf.gov/geneReportFull.jsp?rowids=34173) | 1422962_a_at | 1.76 | | ***2.35*** | | 1.77 | |
| [riken cdna 0610037m15 gene](http://niaid.abcc.ncifcrf.gov/geneReportFull.jsp?rowids=33619) | 1431008_at | 2.52 | | ***2.32*** | | 2.31 | |
| [similar to histocompatibility 2, t region locus 3](http://niaid.abcc.ncifcrf.gov/geneReportFull.jsp?rowids=9640) | 1452548_x_at | -1.48 | | ***-2.14*** | | -1.06 | |
| [mhc class i antigen](http://niaid.abcc.ncifcrf.gov/geneReportFull.jsp?rowids=37674) | 1452548_x_at | -1.48 | | ***-2.14*** | | -1.06 | |
| [histocompatibility 2, t region locus 3](http://niaid.abcc.ncifcrf.gov/geneReportFull.jsp?rowids=145074) | 1452548_x_at | -1.48 | | ***-2.14*** | | -1.06 | |
| ***Chemotaxis (2.6E-9)*** | |  | |  | |  | |
| [chemokine (c-c motif) ligand 21b](http://niaid.abcc.ncifcrf.gov/geneReportFull.jsp?rowids=19671) | 1419426_s_at | 11.35 | | ***9.91*** | | 6.85 | |
| [chemokine (c-c motif) ligand 21a](http://niaid.abcc.ncifcrf.gov/geneReportFull.jsp?rowids=56305) | 1419426_s_at | 11.35 | | ***9.91*** | | 6.85 | |
| [chemokine (c-x-c motif) ligand 10](http://niaid.abcc.ncifcrf.gov/geneReportFull.jsp?rowids=3288) | 1418930_at | 7.46 | | ***8.3*** | | 6.69 | |
| [chemokine (c-c motif) ligand 12](http://niaid.abcc.ncifcrf.gov/geneReportFull.jsp?rowids=37153) | 1419282_at | 3.36 | | ***7.41*** | | 4.42 | |
| [chemokine (c-x-c motif) ligand 5](http://niaid.abcc.ncifcrf.gov/geneReportFull.jsp?rowids=11501) | 1419728_at | 2.34 | | ***6.09*** | | 2.28 | |
| [chemokine (c-c motif) ligand 3](http://niaid.abcc.ncifcrf.gov/geneReportFull.jsp?rowids=6055) | 1419561_at | 2.43 | | ***3.84*** | | 2.84 | |
| [integrin beta 2](http://niaid.abcc.ncifcrf.gov/geneReportFull.jsp?rowids=23787) | 1450678_at | 1.55 | | ***3.29*** | | 4.63 | |
| [chemokine (c-c motif) ligand 5](http://niaid.abcc.ncifcrf.gov/geneReportFull.jsp?rowids=25442) | 1418126_at | 2.63 | | ***2.77*** | | 3.76 | |
| [fc receptor, ige, high affinity i, gamma polypeptide](http://niaid.abcc.ncifcrf.gov/geneReportFull.jsp?rowids=7045) | 1418340_at | 2.24 | | ***2.65*** | | 3.34 | |
| [complement component 3a receptor 1](http://niaid.abcc.ncifcrf.gov/geneReportFull.jsp?rowids=4503) | 1442082_at | 2.12 | | ***2.62*** | | 3.26 | |
| [chemokine (c-c motif) ligand 6](http://niaid.abcc.ncifcrf.gov/geneReportFull.jsp?rowids=16920) | 1417266_at, 1420249_s_at | 2.03 | | ***2.4*** | | 2.01 | |
| [chemokine (c-c motif) ligand 9](http://niaid.abcc.ncifcrf.gov/geneReportFull.jsp?rowids=37134) | 1417936_at | 1.59 | | ***2.27*** | | 1.12 | |
| [integrin alpha m](http://niaid.abcc.ncifcrf.gov/geneReportFull.jsp?rowids=145076) | 1422046_at | 1.46 | | ***2.18*** | | 1.04 | |
| ***Positive Regulation of Phagocytosis (6.4E-5)*** | |  | |  | |  | |
| [c-type lectin domain family 7, member a](http://niaid.abcc.ncifcrf.gov/geneReportFull.jsp?rowids=146079) | 1420699_at | 10.25 | | ***13.58*** | | 14.66 | |
| [complement component 3](http://niaid.abcc.ncifcrf.gov/geneReportFull.jsp?rowids=16973) | 1423954_at | 1.18 | | ***6.49*** | | 14.72 | |
| [fc receptor, igg, low affinity iib](http://niaid.abcc.ncifcrf.gov/geneReportFull.jsp?rowids=26631) | 1435477_s_at | 2.65 | | ***4.45*** | | 6.88 | |
| [pentraxin related gene](http://niaid.abcc.ncifcrf.gov/geneReportFull.jsp?rowids=144233) | 1418666_at | 2 | | ***2.72*** | | 3.53 | |
| [fc receptor, ige, high affinity i, gamma polypeptide](http://niaid.abcc.ncifcrf.gov/geneReportFull.jsp?rowids=7045) | 1418340_at | 2.24 | | ***2.65*** | | 3.34 | |
| ***Complement Activation (9.1E-5)*** | |  | |  | |  | |
| [complement component 4b (childo blood group)](http://niaid.abcc.ncifcrf.gov/geneReportFull.jsp?rowids=28988) | 1418021_at | 4.79 | | ***10.13*** | | 8.61 | |
| [complement component 3](http://niaid.abcc.ncifcrf.gov/geneReportFull.jsp?rowids=16973) | 1423954_at | 1.18 | | ***6.49*** | | 14.72 | |
| [complement component 3a receptor 1](http://niaid.abcc.ncifcrf.gov/geneReportFull.jsp?rowids=4503) | 1442082_at | 2.12 | | ***2.62*** | | 3.26 | |
| [complement component 1, q subcomponent, alpha polypeptide](http://niaid.abcc.ncifcrf.gov/geneReportFull.jsp?rowids=35852) | 1417381_at | 2.32 | | ***2.57*** | | 3.2 | |
| [complement component 1, q subcomponent, c chain](http://niaid.abcc.ncifcrf.gov/geneReportFull.jsp?rowids=6708) | 1449401_at | 2.17 | | ***2.55*** | | 3.28 | |
| [complement component 1, q subcomponent, beta polypeptide](http://niaid.abcc.ncifcrf.gov/geneReportFull.jsp?rowids=13238) | 1417063_at, 1437726_x_at | 2.02 | | ***2.3*** | | 2.93 | |
| ***Immune cell migration (3.1E-3)*** | |  | |  | |  | |
| [chemokine (c-c motif) ligand 21a](http://niaid.abcc.ncifcrf.gov/geneReportFull.jsp?rowids=56305) | 1419426_s_at | 11.35 | | ***9.91*** | | 6.85 | |
| [integrin beta 2](http://niaid.abcc.ncifcrf.gov/geneReportFull.jsp?rowids=23787) | 1450678_at | 1.55 | | ***3.29*** | | 4.63 | |
| [fc receptor, ige, high affinity i, gamma polypeptide](http://niaid.abcc.ncifcrf.gov/geneReportFull.jsp?rowids=7045) | 1418340_at | 2.24 | | ***2.65*** | | 3.34 | |
| [integrin alpha m](http://niaid.abcc.ncifcrf.gov/geneReportFull.jsp?rowids=145076) | 1422046_at | 1.46 | | ***2.18*** | | 1.04 | |
| ***Cell Adhesion Molecules (3.3E-4)*** | |  | |  | |  | |
| [integrin beta 2](http://niaid.abcc.ncifcrf.gov/geneReportFull.jsp?rowids=23787) | 1450678_at | 1.55 | | ***3.29*** | | 4.63 | |
| [mhc (a.ca/j(h-2k-f) class i antigen](http://niaid.abcc.ncifcrf.gov/geneReportFull.jsp?rowids=146545) | 1424948_x_at, 1451784_x_at | 3.21 | | ***3.09*** | | 1.89 | |
| [histocompatibility 2, k1, k region](http://niaid.abcc.ncifcrf.gov/geneReportFull.jsp?rowids=8197) | 1427746_x_at, 1424948_x_at, 1425336_x_at, 1451784_x_at | 4.12 | | ***2.96*** | | 2.07 | |
| [cd86 antigen](http://niaid.abcc.ncifcrf.gov/geneReportFull.jsp?rowids=32785) | 1420404_at | 1.38 | | ***2.87*** | | 1.54 | |
| [protein tyrosine phosphatase, receptor type, c](http://niaid.abcc.ncifcrf.gov/geneReportFull.jsp?rowids=10409) | 1422124_a_at | 2.8 | | ***2.75*** | | 2.12 | |
| [cd22 antigen](http://niaid.abcc.ncifcrf.gov/geneReportFull.jsp?rowids=17050) | 1419768_at | 1.15 | | ***2.7*** | | 2.4 | |
| [histocompatibility 2, d region locus 1](http://niaid.abcc.ncifcrf.gov/geneReportFull.jsp?rowids=13267) | 1424948_x_at, 1452548_x_at, 1451931_x_at, 1451784_x_at, 1451683_x_at, 1425545_x_at | 2.64 | | ***2.68*** | | 2.1 | |
| [histocompatibility 2, class ii antigen a, alpha](http://niaid.abcc.ncifcrf.gov/geneReportFull.jsp?rowids=20507) | 1435290_x_at | 2.42 | | ***2.51*** | | 2.11 | |
| [riken cdna 0610037m15 gene](http://niaid.abcc.ncifcrf.gov/geneReportFull.jsp?rowids=33619) |  | 2.52 | | ***2.32*** | | 2.31 | |
| [integrin alpha m](http://niaid.abcc.ncifcrf.gov/geneReportFull.jsp?rowids=145076) | 1422046_at | 1.46 | | ***2.18*** | | 1.04 | |
| [histocompatibility 2, t region locus 3](http://niaid.abcc.ncifcrf.gov/geneReportFull.jsp?rowids=145074) | 1452548_x_at | -1.48 | | ***-2.14*** | | -1.06 | |
| ***Immune Cell activation (1.7E-2)*** | |  | |  | |  | |
| [fc receptor, igg, low affinity iib](http://niaid.abcc.ncifcrf.gov/geneReportFull.jsp?rowids=26631) | 1435477_s_at | 2.65 | | ***4.45*** | | 6.88 | |
| [integrin alpha x](http://niaid.abcc.ncifcrf.gov/geneReportFull.jsp?rowids=26979) | 1419128_at | 2.31 | | ***3.8*** | | 5.18 | |
| [integrin beta 2](http://niaid.abcc.ncifcrf.gov/geneReportFull.jsp?rowids=23787) | 1450678_at | 1.55 | | ***3.29*** | | 4.63 | |
| [protein tyrosine phosphatase, receptor type, c](http://niaid.abcc.ncifcrf.gov/geneReportFull.jsp?rowids=10409) | 1422124_a_at | 2.8 | | ***2.75*** | | 2.12 | |
| [histocompatibility 2, class ii antigen a, alpha](http://niaid.abcc.ncifcrf.gov/geneReportFull.jsp?rowids=20507) | 1435290_x_at | 2.42 | | ***2.51*** | | 2.11 | |
| [fyn binding protein](http://niaid.abcc.ncifcrf.gov/geneReportFull.jsp?rowids=30200) | 1435144_at | 1.69 | | ***2.25*** | | 3.69 | |
| [integrin alpha m](http://niaid.abcc.ncifcrf.gov/geneReportFull.jsp?rowids=145076) | 1422046_at | 1.46 | | ***2.18*** | | 1.04 | |
| ***Positive regulation of immune response (1.7E-2)*** | |  | |  | |  | |
| [c-type lectin domain family 7, member a](http://niaid.abcc.ncifcrf.gov/geneReportFull.jsp?rowids=146079) | 1420699_at | 10.25 | | ***13.58*** | | 14.66 | |
| [complement component 3](http://niaid.abcc.ncifcrf.gov/geneReportFull.jsp?rowids=16973) | 1423954_at | 1.18 | | ***6.49*** | | 14.72 | |
| [protein tyrosine phosphatase, receptor type, c](http://niaid.abcc.ncifcrf.gov/geneReportFull.jsp?rowids=10409) | 1422124_a_at | 2.8 | | ***2.75*** | | 2.12 | |
| [fc receptor, ige, high affinity i, gamma polypeptide](http://niaid.abcc.ncifcrf.gov/geneReportFull.jsp?rowids=7045) | 1418340_at | 2.24 | | ***2.65*** | | 3.34 | |
| [histocompatibility 2, class ii antigen a, alpha](http://niaid.abcc.ncifcrf.gov/geneReportFull.jsp?rowids=20507) | 1435290_x_at | 2.42 | | ***2.51*** | | 2.11 | |
| ***Lysosome (3.4E-3)*** | |  | |  | |  | |
| [cd68 antigen](http://niaid.abcc.ncifcrf.gov/geneReportFull.jsp?rowids=146071) | 1449164_at | 2.42 | | ***5.16*** | | 4.75 | |
| [histocompatibility 2, class ii antigen a, alpha](http://niaid.abcc.ncifcrf.gov/geneReportFull.jsp?rowids=20507) | 1435290_x_at | 2.42 | | ***2.51*** | | 2.11 | |
| [cathepsin s](http://niaid.abcc.ncifcrf.gov/geneReportFull.jsp?rowids=145797) | 1448591_at | 2.09 | | ***2.4*** | | 2.67 | |
| [cathepsin z](http://niaid.abcc.ncifcrf.gov/geneReportFull.jsp?rowids=145545) | 1417868_a_at, 1417870_x_at | 1.77 | | ***2.22*** | | 2.24 | |
| [cathepsin d](http://niaid.abcc.ncifcrf.gov/geneReportFull.jsp?rowids=15261) | 1448118_a_at | 1.54 | | ***2.12*** | | 2.66 | |
| [interferon gamma inducible protein 30](http://niaid.abcc.ncifcrf.gov/geneReportFull.jsp?rowids=145219) | 1422476_at | 2.16 | | ***2.03*** | | 2.39 | |
| [palmitoyl-protein thioesterase 1](http://niaid.abcc.ncifcrf.gov/geneReportFull.jsp?rowids=147223) | 1422467_at, 1422468_at | -41.25 | | ***-31.17*** | | -24.59 | |
| ***Protease Inhibitor Activity (7.0E-4)*** | |  | |  | |  | |
| serine (or cysteine) peptidase inhibitor, clade a, member 3n | 1419100_at | 4.27 | | ***13.83*** | | 20.32 | |
| [cystatin f (leukocystatin)](http://niaid.abcc.ncifcrf.gov/geneReportFull.jsp?rowids=4102) | 1419202_at | 5.28 | | ***12.02*** | | 13.58 | |
| complement component 4b (childo blood group) | 1418021_at | 4.79 | | ***10.13*** | | 8.61 | |
| complement component 3 | 1423954_at | 1.18 | | ***6.49*** | | 14.72 | |
| alpha-2-macroglobulin | 1434719_at | 1.34 | | ***3.08*** | | 4.99 | |
| [tissue inhibitor of metalloproteinase 1](http://niaid.abcc.ncifcrf.gov/geneReportFull.jsp?rowids=12921) | 1460227_at | 1.45 | | ***2.88*** | | 6.92 | |
| procollagen lysine, 2-oxoglutarate 5-dioxygenase 2 | 1416686_at | 1.61 | | ***2.57*** | | 1.2 | |
| serine (or cysteine) peptidase inhibitor, clade b, member 1a | 1416318_at | 2.41 | | ***2.1*** | | -1.62 | |
| ***Water Transport (8.5E-3)*** | |  | |  | |  | |
| [podoplanin](http://niaid.abcc.ncifcrf.gov/geneReportFull.jsp?rowids=27708) | 1419309_at | 1.63 | | ***2.75*** | | 2.77 | |
| [aquaporin 4](http://niaid.abcc.ncifcrf.gov/geneReportFull.jsp?rowids=35420) | 1425382_a_at, 1447745_at, 1434449_at | 1.57 | | ***2.25*** | | 2.31 | |
| [riken cdna 2610507k20 gene](http://niaid.abcc.ncifcrf.gov/geneReportFull.jsp?rowids=3884) | 1428114_at, 1448640_at | 1.81 | | ***2.21*** | | 2.39 | |
| ***GTPase Activity (1.1E-2)*** | |  | |  | |  | |
| [macrophage activation 2 like](http://niaid.abcc.ncifcrf.gov/geneReportFull.jsp?rowids=145781) | 1438676_at, 1447927_at | 5.47 | | ***5.94*** | | 3.77 | |
| [riken cdna 1110005o19 gene](http://niaid.abcc.ncifcrf.gov/geneReportFull.jsp?rowids=10877) | 1418892_at | 5.62 | | ***5.54*** | | 13.35 | |
| [guanylate nucleotide binding protein 2](http://niaid.abcc.ncifcrf.gov/geneReportFull.jsp?rowids=19413) | 1418240_at, 1435906_x_at | 4.08 | | ***3.93*** | | 2.75 | |
| [guanylate nucleotide binding protein 4](http://niaid.abcc.ncifcrf.gov/geneReportFull.jsp?rowids=146307) | 1418392_a_at | 2.22 | | ***2.46*** | | 2.92 | |
| [interferon inducible gtpase 1](http://niaid.abcc.ncifcrf.gov/geneReportFull.jsp?rowids=26341) | 1419043_a_at | 2.67 | | ***2.29*** | | 1.51 | |
| [riken cdna 9830147j24 gene](http://niaid.abcc.ncifcrf.gov/geneReportFull.jsp?rowids=5889) | 1434380_at | 2.66 | | ***2.23*** | | 1.34 | |
| ***Cell proliferation (1.6E-2)*** | |  | |  | |  | |
| [fc receptor, igg, low affinity iib](http://niaid.abcc.ncifcrf.gov/geneReportFull.jsp?rowids=26631) | 1435477_s_at | 2.65 | | ***4.45*** | | 6.88 | |
| [endothelial differentiation, sphingolipid g-protein-coupled receptor, 3](http://niaid.abcc.ncifcrf.gov/geneReportFull.jsp?rowids=3654) | 1437173_at, 1438658_a_at | 1.64 | | ***3.89*** | | 4 | |
| [integrin alpha x](http://niaid.abcc.ncifcrf.gov/geneReportFull.jsp?rowids=26979) | 1419128_at | 2.31 | | ***3.8*** | | 5.18 | |
| [s100 calcium binding protein a6 (calcyclin)](http://niaid.abcc.ncifcrf.gov/geneReportFull.jsp?rowids=3272) | 1421375_a_at | 1.8 | | ***3.45*** | | 4.64 | |
| [integrin beta 2](http://niaid.abcc.ncifcrf.gov/geneReportFull.jsp?rowids=23787) | 1450678_at | 1.55 | | ***3.29*** | | 4.63 | |
| [protein tyrosine phosphatase, receptor type, c](http://niaid.abcc.ncifcrf.gov/geneReportFull.jsp?rowids=10409) | 1422124_a_at | 2.8 | | ***2.75*** | | 2.12 | |
| [interferon induced transmembrane protein 3](http://niaid.abcc.ncifcrf.gov/geneReportFull.jsp?rowids=148909) | 1423754_at | 2.57 | | ***2.72*** | | 2.75 | |
| [integrin alpha m](http://niaid.abcc.ncifcrf.gov/geneReportFull.jsp?rowids=145076) | 1422046_at | 1.46 | | ***2.18*** | | 1.04 | |
| [granulin](http://niaid.abcc.ncifcrf.gov/geneReportFull.jsp?rowids=36996) | 1456567_x_at | 1.69 | | ***2.08*** | | 2.55 | |
| [bone morphogenetic protein 2](http://niaid.abcc.ncifcrf.gov/geneReportFull.jsp?rowids=31105) | 1423635_at | -1.18 | | ***-2.26*** | | 2.64 | |
| [transcription factor-like 5 (basic helix-loop-helix)](http://niaid.abcc.ncifcrf.gov/geneReportFull.jsp?rowids=21524) | 1456515_s_at | 1.34 | | ***-2.72*** | | -1.02 | |
| ***Uncategorized*** | |  | |  | |  | |
| [lectin, galactose binding, soluble 3](http://niaid.abcc.ncifcrf.gov/geneReportFull.jsp?rowids=146164) | 1426808_at | 4.16 | | ***10.18*** | | 14.44 | |
| [s100 calcium binding protein a4](http://niaid.abcc.ncifcrf.gov/geneReportFull.jsp?rowids=8890) | 1424542_at | 1.78 | | ***9.01*** | | 8.2 | |
| [glycoprotein (transmembrane) nmb](http://niaid.abcc.ncifcrf.gov/geneReportFull.jsp?rowids=145442) | 1448303_at | 1.93 | | ***8.59*** | | 27.1 | |
| [membrane-spanning 4-domains, subfamily a, member 7](http://niaid.abcc.ncifcrf.gov/geneReportFull.jsp?rowids=148591) | 1424754_at | 2.01 | | ***6.22*** | | 7.5 | |
| [lipocalin 2](http://niaid.abcc.ncifcrf.gov/geneReportFull.jsp?rowids=16296) | 1427747_a_at | 1.38 | | ***6.1*** | | 28.44 | |
| [cd52 antigen](http://niaid.abcc.ncifcrf.gov/geneReportFull.jsp?rowids=6765) | 1460218_at | 4.53 | | ***6.05*** | | 4.51 | |
| [glial fibrillary acidic protein](http://niaid.abcc.ncifcrf.gov/geneReportFull.jsp?rowids=19284) | 1426508_at, 1426509_s_at, 1440142_s_at | 3.05 | | ***5.97*** | | 8.52 | |
| [similar to riken cdna 4933409k07](http://niaid.abcc.ncifcrf.gov/geneReportFull.jsp?rowids=135590) | 1447939_a_at | 4.77 | | ***5.38*** | | 2.87 | |
| [hypothetical loc545604](http://niaid.abcc.ncifcrf.gov/geneReportFull.jsp?rowids=13579) | 1447937_a_at, 1447939_a_at | 4.9 | | ***5.37*** | | 3.06 | |
| [similar to 4933409k07rik protein](http://niaid.abcc.ncifcrf.gov/geneReportFull.jsp?rowids=135587) | 1447937_a_at | 5.02 | | ***5.36*** | | 3.25 | |
| [similar to riken cdna 4933409k07](http://niaid.abcc.ncifcrf.gov/geneReportFull.jsp?rowids=135593) | 1447937_a_at | 5.02 | | ***5.36*** | | 3.25 | |
| [similar to riken cdna 4933409k07](http://niaid.abcc.ncifcrf.gov/geneReportFull.jsp?rowids=135591) | 1447937_a_at | 5.02 | | ***5.36*** | | 3.25 | |
| [similar to riken cdna 4933409k07](http://niaid.abcc.ncifcrf.gov/geneReportFull.jsp?rowids=135589) | 1447937_a_at | 5.02 | | ***5.36*** | | 3.25 | |
| [activating transcription factor 3](http://niaid.abcc.ncifcrf.gov/geneReportFull.jsp?rowids=16699) | 1449363_at | 3.81 | | ***4.86*** | | 3.75 | |
| [riken cdna 4933409k07 gene](http://niaid.abcc.ncifcrf.gov/geneReportFull.jsp?rowids=147976) | 1447937_a_at, 1447938_at, 1447939_a_at | 4.37 | | ***4.83*** | | 2.67 | |
| [bone marrow stromal cell antigen 2](http://niaid.abcc.ncifcrf.gov/geneReportFull.jsp?rowids=18435) | 1424921_at | 2.46 | | ***4.76*** | | 1.69 | |
| [macrophage expressed gene 1](http://niaid.abcc.ncifcrf.gov/geneReportFull.jsp?rowids=16058) | 1427076_at | 4.33 | | ***4.74*** | | 5.66 | |
| [glycoprotein galactosyltransferase alpha 1, 3](http://niaid.abcc.ncifcrf.gov/geneReportFull.jsp?rowids=7347) | 1418483_a_at | 2.77 | | ***4.67*** | | 3.18 | |
| [riken cdna a530050d06 gene](http://niaid.abcc.ncifcrf.gov/geneReportFull.jsp?rowids=37455) | 1435036_at | 1.78 | | ***4.48*** | | 3.5 | |
| [interferon gamma inducible protein 47](http://niaid.abcc.ncifcrf.gov/geneReportFull.jsp?rowids=16587) | 1417292_at | 1.29 | | ***4.46*** | | 1.44 | |
| [ubiquitin specific peptidase 18](http://niaid.abcc.ncifcrf.gov/geneReportFull.jsp?rowids=147072) | 1418191_at | 4.28 | | ***4.4*** | | 2.03 | |
| [riken cdna 1700031k17 gene](http://niaid.abcc.ncifcrf.gov/geneReportFull.jsp?rowids=85440) | 1454314_at | 3.51 | | ***4.27*** | | 2.33 | |
| [hypothetical protein](http://niaid.abcc.ncifcrf.gov/geneReportFull.jsp?rowids=12866) | 1452426_x_at | 2.78 | | ***4.23*** | | 7.97 | |
| [cd5 antigen-like](http://niaid.abcc.ncifcrf.gov/geneReportFull.jsp?rowids=32840) | 1449193_at | -1.45 | | ***4.08*** | | 5.92 | |
| [oncostatin m receptor](http://niaid.abcc.ncifcrf.gov/geneReportFull.jsp?rowids=15302) | 1418674_at, 1418675_at | 1.88 | | ***3.96*** | | 7.29 | |
| [cd48 antigen](http://niaid.abcc.ncifcrf.gov/geneReportFull.jsp?rowids=145994) | 1427301_at | 3.47 | | ***3.91*** | | 2.93 | |
| [b-cell leukemia/lymphoma 2 related protein a1a](http://niaid.abcc.ncifcrf.gov/geneReportFull.jsp?rowids=148308) | 1419004_s_at | 2.68 | | ***3.9*** | | 3.11 | |
| [b-cell leukemia/lymphoma 2 related protein a1b](http://niaid.abcc.ncifcrf.gov/geneReportFull.jsp?rowids=13615) | 1419004_s_at | 2.68 | | ***3.9*** | | 3.11 | |
| [b-cell leukemia/lymphoma 2 related protein a1d](http://niaid.abcc.ncifcrf.gov/geneReportFull.jsp?rowids=3167) | 1419004_s_at | 2.68 | | ***3.9*** | | 3.11 | |
| [tyro protein tyrosine kinase binding protein](http://niaid.abcc.ncifcrf.gov/geneReportFull.jsp?rowids=145147) | 1450792_at | 3.07 | | ***3.88*** | | 3.75 | |
| [lectin, galactoside-binding, soluble, 3 binding protein](http://niaid.abcc.ncifcrf.gov/geneReportFull.jsp?rowids=27527) | 1448380_at | 3.13 | | ***3.77*** | | 3.37 | |
| [riken cdna 0610040j01 gene](http://niaid.abcc.ncifcrf.gov/geneReportFull.jsp?rowids=147083) | 1424404_at | -1.49 | | ***3.76*** | | -1.13 | |
| [receptor transporter protein 4](http://niaid.abcc.ncifcrf.gov/geneReportFull.jsp?rowids=146418) | 1418580_at | 3.46 | | ***3.6*** | | 2.18 | |
| [baculoviral iap repeat-containing 3](http://niaid.abcc.ncifcrf.gov/geneReportFull.jsp?rowids=14228) | 1421392_a_at | 2.34 | | ***3.58*** | | 1.9 | |
| [myosin if](http://niaid.abcc.ncifcrf.gov/geneReportFull.jsp?rowids=149251) | 1429524_at | 1.69 | | ***3.52*** | | 3.61 | |
| [cd72 antigen](http://niaid.abcc.ncifcrf.gov/geneReportFull.jsp?rowids=145428) | 1426112_a_at | 1.38 | | ***3.52*** | | 2.43 | |
| [novel protein similar to extracellular proteinase inhibitor expi](http://niaid.abcc.ncifcrf.gov/geneReportFull.jsp?rowids=37852) | 1436530_at | 1.42 | | ***3.51*** | | 7.47 | |
| [heme oxygenase (decycling) 1](http://niaid.abcc.ncifcrf.gov/geneReportFull.jsp?rowids=12807) | 1448239_at | 1.55 | | ***3.27*** | | 4.38 | |
| [solute carrier family 15, member 3](http://niaid.abcc.ncifcrf.gov/geneReportFull.jsp?rowids=36809) | 1420697_at | 3.33 | | ***3.26*** | | 3.12 | |
| [interferon-induced protein 44](http://niaid.abcc.ncifcrf.gov/geneReportFull.jsp?rowids=35189) | 1423555_a_at | 2.59 | | ***3.25*** | | 3.22 | |
| [,gb:aw215795 /db_xref=gi:6526490 /db_xref=uo99b12.x1 /clone=image:2650655 /fea=est /cnt=6 /tid=mm.89848.1 /tier=consend /stk=3 /ug=mm.89848 /ug_title=ests](http://niaid.abcc.ncifcrf.gov/geneReportFull.jsp?rowids=3166676) | 1442118_at | 2.26 | | ***3.2*** | | 4.83 | |
| [cap, adenylate cyclase-associated protein 1 (yeast)](http://niaid.abcc.ncifcrf.gov/geneReportFull.jsp?rowids=20234) | 1417461_at, 1443141_at, 1417462_at | 3.36 | | ***3.18*** | | 4.42 | |
| [cd84 antigen](http://niaid.abcc.ncifcrf.gov/geneReportFull.jsp?rowids=37956) | 1422875_at | 1.68 | | ***3.17*** | | 3.84 | |
| [riken cdna 2310026i04 gene](http://niaid.abcc.ncifcrf.gov/geneReportFull.jsp?rowids=146316) | 1419315_at | 2.76 | | ***3.15*** | | 1.99 | |
| [cd44 antigen](http://niaid.abcc.ncifcrf.gov/geneReportFull.jsp?rowids=9004) | 1423760_at, 1434376_at | 1.54 | | ***3.12*** | | 4.81 | |
| [ccaat/enhancer binding protein (c/ebp), delta](http://niaid.abcc.ncifcrf.gov/geneReportFull.jsp?rowids=5249) | 1423233_at | 1.65 | | ***3.09*** | | 5.84 | |
| [interferon activated gene 205](http://niaid.abcc.ncifcrf.gov/geneReportFull.jsp?rowids=37635) | 1452349_x_at | 2.38 | | ***3.05*** | | 3.27 | |
| [myeloid cell nuclear differentiation antigen](http://niaid.abcc.ncifcrf.gov/geneReportFull.jsp?rowids=145265) | 1452349_x_at | 2.38 | | ***3.05*** | | 3.27 | |
| [benzodiazepine receptor, peripheral](http://niaid.abcc.ncifcrf.gov/geneReportFull.jsp?rowids=147018) | 1438948_x_at | 1.36 | | ***3.03*** | | 2.08 | |
| [vimentin](http://niaid.abcc.ncifcrf.gov/geneReportFull.jsp?rowids=13620) | 1438118_x_at, 1456292_a_at, 1450641_at | 1.77 | | ***2.82*** | | 5.02 | |
| [cytochrome b-245, beta polypeptide](http://niaid.abcc.ncifcrf.gov/geneReportFull.jsp?rowids=5755) | 1436778_at | 2.17 | | ***2.77*** | | 5.45 | |
| [pentraxin related gene](http://niaid.abcc.ncifcrf.gov/geneReportFull.jsp?rowids=144233) | 1418666_at | 2 | | ***2.72*** | | 3.53 | |
| [cd9 antigen](http://niaid.abcc.ncifcrf.gov/geneReportFull.jsp?rowids=7043) | 1416066_at | 1.79 | | ***2.71*** | | 3.2 | |
| [capping protein (actin filament), gelsolin-like](http://niaid.abcc.ncifcrf.gov/geneReportFull.jsp?rowids=5552) | 1450355_a_at | 1.45 | | ***2.70*** | | 3.86 | |
| [chitinase 3-like 1](http://niaid.abcc.ncifcrf.gov/geneReportFull.jsp?rowids=14931) | 1451537_at | 2.01 | | ***2.65*** | | 4.55 | |
| [expressed sequence au020206](http://niaid.abcc.ncifcrf.gov/geneReportFull.jsp?rowids=102342) | 1433935_at | 2.37 | | ***2.64*** | | 3.93 | |
| [calcium/calmodulin-dependent protein kinase ii, beta](http://niaid.abcc.ncifcrf.gov/geneReportFull.jsp?rowids=4165) | 1455869_at | 3.45 | | ***2.6*** | | 3.31 | |
| [riken cdna 4732466d17 gene](http://niaid.abcc.ncifcrf.gov/geneReportFull.jsp?rowids=146926) | 1438980_x_at | 1.31 | | ***2.57*** | | 3.05 | |
| [membrane-spanning 4-domains, subfamily a, member 11](http://niaid.abcc.ncifcrf.gov/geneReportFull.jsp?rowids=35275) | 1419599_s_at | 1.31 | | ***2.51*** | | 5.37 | |
| [sodium channel, voltage-gated, type xi, alpha](http://niaid.abcc.ncifcrf.gov/geneReportFull.jsp?rowids=20138) | 1420784_at | -1.16 | | ***2.48*** | | -1.3 | |
| [f-box protein 39](http://niaid.abcc.ncifcrf.gov/geneReportFull.jsp?rowids=37531) | 1443698_at | 1.77 | | ***2.45*** | | 1.84 | |
| [renin binding protein](http://niaid.abcc.ncifcrf.gov/geneReportFull.jsp?rowids=144067) | 1450107_a_at | 1.42 | | ***2.45*** | | 2.23 | |
| [triggering receptor expressed on myeloid cells 2c](http://niaid.abcc.ncifcrf.gov/geneReportFull.jsp?rowids=143807) | 1421792_s_at | 2.53 | | ***2.44*** | | 3.65 | |
| [riken cdna c530044c16 gene](http://niaid.abcc.ncifcrf.gov/geneReportFull.jsp?rowids=138712) | 1445247_at | 1.85 | | ***2.39*** | | 2.37 | |
| [expressed sequence ai607873](http://niaid.abcc.ncifcrf.gov/geneReportFull.jsp?rowids=15430) | 1457035_at | 1 | | ***2.38*** | | 2.45 | |
| [similar to tripartite motif protein 34](http://niaid.abcc.ncifcrf.gov/geneReportFull.jsp?rowids=145418) | 1421550_a_at, 1424857_a_at | 2.6 | | ***2.36*** | | 1.42 | |
| [tripartite motif protein 34](http://niaid.abcc.ncifcrf.gov/geneReportFull.jsp?rowids=145277) | 1421550_a_at, 1424857_a_at | 2.6 | | ***2.36*** | | 1.42 | |
| [three prime repair exonuclease 1](http://niaid.abcc.ncifcrf.gov/geneReportFull.jsp?rowids=33572) | 1450672_a_at | 1.55 | | ***2.36*** | | -1.5 | |
| [allograft inflammatory factor 1](http://niaid.abcc.ncifcrf.gov/geneReportFull.jsp?rowids=13193) | 1418204_s_at | 3.31 | | ***2.33*** | | 1.9 | |
| [dna segment, chr 14, erato doi 668, expressed](http://niaid.abcc.ncifcrf.gov/geneReportFull.jsp?rowids=35182) | 1438868_at | 2.09 | | ***2.32*** | | 1.19 | |
| [fxyd domain-containing ion transport regulator 1](http://niaid.abcc.ncifcrf.gov/geneReportFull.jsp?rowids=7076) | 1421374_a_at | 1.45 | | ***2.28*** | | 2.61 | |
| [carbonic anhydrase 13](http://niaid.abcc.ncifcrf.gov/geneReportFull.jsp?rowids=25741) | 1421308_at | 1.01 | | ***2.28*** | | 1.79 | |
| [cytochrome p450, family 3, subfamily a, polypeptide 13](http://niaid.abcc.ncifcrf.gov/geneReportFull.jsp?rowids=19495) | 1419523_at | 1.47 | | ***2.26*** | | 1.65 | |
| [udp-n-acetyl-alpha-d-galactosamine:polypeptide n-acetylgalactosaminyltransferase-like 2](http://niaid.abcc.ncifcrf.gov/geneReportFull.jsp?rowids=26448) | 1429235_at | 1.25 | | ***2.24*** | | 2.34 | |
| [expressed sequence ai451617](http://niaid.abcc.ncifcrf.gov/geneReportFull.jsp?rowids=26580) | 1456494_a_at | 1.81 | | ***2.22*** | | 1.86 | |
| [tripartite motif protein 30](http://niaid.abcc.ncifcrf.gov/geneReportFull.jsp?rowids=23840) | 1451860_a_at, 1456494_a_at | 2.06 | | ***2.22*** | | 2.05 | |
| [macrophage scavenger receptor 2](http://niaid.abcc.ncifcrf.gov/geneReportFull.jsp?rowids=36899) | 1448891_at | 1.82 | | ***2.22*** | | 2.35 | |
| [lymphocyte cytosolic protein 1](http://niaid.abcc.ncifcrf.gov/geneReportFull.jsp?rowids=146073) | 1415983_at | 1.97 | | ***2.21*** | | 2.04 | |
| [poly (adp-ribose) polymerase family, member 3](http://niaid.abcc.ncifcrf.gov/geneReportFull.jsp?rowids=16271) | 1426210_x_at, 1451969_s_at | 1.83 | | ***2.19*** | | 3.15 | |
| [carboxypeptidase x 2 (m14 family)](http://niaid.abcc.ncifcrf.gov/geneReportFull.jsp?rowids=26497) | 1460248_at | 1.21 | | ***2.18*** | | 1.36 | |
| [fibrinogen-like protein 2](http://niaid.abcc.ncifcrf.gov/geneReportFull.jsp?rowids=12644) | 1421854_at | 1.02 | | ***2.18*** | | 1.73 | |
| [heat shock protein, alpha-crystallin-related, b6](http://niaid.abcc.ncifcrf.gov/geneReportFull.jsp?rowids=26485) | 1436332_at | 1.42 | | ***2.16*** | | 2.9 | |
| [rap guanine nucleotide exchange factor (gef) 3](http://niaid.abcc.ncifcrf.gov/geneReportFull.jsp?rowids=146414) | 1438590_at | 1.29 | | ***2.16*** | | 2.19 | |
| [kinesin family member 1c](http://niaid.abcc.ncifcrf.gov/geneReportFull.jsp?rowids=6710) | 1424747_at | 1.39 | | ***2.12*** | | 1.57 | |
| [pdz and lim domain 4](http://niaid.abcc.ncifcrf.gov/geneReportFull.jsp?rowids=19295) | 1417928_at | 1.97 | | ***2.12*** | | 2.23 | |
| [thrombospondin 2](http://niaid.abcc.ncifcrf.gov/geneReportFull.jsp?rowids=37065) | 1422571_at | 1.36 | | ***2.09*** | | 2.3 | |
| [plasmacytoma variant translocation 1](http://niaid.abcc.ncifcrf.gov/geneReportFull.jsp?rowids=7156) | 1427140_at | 1.21 | | ***2.09*** | | 2.85 | |
| [tripartite motif protein 25](http://niaid.abcc.ncifcrf.gov/geneReportFull.jsp?rowids=15257) | 1419879_s_at, 1425974_a_at | 1.38 | | ***2.07*** | | 1.46 | |
| [udp glucuronosyltransferase 1 family, polypeptide a7c](http://niaid.abcc.ncifcrf.gov/geneReportFull.jsp?rowids=144007) | 1426260_a_at | 1.68 | | ***2.07*** | | 2.91 | |
| [udp glucuronosyltransferase 1 family, polypeptide a2](http://niaid.abcc.ncifcrf.gov/geneReportFull.jsp?rowids=3809) | 1426260_a_at | 1.68 | | ***2.07*** | | 2.91 | |
| [udp glucuronosyltransferase 1 family, polypeptide a9](http://niaid.abcc.ncifcrf.gov/geneReportFull.jsp?rowids=27051) | 1426260_a_at | 1.68 | | ***2.07*** | | 2.91 | |
| [zinc finger protein 306](http://niaid.abcc.ncifcrf.gov/geneReportFull.jsp?rowids=5348) | 1437892_at | 1.51 | | ***2.07*** | | -1.05 | |
| [udp glucuronosyltransferase 1 family, polypeptide a5](http://niaid.abcc.ncifcrf.gov/geneReportFull.jsp?rowids=27362) | 1426260_a_at | 1.68 | | ***2.07*** | | 2.91 | |
| [hypothetical protein mgc36247](http://niaid.abcc.ncifcrf.gov/geneReportFull.jsp?rowids=3348) | 1426260_a_at | 1.68 | | ***2.07*** | | 2.91 | |
| [udp glycosyltransferase 1 family, polypeptide a10](http://niaid.abcc.ncifcrf.gov/geneReportFull.jsp?rowids=22974) | 1426260_a_at | 1.68 | | ***2.07*** | | 2.91 | |
| [udp glucuronosyltransferase 1 family, polypeptide a6a](http://niaid.abcc.ncifcrf.gov/geneReportFull.jsp?rowids=146268) | 1426260_a_at | 1.68 | | ***2.07*** | | 2.91 | |
| [riken cdna 0610011i04 gene](http://niaid.abcc.ncifcrf.gov/geneReportFull.jsp?rowids=13418) | 1425603_at, 1441811_x_at | 1.41 | | ***2.06*** | | 2.78 | |
| [riken cdna 4933439c20 gene](http://niaid.abcc.ncifcrf.gov/geneReportFull.jsp?rowids=149075) | 1453145_at, 1453144_at | 2.22 | | ***2.06*** | | 1.34 | |
| [phosphatidic acid phosphatase type 2b](http://niaid.abcc.ncifcrf.gov/geneReportFull.jsp?rowids=12962) | 1446850_at | 1.22 | | ***2.05*** | | -1.05 | |
| [s100 calcium binding protein a11 (calizzarin)](http://niaid.abcc.ncifcrf.gov/geneReportFull.jsp?rowids=25580) | 1460351_at | 1.51 | | ***2.05*** | | 2.24 | |
| [immunity-related gtpase family, m](http://niaid.abcc.ncifcrf.gov/geneReportFull.jsp?rowids=27981) | 1418825_at | 1.88 | | ***2.04*** | | 1.86 | |
| [pyrimidinergic receptor p2y, g-protein coupled, 6](http://niaid.abcc.ncifcrf.gov/geneReportFull.jsp?rowids=7979) | 1425214_at | 1.57 | | ***2.04*** | | 2.45 | |
| [annexin a4](http://niaid.abcc.ncifcrf.gov/geneReportFull.jsp?rowids=147692) | 1424176_a_at | 1.52 | | ***2.04*** | | 2.83 | |
| [caspase 12](http://niaid.abcc.ncifcrf.gov/geneReportFull.jsp?rowids=147683) | 1449297_at | 1.34 | | ***2.04*** | | 2.72 | |
| [actin related protein 2/3 complex, subunit 1b](http://niaid.abcc.ncifcrf.gov/geneReportFull.jsp?rowids=26617) | 1416226_at | 1.59 | | ***2.02*** | | 2.46 | |
| [tigger transposable element derived 5](http://niaid.abcc.ncifcrf.gov/geneReportFull.jsp?rowids=15527) | 1460743_at | 1.01 | | ***2.02*** | | -1.81 | |
| [riken cdna 5730465c04 gene](http://niaid.abcc.ncifcrf.gov/geneReportFull.jsp?rowids=12757) | 1449221_a_at | 1 | | ***2.01*** | | 1.38 | |
| [,gb:bb522668 /db_xref=gi:16443707 /db_xref=bb522668 /clone=d930008n22 /fea=est /cnt=2 /tid=mm.209697.1 /tier=consend /stk=2 /ug=mm.209697 /ug_title=ests](http://niaid.abcc.ncifcrf.gov/geneReportFull.jsp?rowids=3168474) | 1459235_at | 1.64 | | ***-2.03*** | | -1.22 | |
| [centrosome and spindle pole associated protein 1](http://niaid.abcc.ncifcrf.gov/geneReportFull.jsp?rowids=13977) | 1457971_at | -1.27 | | ***-2.03*** | | -1.27 | |
| [,gb:av266436 /db_xref=gi:16389471 /db_xref=av266436 /clone=4930517k17 /fea=est /cnt=10 /tid=mm.109571.1 /tier=stack /stk=8 /ug=mm.109571 /ug_title=ests](http://niaid.abcc.ncifcrf.gov/geneReportFull.jsp?rowids=3166313) | 1439177_at | -1.24 | | ***-2.07*** | | -1.13 | |
| [nima (never in mitosis gene a)-related expressed kinase 1](http://niaid.abcc.ncifcrf.gov/geneReportFull.jsp?rowids=144648) | 1453612_at | -1.03 | | ***-2.11*** | | -1.49 | |
| [coiled-coil domain containing 65](http://niaid.abcc.ncifcrf.gov/geneReportFull.jsp?rowids=144393) | 1451578_at | 1.08 | | ***-2.13*** | | -1.09 | |
| [riken cdna 4833426j09 gene](http://niaid.abcc.ncifcrf.gov/geneReportFull.jsp?rowids=35174) | 1441285_at | -1.1 | | ***-2.14*** | | 1.03 | |
| [eps8-like 1](http://niaid.abcc.ncifcrf.gov/geneReportFull.jsp?rowids=13160) | 1431821_a_at | 1.27 | | ***-2.15*** | | -2.09 | |
| [expressed sequence ai256693](http://niaid.abcc.ncifcrf.gov/geneReportFull.jsp?rowids=14612) | 1416494_at | -2.1 | | ***-2.17*** | | -2.39 | |
| [endothelin converting enzyme 1](http://niaid.abcc.ncifcrf.gov/geneReportFull.jsp?rowids=5514) | 1441423_at | -1.07 | | ***-2.19*** | | -1.24 | |
| [solute carrier family 25 (mitochondrial carrier, ornithine transporter) member 2](http://niaid.abcc.ncifcrf.gov/geneReportFull.jsp?rowids=30310) | 1425013_at | 1.2 | | ***-2.22*** | | -1.24 | |
| [,gb:bf661182 /db_xref=gi:11926316 /db_xref=maa73e01.x1 /clone=image:3822432 /fea=est /cnt=5 /tid=mm.139152.1 /tier=consend /stk=3 /ug=mm.139152 /ug_title=ests](http://niaid.abcc.ncifcrf.gov/geneReportFull.jsp?rowids=3166707) | 1442343_at | 1.01 | | ***-2.25*** | | -1.27 | |
| [,gb:bg063381 /db_xref=gi:12545944 /db_xref=h3006f02-3 /clone=h3006f02 /fea=est /cnt=3 /tid=mm.171805.1 /tier=consend /stk=2 /ug=mm.171805 /ug_title=ests](http://niaid.abcc.ncifcrf.gov/geneReportFull.jsp?rowids=3167212) | 1445132_at | -1.72 | | ***-2.39*** | | 1.64 | |
| [dedicator of cytokinesis 9](http://niaid.abcc.ncifcrf.gov/geneReportFull.jsp?rowids=5420) | 1439200_x_at | -1.66 | | ***-2.45*** | | -1.92 | |
| [wd repeat and fyve domain containing 1](http://niaid.abcc.ncifcrf.gov/geneReportFull.jsp?rowids=38064) | 1437358_at, 1435588_at, 1424749_at | -2.55 | | ***-2.49*** | | -2.17 | |
| [gamma-aminobutyric acid (gaba-a) receptor, subunit alpha 2](http://niaid.abcc.ncifcrf.gov/geneReportFull.jsp?rowids=5213) | 1421738_at, 1455444_at, 1443865_at | -2.4 | | ***-2.58*** | | -2.41 | |
| [riken cdna c330013j21 gene](http://niaid.abcc.ncifcrf.gov/geneReportFull.jsp?rowids=6665) | 1420318_at | 1.53 | | ***-2.63*** | | -1.11 | |
| [transmembrane protein 40](http://niaid.abcc.ncifcrf.gov/geneReportFull.jsp?rowids=148465) | 1424966_at | -1.82 | | ***-2.79*** | | -1.36 | |
| [erythroid differentiation regulator 1](http://niaid.abcc.ncifcrf.gov/geneReportFull.jsp?rowids=4417) | 1439200_x_at, 1452406_x_at | -2.2 | | ***-3.01*** | | -2.43 | |
| [hypothetical protein](http://niaid.abcc.ncifcrf.gov/geneReportFull.jsp?rowids=19195) | 1427820_at | -2.86 | | ***-3.34*** | | -3.21 | |
| [,gb:bb276544 /db_xref=gi:8973565 /db_xref=bb276544 /clone=a830097p08 /fea=est /cnt=7 /tid=mm.71877.1 /tier=consend /stk=5 /ug=mm.71877 /ug_title=ests, weakly similar to pol2 mouse retrovirus-related pol polyprotein (m.musculus)](http://niaid.abcc.ncifcrf.gov/geneReportFull.jsp?rowids=3166422) | 1440342_at | -2.4 | | ***-4.9*** | | -1.9 | |
