# Supplementary material for: Gene expression profiling in a mouse model of infantile neuronal ceroid lipofuscinosis reveals upregulation of immediate early genes and mediators of the inflammatory response
Source: BMC Neurosci. 2007 Nov 16;8:95. doi: 10.1186/1471-2202-8-95 (PMC2204004; doi:10.1186/1471-2202-8-95)
Supplement: Additional File 6 — Enriched GO (Gene Ontology) categories (and enrichment scores) for genes significantly regulated at 8 months (but not significant at 3 and 5 months) (Microsoft Word table). All genes showing a statistically significant difference between knockout and wild-type at 8 months but not at 3 and 5 months are shown. Genes are first grouped according to Gene Ontology category, and uncategorized genes are also shown. Of note, several genes show evidence of regulation at 3 and 5 months (as well as 8 months), but in these instances, the difference reached statistical significance only at 8 months. [file 1471-2202-8-95-S6.doc]

| Additional File 6 |  |  |  |  |
| --- | --- | --- | --- | --- |
| **Enriched GO (Gene Ontology) categories (and enrichment scores) for genes significantly regulated at 8 months (but not significant at 3 and 5 months)s** | | | | |
| ***Gene family and gene name*** | ***Affy-ID*** | ***Fold change KO:WT*** | | |
|  |  | ***3 mo*** | ***5 mo*** | ***8 mo*** |
| ***purine nucleotide binding (2.5E-4)*** | |  |  |  |
| [riken cdna 9330153b10 gene](http://niaid.abcc.ncifcrf.gov/geneReportFull.jsp?rowids=117218) | 1459956_at | -1.33 | 1.06 | ***2.89*** |
| [yamaguchi sarcoma viral (v-yes) oncogene homolog 1](http://niaid.abcc.ncifcrf.gov/geneReportFull.jsp?rowids=146989) | 1456843_at | -1.08 | 1.47 | ***2.65*** |
| [calcium/calmodulin-dependent protein kinase iv](http://niaid.abcc.ncifcrf.gov/geneReportFull.jsp?rowids=9571) | 1438960_at | 1.17 | -1.11 | ***2.49*** |
| [endothelial differentiation, sphingolipid g-protein-coupled receptor, 3](http://niaid.abcc.ncifcrf.gov/geneReportFull.jsp?rowids=3654) | 1460661_at | 1.9 | 2.21 | ***2.47*** |
| [est x83316](http://niaid.abcc.ncifcrf.gov/geneReportFull.jsp?rowids=29252) | 1449060_at | 1.47 | 1.09 | ***2.43*** |
| [ras homolog gene family, member c](http://niaid.abcc.ncifcrf.gov/geneReportFull.jsp?rowids=26653) | 1448605_at | 1.28 | 1.51 | ***2.41*** |
| [era (g-protein)-like 1 (e. coli)](http://niaid.abcc.ncifcrf.gov/geneReportFull.jsp?rowids=59910) | 1458914_at | 1.81 | -1.1 | ***2.34*** |
| [braf transforming gene](http://niaid.abcc.ncifcrf.gov/geneReportFull.jsp?rowids=10502) | 1441740_at | 1.07 | 1.12 | ***2.3*** |
| [atp-binding cassette, sub-family a (abc1), member 1](http://niaid.abcc.ncifcrf.gov/geneReportFull.jsp?rowids=31084) | 1421839_at | 1.69 | 1.86 | ***2.29*** |
| [gtpase, imap family member 4](http://niaid.abcc.ncifcrf.gov/geneReportFull.jsp?rowids=31919) | 1424375_s_at | 2.22 | 1.14 | ***2.26*** |
| [transglutaminase 2, c polypeptide](http://niaid.abcc.ncifcrf.gov/geneReportFull.jsp?rowids=15300) | 1433428_x_at | 1.37 | 1.58 | ***2.18*** |
| [riken cdna 1300010f03 gene](http://niaid.abcc.ncifcrf.gov/geneReportFull.jsp?rowids=24603) | 1431800_at | -1.34 | -1.17 | ***2.11*** |
| [kinesin family member 18a](http://niaid.abcc.ncifcrf.gov/geneReportFull.jsp?rowids=3337) | 1424107_at | -1.18 | 1.74 | ***2.07*** |
| [death associated protein kinase 1](http://niaid.abcc.ncifcrf.gov/geneReportFull.jsp?rowids=5250) | 1445032_at | -1.21 | 1.3 | ***2.05*** |
| [thyroid hormone receptor interactor 13](http://niaid.abcc.ncifcrf.gov/geneReportFull.jsp?rowids=34153) | 1429295_s_at | 1.38 | -1.17 | ***2.04*** |
| [deah (asp-glu-ala-his) box polypeptide 29](http://niaid.abcc.ncifcrf.gov/geneReportFull.jsp?rowids=15426) | 1439076_at | -1.14 | -2.02 | ***-2*** |
| [kinesin family member 5a](http://niaid.abcc.ncifcrf.gov/geneReportFull.jsp?rowids=16472) | 1450249_s_at | 1.04 | -1.27 | ***-2.01*** |
| [kinesin family member 5c](http://niaid.abcc.ncifcrf.gov/geneReportFull.jsp?rowids=36465) | 1450249_s_at | 1.04 | -1.27 | ***-2.01*** |
| [diras family, gtp-binding ras-like 2](http://niaid.abcc.ncifcrf.gov/geneReportFull.jsp?rowids=37863) | 1445473_at | 1.02 | -1.29 | ***-2.09*** |
| [atp-binding cassette, sub-family a (abc1), member 8a](http://niaid.abcc.ncifcrf.gov/geneReportFull.jsp?rowids=86191) | 1427371_at | -1.55 | -1.22 | ***-2.12*** |
| [heat shock protein 12a](http://niaid.abcc.ncifcrf.gov/geneReportFull.jsp?rowids=4560) | 1447222_at | 1.19 | -1.12 | ***-2.33*** |
| [u2af homology motif (uhm) kinase 1](http://niaid.abcc.ncifcrf.gov/geneReportFull.jsp?rowids=39680) | 1450500_at | 1.04 | -1.32 | ***-2.45*** |
| [cdna sequence bc030499](http://niaid.abcc.ncifcrf.gov/geneReportFull.jsp?rowids=147876) | 1444656_at | -1.34 | -1.32 | ***-2.5*** |
| [chaperonin subunit 4 (delta)](http://niaid.abcc.ncifcrf.gov/geneReportFull.jsp?rowids=36559) | 1415868_at | -1.2 | -1.22 | ***-2.6*** |
| [triple functional domain (ptprf interacting)](http://niaid.abcc.ncifcrf.gov/geneReportFull.jsp?rowids=145563) | 1438099_at | 1.17 | -1.57 | ***-2.62*** |
| [eph receptor a3](http://niaid.abcc.ncifcrf.gov/geneReportFull.jsp?rowids=26506) | 1425574_at | 1.68 | -1.01 | ***-2.73*** |
| [myosin, heavy polypeptide 3, skeletal muscle, embryonic](http://niaid.abcc.ncifcrf.gov/geneReportFull.jsp?rowids=31940) | 1427115_at | -1.2 | -1.81 | ***-2.95*** |
| [myosin, heavy polypeptide 6, cardiac muscle, alpha](http://niaid.abcc.ncifcrf.gov/geneReportFull.jsp?rowids=27090) | 1448554_s_at | 1.19 | -1.28 | ***-3.06*** |
| ***Negative regulation of cellular process (3.7E-3)*** | |  |  |  |
| [regulator of g-protein signaling 1](http://niaid.abcc.ncifcrf.gov/geneReportFull.jsp?rowids=27424) | 1417601_at | 1.18 | 1.96 | ***4.95*** |
| [insulin-like growth factor 1](http://niaid.abcc.ncifcrf.gov/geneReportFull.jsp?rowids=144284) | 1452014_a_at | 1.61 | 1.61 | ***3.16*** |
| [fc receptor, igg, low affinity iib](http://niaid.abcc.ncifcrf.gov/geneReportFull.jsp?rowids=26631) | 1455332_x_at | 1.19 | 2.45 | ***2.86*** |
| [ciliary neurotrophic factor](http://niaid.abcc.ncifcrf.gov/geneReportFull.jsp?rowids=15988) | 1422033_a_at | 1.44 | 1.53 | ***2.83*** |
| [ccaat/enhancer binding protein (c/ebp), beta](http://niaid.abcc.ncifcrf.gov/geneReportFull.jsp?rowids=13626) | 1427844_a_at | 1.25 | 1.4 | ***2.58*** |
| [nitric oxide synthase trafficker](http://niaid.abcc.ncifcrf.gov/geneReportFull.jsp?rowids=144637) | 1441075_at | -1.24 | 1.47 | ***2.35*** |
| [regulator of g-protein signaling 2](http://niaid.abcc.ncifcrf.gov/geneReportFull.jsp?rowids=5269) | 1443250_at | 1.23 | -1.02 | ***2.23*** |
| [ccaat/enhancer binding protein (c/ebp), alpha](http://niaid.abcc.ncifcrf.gov/geneReportFull.jsp?rowids=4171) | 1418982_at | 1.09 | 1.39 | ***2.21*** |
| [kruppel-like factor 4 (gut)](http://niaid.abcc.ncifcrf.gov/geneReportFull.jsp?rowids=9018) | 1417394_at | 1.27 | 1.75 | ***2.12*** |
| [nuclear factor of kappa light chain gene enhancer in b-cells inhibitor, alpha](http://niaid.abcc.ncifcrf.gov/geneReportFull.jsp?rowids=144696) | 1420089_at | -1.03 | 1.2 | ***2.10*** |
| [death associated protein kinase 1](http://niaid.abcc.ncifcrf.gov/geneReportFull.jsp?rowids=5250) | 1445032_at | -1.21 | 1.3 | ***2.05*** |
| [arachidonate 8-lipoxygenase](http://niaid.abcc.ncifcrf.gov/geneReportFull.jsp?rowids=28220) | 1425376_at | -1.52 | 1.15 | ***-2.16*** |
| [midline 1](http://niaid.abcc.ncifcrf.gov/geneReportFull.jsp?rowids=12671) | 1422055_at | -1.89 | -1.52 | ***-2.24*** |
| [u2af homology motif (uhm) kinase 1](http://niaid.abcc.ncifcrf.gov/geneReportFull.jsp?rowids=39680) | 1450500_at | 1.04 | -1.32 | ***-2.45*** |
| [hairy and enhancer of split 5 (drosophila)](http://niaid.abcc.ncifcrf.gov/geneReportFull.jsp?rowids=34748) | 1456010_x_at | 1.18 | -1.14 | ***-3.27*** |
| ***Intracellular transport (1.3E-2)*** | |  |  |  |
| [uncoupling protein 2 (mitochondrial, proton carrier)](http://niaid.abcc.ncifcrf.gov/geneReportFull.jsp?rowids=144647) | 1459741_x_at | -1.76 | -1 | ***3.16*** |
| [nucleoporin 214](http://niaid.abcc.ncifcrf.gov/geneReportFull.jsp?rowids=15053) | 1456503_at | -1.04 | 1.1 | ***2.62*** |
| [solute carrier family 1 (glial high affinity glutamate transporter), member 3](http://niaid.abcc.ncifcrf.gov/geneReportFull.jsp?rowids=33996) | 1440491_at | -1.3 | 1.21 | ***2.60*** |
| [calcium/calmodulin-dependent protein kinase iv](http://niaid.abcc.ncifcrf.gov/geneReportFull.jsp?rowids=9571) | 1438960_at | 1.17 | -1.11 | ***2.49*** |
| [est x83316](http://niaid.abcc.ncifcrf.gov/geneReportFull.jsp?rowids=29252) | 1449060_at | 1.47 | 1.09 | ***2.43*** |
| [riken cdna 1700081h05 gene](http://niaid.abcc.ncifcrf.gov/geneReportFull.jsp?rowids=27586) | 1442697_at | -1.03 | -1.03 | ***2.41*** |
| [nuclear factor of kappa light chain gene enhancer in b-cells inhibitor, alpha](http://niaid.abcc.ncifcrf.gov/geneReportFull.jsp?rowids=144696) | 1420089_at | -1.03 | 1.2 | ***2.10*** |
| [kinesin family member 18a](http://niaid.abcc.ncifcrf.gov/geneReportFull.jsp?rowids=3337) | 1424107_at | -1.18 | 1.74 | ***2.07*** |
| [kinesin family member 5a](http://niaid.abcc.ncifcrf.gov/geneReportFull.jsp?rowids=16472) | 1450249_s_at | 1.04 | -1.27 | ***-2.01*** |
| [kinesin family member 5c](http://niaid.abcc.ncifcrf.gov/geneReportFull.jsp?rowids=36465) | 1450249_s_at | 1.04 | -1.27 | ***-2.01*** |
| [solute carrier family 25, member 32](http://niaid.abcc.ncifcrf.gov/geneReportFull.jsp?rowids=18043) | 1429462_at | 1.19 | -1.66 | ***-2.06*** |
| [golgi snap receptor complex member 1](http://niaid.abcc.ncifcrf.gov/geneReportFull.jsp?rowids=144179) | 1448256_at | 1.22 | -1.06 | ***-2.18*** |
| [u2af homology motif (uhm) kinase 1](http://niaid.abcc.ncifcrf.gov/geneReportFull.jsp?rowids=39680) | 1450500_at | 1.04 | -1.32 | ***-2.45*** |
| ***positive regulation of cellular process (1.8E-2)*** | |  |  |  |
| [fc receptor, igg, low affinity iib](http://niaid.abcc.ncifcrf.gov/geneReportFull.jsp?rowids=26631) | 1455332_x_at | 1.19 | 2.45 | ***2.86*** |
| [ciliary neurotrophic factor](http://niaid.abcc.ncifcrf.gov/geneReportFull.jsp?rowids=15988) | 1422033_a_at | 1.44 | 1.53 | ***2.83*** |
| [ccaat/enhancer binding protein (c/ebp), beta](http://niaid.abcc.ncifcrf.gov/geneReportFull.jsp?rowids=13626) | 1427844_a_at | 1.25 | 1.4 | ***2.58*** |
| [endothelial differentiation, sphingolipid g-protein-coupled receptor, 3](http://niaid.abcc.ncifcrf.gov/geneReportFull.jsp?rowids=3654) | 1460661_at | 1.9 | 2.21 | ***2.47*** |
| [icos ligand](http://niaid.abcc.ncifcrf.gov/geneReportFull.jsp?rowids=9710) | 1419212_at | 1.14 | 1.21 | ***2.45*** |
| [thyroid hormone receptor associated protein 1](http://niaid.abcc.ncifcrf.gov/geneReportFull.jsp?rowids=28243) | 1436904_at | -1.06 | 1.14 | ***2.27*** |
| [transglutaminase 2, c polypeptide](http://niaid.abcc.ncifcrf.gov/geneReportFull.jsp?rowids=15300) | 1433428_x_at | 1.37 | 1.58 | ***2.18*** |
| [death associated protein kinase 1](http://niaid.abcc.ncifcrf.gov/geneReportFull.jsp?rowids=5250) | 1445032_at | -1.21 | 1.3 | ***2.05*** |
| [arachidonate 8-lipoxygenase](http://niaid.abcc.ncifcrf.gov/geneReportFull.jsp?rowids=28220) | 1425376_at | -1.52 | 1.15 | ***-2.16*** |
| [phosphodiesterase 6g, cgmp-specific, rod, gamma](http://niaid.abcc.ncifcrf.gov/geneReportFull.jsp?rowids=11847) | 1425100_a_at | 1.68 | 1.86 | ***-2.34*** |
| [atonal homolog 1 (drosophila)](http://niaid.abcc.ncifcrf.gov/geneReportFull.jsp?rowids=20329) | 1449822_at | -1.96 | 1.28 | ***-2.43*** |
| [nuclear factor of activated t-cells 5](http://niaid.abcc.ncifcrf.gov/geneReportFull.jsp?rowids=4025) | 1451921_a_at | 1.18 | 1.08 | ***-2.78*** |
| ***Cell adhesion (1.8E-2)*** | |  |  |  |
| [intercellular adhesion molecule](http://niaid.abcc.ncifcrf.gov/geneReportFull.jsp?rowids=147039) | 1424067_at | 1.84 | 2.45 | ***5.42*** |
| [cd44 antigen](http://niaid.abcc.ncifcrf.gov/geneReportFull.jsp?rowids=9004) | 1452483_a_at | 1.24 | 2.36 | ***4.45*** |
| [transforming growth factor, beta induced](http://niaid.abcc.ncifcrf.gov/geneReportFull.jsp?rowids=144470) | 1448123_s_at | 1.17 | 1.48 | ***3.37*** |
| [annexin a9](http://niaid.abcc.ncifcrf.gov/geneReportFull.jsp?rowids=38836) | 1431554_a_at | 1.74 | 1.15 | ***2.95*** |
| [procollagen, type iv, alpha 5](http://niaid.abcc.ncifcrf.gov/geneReportFull.jsp?rowids=10741) | 1425476_at | 1.23 | 1.67 | ***2.85*** |
| [ae binding protein 1](http://niaid.abcc.ncifcrf.gov/geneReportFull.jsp?rowids=27049) | 1450637_a_at | 1.31 | 2.86 | ***2.49*** |
| [melanoma inhibitory activity 1](http://niaid.abcc.ncifcrf.gov/geneReportFull.jsp?rowids=37429) | 1419608_a_at | -1.45 | -1.25 | ***2.31*** |
| [procollagen, type ix, alpha 2](http://niaid.abcc.ncifcrf.gov/geneReportFull.jsp?rowids=11070) | 1450673_at | 1.78 | -1.08 | ***2.21*** |
| [protein s (alpha)](http://niaid.abcc.ncifcrf.gov/geneReportFull.jsp?rowids=16901) | 1426246_at | 1.64 | 1.67 | ***2.18*** |
| [transglutaminase 2, c polypeptide](http://niaid.abcc.ncifcrf.gov/geneReportFull.jsp?rowids=15300) | 1433428_x_at | 1.37 | 1.58 | ***2.18*** |
| [poliovirus receptor-related 3](http://niaid.abcc.ncifcrf.gov/geneReportFull.jsp?rowids=5065) | 1417319_at | 1.31 | -1.02 | ***-2.88*** |
| [hairy and enhancer of split 5 (drosophila)](http://niaid.abcc.ncifcrf.gov/geneReportFull.jsp?rowids=34748) | 1456010_x_at | 1.18 | -1.14 | ***-3.27*** |
| ***Uncategorized*** | |  |  |  |
| [,gb:aw060425 /db_xref=gi:6008176 /db_xref=ui-m-bh1-anj-c-10-0-ui.s1 /clone=ui-m-bh1-anj-c-10-0-ui /fea=est /cnt=3 /tid=mm.59121.1 /tier=consend /stk=2 /ug=mm.59121 /ug_title=ests](http://niaid.abcc.ncifcrf.gov/geneReportFull.jsp?rowids=3167341) | 1445656_at | -1.42 | 1.21 | ***4.49*** |
| [membrane-spanning 4-domains, subfamily a, member 6d](http://niaid.abcc.ncifcrf.gov/geneReportFull.jsp?rowids=18909) | 1419598_at | 1.77 | 2.02 | ***4.37*** |
| [heat shock protein 1](http://niaid.abcc.ncifcrf.gov/geneReportFull.jsp?rowids=144368) | 1422943_a_at, 1425964_x_at | 1.05 | 1.54 | ***4.12*** |
| [transmembrane inner ear](http://niaid.abcc.ncifcrf.gov/geneReportFull.jsp?rowids=10901) | 1441926_x_at | 1.92 | 1.94 | ***3.8*** |
| [riken cdna 4632413k17 gene](http://niaid.abcc.ncifcrf.gov/geneReportFull.jsp?rowids=147305) | 1431696_at | 1.35 | -1.3 | ***3.66*** |
| [solute carrier family 11 (proton-coupled divalent metal ion transporters), member 1](http://niaid.abcc.ncifcrf.gov/geneReportFull.jsp?rowids=145788) | 1420361_at | 1.84 | 1.97 | ***3.64*** |
| [riken cdna 9030217h17 gene](http://niaid.abcc.ncifcrf.gov/geneReportFull.jsp?rowids=3653) | 1434089_at | -1.28 | 1.31 | ***3.35*** |
| [polymerase (rna) ii (dna directed) polypeptide a](http://niaid.abcc.ncifcrf.gov/geneReportFull.jsp?rowids=14915) | 1458710_at | 1.27 | 1.13 | ***3.22*** |
| [cdna sequence af397014](http://niaid.abcc.ncifcrf.gov/geneReportFull.jsp?rowids=144729) | 1421422_at | 1.33 | -1.01 | ***3.21*** |
| [metallothionein 2](http://niaid.abcc.ncifcrf.gov/geneReportFull.jsp?rowids=32045) | 1428942_at | -1.23 | 1.35 | ***3.19*** |
| [cytochrome b reductase 1](http://niaid.abcc.ncifcrf.gov/geneReportFull.jsp?rowids=143791) | 1460604_at | -1.04 | 1.59 | ***3.1*** |
| [,gb:bg063579 /db_xref=gi:12546142 /db_xref=h3009a05-3 /clone=h3009a05 /fea=est /cnt=3 /tid=mm.102551.1 /tier=consend /stk=3 /ug=mm.102551 /ug_title=ests](http://niaid.abcc.ncifcrf.gov/geneReportFull.jsp?rowids=3166839) | 1442996_x_at | 1.27 | -1.27 | ***3.03*** |
| [cytochrome b-245, beta polypeptide](http://niaid.abcc.ncifcrf.gov/geneReportFull.jsp?rowids=5755) | 1436779_at | 1.8 | 2.24 | ***3.02*** |
| [cd300 antigen like family member f](http://niaid.abcc.ncifcrf.gov/geneReportFull.jsp?rowids=3987) | 1427994_at | 1.86 | 2.32 | ***2.93*** |
| [riken cdna 2810032e02 gene](http://niaid.abcc.ncifcrf.gov/geneReportFull.jsp?rowids=9119) | 1455971_at | -1.45 | -1.47 | ***2.88*** |
| [,gb:be980823 /db_xref=gi:10649280 /db_xref=ui-m-bg2-bcn-e-01-0-ui.s1 /clone=ui-m-bg2-bcn-e-01-0-ui /fea=est /cnt=7 /tid=mm.131143.1 /tier=consend /stk=2 /ug=mm.131143 /ug_title=ests](http://niaid.abcc.ncifcrf.gov/geneReportFull.jsp?rowids=3166997) | 1443998_at | -1.14 | -1.05 | ***2.83*** |
| [apolipoprotein b editing complex 1](http://niaid.abcc.ncifcrf.gov/geneReportFull.jsp?rowids=35220) | 1451755_a_at | 1.47 | 1.82 | ***2.82*** |
| [riken cdna d130037m23 gene](http://niaid.abcc.ncifcrf.gov/geneReportFull.jsp?rowids=116568) | 1457321_at | 1.31 | 1.11 | ***2.81*** |
| [cdna sequence ab124611](http://niaid.abcc.ncifcrf.gov/geneReportFull.jsp?rowids=18458) | 1438475_at | 1.49 | -1.46 | ***2.8*** |
| [riken cdna 5730583k22 gene](http://niaid.abcc.ncifcrf.gov/geneReportFull.jsp?rowids=149090) | 1439568_at | -1.37 | 1.61 | ***2.78*** |
| [riken cdna d430047d06 gene](http://niaid.abcc.ncifcrf.gov/geneReportFull.jsp?rowids=119891) | 1458571_at | -1.26 | -1.16 | ***2.74*** |
| [toll-like receptor 13](http://niaid.abcc.ncifcrf.gov/geneReportFull.jsp?rowids=148205) | 1457753_at | 1.13 | 1.24 | ***2.73*** |
| [dna segment, chr 17, human d6s56e 5](http://niaid.abcc.ncifcrf.gov/geneReportFull.jsp?rowids=145185) | 1417821_at | 1.23 | 1.99 | ***2.72*** |
| [,gb:ai448322 /db_xref=gi:4281710 /db_xref=mp59c02.x1 /clone=image:573506 /fea=est /cnt=2 /tid=mm.154154.1 /tier=consend /stk=2 /ug=mm.154154 /ug_title=ests](http://niaid.abcc.ncifcrf.gov/geneReportFull.jsp?rowids=3167741) | 1447175_at | -1.36 | -1.19 | ***2.71*** |
| [olfactomedin 4](http://niaid.abcc.ncifcrf.gov/geneReportFull.jsp?rowids=26723) | 1437060_at | 1.21 | 1.08 | ***2.68*** |
| [serologically defined colon cancer antigen 3](http://niaid.abcc.ncifcrf.gov/geneReportFull.jsp?rowids=10357) | 1456106_x_at | 1 | 1.48 | ***2.68*** |
| [radical s-adenosyl methionine domain containing 2](http://niaid.abcc.ncifcrf.gov/geneReportFull.jsp?rowids=28258) | 1436058_at | 2.64 | 1.97 | ***2.63*** |
| [expressed sequence ai451617](http://niaid.abcc.ncifcrf.gov/geneReportFull.jsp?rowids=26580) | 1438716_at | -1.11 | 1.49 | ***2.61*** |
| [strawberry notch homolog (drosophila)](http://niaid.abcc.ncifcrf.gov/geneReportFull.jsp?rowids=37288) | 1443721_x_at | 1.28 | -1.19 | ***2.6*** |
| [calponin 3, acidic](http://niaid.abcc.ncifcrf.gov/geneReportFull.jsp?rowids=39240) | 1438354_x_at | 1.08 | 1.48 | ***2.57*** |
| [protein tyrosine phosphatase, receptor-type, f interacting protein, binding protein 2](http://niaid.abcc.ncifcrf.gov/geneReportFull.jsp?rowids=14004) | 1444516_at | 1.79 | 1.41 | ***2.55*** |
| [riken cdna 2210011c24 gene](http://niaid.abcc.ncifcrf.gov/geneReportFull.jsp?rowids=27287) | 1429953_at | -1.04 | 1.12 | ***2.55*** |
| [musculoskeletal, embryonic nuclear protein 1](http://niaid.abcc.ncifcrf.gov/geneReportFull.jsp?rowids=19515) | 1427201_at | 1.07 | -1.07 | ***2.54*** |
| [riken cdna 9130604c24 gene](http://niaid.abcc.ncifcrf.gov/geneReportFull.jsp?rowids=79773) | 1440961_at | -1.26 | 1.08 | ***2.5*** |
| [,gb:ai853801 /db_xref=gi:5497707 /db_xref=ui-m-bh0-ajg-g-12-0-ui.s1 /clone=ui-m-bh0-ajg-g-12-0-ui /fea=est /cnt=2 /tid=mm.140741.1 /tier=consend /stk=2 /ug=mm.140741 /ug_title=ests, highly similar to s26404 alternative splicing factor asf - mouse (m.musculus)](http://niaid.abcc.ncifcrf.gov/geneReportFull.jsp?rowids=3167791) | 1447357_at | 1.08 | -1.21 | ***2.49*** |
| [ceruloplasmin](http://niaid.abcc.ncifcrf.gov/geneReportFull.jsp?rowids=145690) | 1448735_at | 1.3 | 1.72 | ***2.48*** |
| [patatin-like phospholipase domain containing 2](http://niaid.abcc.ncifcrf.gov/geneReportFull.jsp?rowids=26846) | 1428591_at | -1.69 | 2.03 | ***2.48*** |
| [thioredoxin domain containing 9](http://niaid.abcc.ncifcrf.gov/geneReportFull.jsp?rowids=12837) | 1436108_at | 1.1 | 1.07 | ***2.47*** |
| [,gb:bb043576 /db_xref=gi:16258790 /db_xref=bb043576 /clone=6030475i06 /fea=est /cnt=4 /tid=mm.130935.1 /tier=consend /stk=4 /ug=mm.130935 /ug_title=ests](http://niaid.abcc.ncifcrf.gov/geneReportFull.jsp?rowids=3168143) | 1457549_at | -1.58 | 1.87 | ***2.45*** |
| [riken cdna 2610011e03 gene](http://niaid.abcc.ncifcrf.gov/geneReportFull.jsp?rowids=85979) | 1454404_at | 1.03 | -1.18 | ***2.42*** |
| [hepatoma-derived growth factor, related protein 2](http://niaid.abcc.ncifcrf.gov/geneReportFull.jsp?rowids=16997) | 1438912_at | 1.3 | 1.37 | ***2.41*** |
| [,gb:aa266723 /db_xref=gi:1903565 /db_xref=mz98f08.r1 /clone=image:721479 /fea=est /cnt=12 /tid=mm.29940.1 /tier=consend /stk=0 /ug=mm.29940 /ug_title=ests](http://niaid.abcc.ncifcrf.gov/geneReportFull.jsp?rowids=3167885) | 1448021_at | 2.47 | 1.25 | ***2.39*** |
| [mitochondrial ribosomal protein l52](http://niaid.abcc.ncifcrf.gov/geneReportFull.jsp?rowids=23784) | 1455366_at | -1.51 | -1.12 | ***2.39*** |
| [,gb:aw551197 /db_xref=gi:7196625 /db_xref=l0075b06-3 /clone=l0075b06 /fea=est /cnt=2 /tid=mm.102949.1 /tier=consend /stk=2 /ug=mm.102949 /ug_title=ests](http://niaid.abcc.ncifcrf.gov/geneReportFull.jsp?rowids=3167615) | 1446785_at | -1.47 | 1.1 | ***2.37*** |
| [histone 1, h1t](http://niaid.abcc.ncifcrf.gov/geneReportFull.jsp?rowids=144546) | 1450608_at | -1.15 | -1.02 | ***2.37*** |
| [signal transducer and activator of transcription 1](http://niaid.abcc.ncifcrf.gov/geneReportFull.jsp?rowids=15474) | 1440481_at | 1.4 | 1.3 | ***2.37*** |
| [galectin-related inter-fiber protein](http://niaid.abcc.ncifcrf.gov/geneReportFull.jsp?rowids=25458) | 1454270_at | 1.05 | -1.28 | ***2.34*** |
| [riken cdna 0610007l01 gene](http://niaid.abcc.ncifcrf.gov/geneReportFull.jsp?rowids=12460) | 1458603_at | -1.03 | -1.18 | ***2.33*** |
| [riken cdna 4930444g20 gene](http://niaid.abcc.ncifcrf.gov/geneReportFull.jsp?rowids=30737) | 1430390_x_at | 2.09 | 1.47 | ***2.3*** |
| [complement component 1, r subcomponent](http://niaid.abcc.ncifcrf.gov/geneReportFull.jsp?rowids=8227) | 1456437_x_at | 1.14 | 1.17 | ***2.27*** |
| [riken cdna 9930033d15 gene](http://niaid.abcc.ncifcrf.gov/geneReportFull.jsp?rowids=120234) | 1444775_at | -1.67 | 1.05 | ***2.26*** |
| [pellino 1](http://niaid.abcc.ncifcrf.gov/geneReportFull.jsp?rowids=15163) | 1431060_at | -1.33 | -1.28 | ***2.25*** |
| [leucine zipper transcription factor-like 1](http://niaid.abcc.ncifcrf.gov/geneReportFull.jsp?rowids=17721) | 1431323_at | 1.21 | 1.3 | ***2.23*** |
| [phd finger protein 8](http://niaid.abcc.ncifcrf.gov/geneReportFull.jsp?rowids=45401) | 1459392_at | -1.38 | 1.13 | ***2.22*** |
| [peptidoglycan recognition protein 1](http://niaid.abcc.ncifcrf.gov/geneReportFull.jsp?rowids=16180) | 1449184_at | -1.22 | -1.03 | ***2.21*** |
| [riken cdna 2600011c06 gene](http://niaid.abcc.ncifcrf.gov/geneReportFull.jsp?rowids=17215) | 1444496_at | -1.35 | 1.24 | ***2.21*** |
| [,gb:bb153601 /db_xref=gi:16268103 /db_xref=bb153601 /clone=a130015c20 /fea=est /cnt=3 /tid=mm.207495.1 /tier=consend /stk=3 /ug=mm.207495 /ug_title=ests](http://niaid.abcc.ncifcrf.gov/geneReportFull.jsp?rowids=3168229) | 1458088_at | -1.22 | 1.5 | ***2.19*** |
| [gap junction membrane channel protein alpha 1](http://niaid.abcc.ncifcrf.gov/geneReportFull.jsp?rowids=144213) | 1438650_x_at | 1.03 | 1.04 | ***2.19*** |
| [hypothetical loc545968](http://niaid.abcc.ncifcrf.gov/geneReportFull.jsp?rowids=6519) | 1443835_x_at | -1.01 | -1.1 | ***2.19*** |
| [riken cdna 1110014k08 gene](http://niaid.abcc.ncifcrf.gov/geneReportFull.jsp?rowids=15923) | 1443835_x_at | -1.01 | -1.1 | ***2.19*** |
| [transgelin](http://niaid.abcc.ncifcrf.gov/geneReportFull.jsp?rowids=25740) | 1423505_at | 1 | 1.46 | ***2.18*** |
| [microfibrillar associated protein 5](http://niaid.abcc.ncifcrf.gov/geneReportFull.jsp?rowids=145046) | 1418454_at | 1.14 | 1.04 | ***2.17*** |
| [riken cdna 0610010o12 gene](http://niaid.abcc.ncifcrf.gov/geneReportFull.jsp?rowids=30602) | 1441883_at | -1.21 | -1.3 | ***2.14*** |
| [myod family inhibitor domain containing](http://niaid.abcc.ncifcrf.gov/geneReportFull.jsp?rowids=149260) | 1427040_at | 1.41 | 1.54 | ***2.08*** |
| [riken cdna 4631426b19 gene](http://niaid.abcc.ncifcrf.gov/geneReportFull.jsp?rowids=22091) | 1429459_at | 1.14 | 1.07 | ***2.06*** |
| [ankyrin repeat and socs box-containing protein 5](http://niaid.abcc.ncifcrf.gov/geneReportFull.jsp?rowids=22418) | 1449356_at | -1.1 | 1.18 | ***2.04*** |
| [au rna binding protein/enoyl-coenzyme a hydratase](http://niaid.abcc.ncifcrf.gov/geneReportFull.jsp?rowids=9232) | 1458436_at | -1.03 | -1.18 | ***2.03*** |
| [riken cdna 4933430f16 gene](http://niaid.abcc.ncifcrf.gov/geneReportFull.jsp?rowids=38204) | 1429325_at | 1.64 | 1.7 | ***2.03*** |
| [hypothetical protein 9530064j02](http://niaid.abcc.ncifcrf.gov/geneReportFull.jsp?rowids=90741) | 1438726_at | -1.23 | -1.18 | ***2.02*** |
| [synaptotagmin vii](http://niaid.abcc.ncifcrf.gov/geneReportFull.jsp?rowids=90679) | 1423012_at | 1.15 | 1.04 | ***-2*** |
| [,gb:bg066901 /db_xref=gi:12549470 /db_xref=h3048d01-3 /clone=h3048d01 /fea=est /cnt=22 /tid=mm.27910.1 /tier=stack /stk=19 /ug=mm.27910 /ll=81798 /ug_gene=urml-pending /ug_title=up-regulated in myc liver](http://niaid.abcc.ncifcrf.gov/geneReportFull.jsp?rowids=3167982) | 1455240_x_at | -1.06 | 1.09 | ***-2.01*** |
| [up-regulated in myc liver](http://niaid.abcc.ncifcrf.gov/geneReportFull.jsp?rowids=54657) | 1455240_x_at | -1.06 | 1.09 | ***-2.01*** |
| [,gb:aa420337 /db_xref=gi:2080894 /db_xref=vc51d03.r1 /clone=image:778085 /fea=est /cnt=1 /tid=mm.6761.1 /tier=consend /stk=0 /ug=mm.6761 /ug_title=est](http://niaid.abcc.ncifcrf.gov/geneReportFull.jsp?rowids=3167939) | 1449806_at | 1.07 | -1.58 | ***-2.02*** |
| [riken cdna 4930596i21 gene](http://niaid.abcc.ncifcrf.gov/geneReportFull.jsp?rowids=77918) | 1446330_at | -1.26 | 1.13 | ***-2.02*** |
| [adenosine deaminase, rna-specific, b1](http://niaid.abcc.ncifcrf.gov/geneReportFull.jsp?rowids=27962) | 1421480_a_at | -1.13 | -1.39 | ***-2.03*** |
| [wd repeat domain 47](http://niaid.abcc.ncifcrf.gov/geneReportFull.jsp?rowids=10513) | 1437257_at | 1.14 | -1.24 | ***-2.04*** |
| [netrin g1](http://niaid.abcc.ncifcrf.gov/geneReportFull.jsp?rowids=26260) | 1426338_a_at | 1.07 | -1.41 | ***-2.05*** |
| [riken cdna 1700026j12 gene](http://niaid.abcc.ncifcrf.gov/geneReportFull.jsp?rowids=13257) | 1453793_at | -1.2 | -1.64 | ***-2.05*** |
| [,gb:bb395376 /db_xref=gi:16409304 /db_xref=bb395376 /clone=c230091n23 /fea=est /cnt=2 /tid=mm.126187.1 /tier=consend /stk=2 /ug=mm.126187 /ug_title=ests](http://niaid.abcc.ncifcrf.gov/geneReportFull.jsp?rowids=3168501) | 1459359_at | -1.39 | -1.43 | ***-2.06*** |
| [riken cdna 2410193c02 gene](http://niaid.abcc.ncifcrf.gov/geneReportFull.jsp?rowids=145090) | 1429941_at | -1.01 | 1.17 | ***-2.09*** |
| [,gb:bi076724 /db_xref=gi:14515381 /db_xref=l0228h03-3 /clone=l0228h03 /fea=est /cnt=2 /tid=mm.197270.1 /tier=consend /stk=2 /ug=mm.197270 /ug_title=ests, moderately similar to s12207 hypothetical protein (m.musculus)](http://niaid.abcc.ncifcrf.gov/geneReportFull.jsp?rowids=3167663) | 1446927_at | -1.13 | 1.08 | ***-2.12*** |
| [cdna sequence bc023818](http://niaid.abcc.ncifcrf.gov/geneReportFull.jsp?rowids=14417) | 1439855_at | 1.53 | -1.64 | ***-2.14*** |
| [a kinase (prka) anchor protein 6](http://niaid.abcc.ncifcrf.gov/geneReportFull.jsp?rowids=6828) | 1444357_at | -1.16 | -1.71 | ***-2.15*** |
| [riken cdna 1700001l19 gene](http://niaid.abcc.ncifcrf.gov/geneReportFull.jsp?rowids=7269) | 1453643_at | -1.3 | -1.01 | ***-2.15*** |
| [tripartite motif-containing 36](http://niaid.abcc.ncifcrf.gov/geneReportFull.jsp?rowids=16578) | 1455837_at | 1.42 | -1.52 | ***-2.2*** |
| [,gb:aw492805 /db_xref=gi:7063086 /db_xref=ui-m-bh3-atm-a-09-0-ui.s1 /clone=ui-m-bh3-atm-a-09-0-ui /fea=est /cnt=7 /tid=mm.102127.1 /tier=consend /stk=3 /ug=mm.102127 /ug_title=ests](http://niaid.abcc.ncifcrf.gov/geneReportFull.jsp?rowids=3168162) | 1457729_at | -1.71 | -1.07 | ***-2.23*** |
| [expressed sequence bb220380](http://niaid.abcc.ncifcrf.gov/geneReportFull.jsp?rowids=32759) | 1437956_at | 1.81 | 1.06 | ***-2.23*** |
| [g-protein coupled receptor 12](http://niaid.abcc.ncifcrf.gov/geneReportFull.jsp?rowids=14215) | 1457729_at | -1.71 | -1.07 | ***-2.23*** |
| [hippocalcin](http://niaid.abcc.ncifcrf.gov/geneReportFull.jsp?rowids=14890) | 1425833_a_at | -1.14 | -1.49 | ***-2.27*** |
| [,gb:bm207422 /db_xref=gi:17763245 /db_xref=c0612e08-3 /clone=c0612e08 /fea=est /cnt=3 /tid=mm.2354.1 /tier=consend /stk=2 /ug=mm.2354 /ug_title=ests](http://niaid.abcc.ncifcrf.gov/geneReportFull.jsp?rowids=3167328) | 1445617_at | -1.65 | -1.12 | ***-2.33*** |
| [src homology 2 domain-containing transforming protein c2](http://niaid.abcc.ncifcrf.gov/geneReportFull.jsp?rowids=38117) | 1437944_at | 1.16 | -1.25 | ***-2.34*** |
| [t-cell receptor beta, variable 13](http://niaid.abcc.ncifcrf.gov/geneReportFull.jsp?rowids=7126) | 1425854_x_at | -1.14 | -1.63 | ***-2.34*** |
| [riken cdna 2310009b15 gene](http://niaid.abcc.ncifcrf.gov/geneReportFull.jsp?rowids=16871) | 1455761_at | -1.16 | -1.23 | ***-2.35*** |
| [similar to novel protein](http://niaid.abcc.ncifcrf.gov/geneReportFull.jsp?rowids=135736) | 1455761_at | -1.16 | -1.23 | ***-2.35*** |
| [aldehyde dehydrogenase 2, mitochondrial](http://niaid.abcc.ncifcrf.gov/geneReportFull.jsp?rowids=8123) | 1434988_x_at | 1.14 | -1.62 | ***-2.37*** |
| [methyltransferase-like 1](http://niaid.abcc.ncifcrf.gov/geneReportFull.jsp?rowids=29241) | 1447683_x_at | -1.33 | -1.57 | ***-2.42*** |
| [riken cdna 2410150o07 gene](http://niaid.abcc.ncifcrf.gov/geneReportFull.jsp?rowids=146182) | 1438339_at | 1.05 | 1.22 | ***-2.46*** |
| [similar to novel protein](http://niaid.abcc.ncifcrf.gov/geneReportFull.jsp?rowids=72056) | 1438158_at | -1.18 | -1.6 | ***-2.46*** |
| [similar to tubulin tyrosine ligase-like family, member 7](http://niaid.abcc.ncifcrf.gov/geneReportFull.jsp?rowids=37588) | 1430615_at | -1.2 | -1.46 | ***-2.46*** |
| [syntrophin, gamma 1](http://niaid.abcc.ncifcrf.gov/geneReportFull.jsp?rowids=15622) | 1432287_a_at | -1.17 | -1.89 | ***-2.47*** |
| [hypothetical gene supported by ak039686](http://niaid.abcc.ncifcrf.gov/geneReportFull.jsp?rowids=3988) | 1440290_at | -1.14 | -1.46 | ***-2.48*** |
| [riken cdna 2310035c23 gene](http://niaid.abcc.ncifcrf.gov/geneReportFull.jsp?rowids=144516) | 1430126_at | 1.5 | -1.36 | ***-2.5*** |
| [ubiquitin specific peptidase 31](http://niaid.abcc.ncifcrf.gov/geneReportFull.jsp?rowids=4076) | 1419828_at | 1.01 | -1.34 | ***-2.53*** |
| [calcium binding protein 7](http://niaid.abcc.ncifcrf.gov/geneReportFull.jsp?rowids=8200) | 1425963_at | 1.24 | -1.49 | ***-2.55*** |
| [riken cdna 2810416g20 gene](http://niaid.abcc.ncifcrf.gov/geneReportFull.jsp?rowids=19428) | 1431220_at | 1.39 | 1.5 | ***-2.59*** |
| [riken cdna 4933407c09 gene](http://niaid.abcc.ncifcrf.gov/geneReportFull.jsp?rowids=86685) | 1454348_at | -1.22 | -1.25 | ***-2.59*** |
| [ubiquitin specific peptidase 43](http://niaid.abcc.ncifcrf.gov/geneReportFull.jsp?rowids=147071) | 1424866_at | 1.72 | 1 | ***-2.63*** |
| [riken cdna 1700008b15 gene](http://niaid.abcc.ncifcrf.gov/geneReportFull.jsp?rowids=17543) | 1445238_at | -1.13 | -1.13 | ***-2.85*** |
| [riken cdna 2810457d07 gene](http://niaid.abcc.ncifcrf.gov/geneReportFull.jsp?rowids=15211) | 1430649_at | -1.93 | 1.16 | ***-2.85*** |
| [expressed sequence ai841796](http://niaid.abcc.ncifcrf.gov/geneReportFull.jsp?rowids=10120) | 1457699_at | -1.28 | -1.79 | ***-2.9*** |
| [riken cdna 4933406l05 gene](http://niaid.abcc.ncifcrf.gov/geneReportFull.jsp?rowids=121788) | 1453856_at | 1.29 | -1.22 | ***-2.92*** |
| [rho/rac guanine nucleotide exchange factor (gef) 18](http://niaid.abcc.ncifcrf.gov/geneReportFull.jsp?rowids=36039) | 1457219_at | 1.21 | -1.69 | ***-3.12*** |
| [signaling molecule attp](http://niaid.abcc.ncifcrf.gov/geneReportFull.jsp?rowids=4576) | 1421785_at | 1.32 | 1.09 | ***-3.12*** |
| [low density lipoprotein receptor](http://niaid.abcc.ncifcrf.gov/geneReportFull.jsp?rowids=8818) | 1450383_at | 1.28 | 1.43 | ***-3.27*** |
| [scratch homolog 1, zinc finger protein (drosophila)](http://niaid.abcc.ncifcrf.gov/geneReportFull.jsp?rowids=13810) | 1421368_at | 1.07 | -1.61 | ***-4.05*** |
