# Supplementary material for: Gene expression profiling in a mouse model of infantile neuronal ceroid lipofuscinosis reveals upregulation of immediate early genes and mediators of the inflammatory response
Source: BMC Neurosci. 2007 Nov 16;8:95. doi: 10.1186/1471-2202-8-95 (PMC2204004; doi:10.1186/1471-2202-8-95)
Supplement: Additional File 7 — Genes encoding lysosomal proteins upregulated in PPT1 knockout brain (Microsoft Word table). [file 1471-2202-8-95-S7.doc]

| Additional file 7. Genes encoding lysosomal proteins upregulated in PPT1 knockout brain. | | | |
| --- | --- | --- | --- |
| Gene ID | Gene Symbol | Gene Name | Fold-change |
|  |  |  |  |
| [12514](http://bioinfo.vanderbilt.edu/webgestalt/llid_info.php?llid=12514) | Cd68 | CD68 antigen | 3.9 |
| [13040](http://bioinfo.vanderbilt.edu/webgestalt/llid_info.php?llid=13040) | Ctss | cathepsin S | 2.4 |
| [14960](http://bioinfo.vanderbilt.edu/webgestalt/llid_info.php?llid=14960) | H2-Aa | histocompatibility 2, class II antigen A, alpha | 2.4 |
| [13036](http://bioinfo.vanderbilt.edu/webgestalt/llid_info.php?llid=13036) | Ctsh | cathepsin H | 2.2 |
| [65972](http://bioinfo.vanderbilt.edu/webgestalt/llid_info.php?llid=65972) | Ifi30 | interferon gamma inducible protein 30 | 2.2 |
| [64138](http://bioinfo.vanderbilt.edu/webgestalt/llid_info.php?llid=64138) | Ctsz | cathepsin Z | 2.1 |
| [13033](http://bioinfo.vanderbilt.edu/webgestalt/llid_info.php?llid=13033) | Ctsd | cathepsin D | 2.1 |
| [13032](http://bioinfo.vanderbilt.edu/webgestalt/llid_info.php?llid=13032) | Ctsc | cathepsin C | 1.8 |
| [16784](http://bioinfo.vanderbilt.edu/webgestalt/llid_info.php?llid=16784) | Lamp2 | lysosomal membrane glycoprotein 2 | 1.7 |
| [110006](http://bioinfo.vanderbilt.edu/webgestalt/llid_info.php?llid=110006) | Gusb | glucuronidase, beta | 1.7 |
| [11758](http://bioinfo.vanderbilt.edu/webgestalt/llid_info.php?llid=11758) | Prdx6 | peroxiredoxin 6 | 1.7 |
| [15212](http://bioinfo.vanderbilt.edu/webgestalt/llid_info.php?llid=15212) | Hexb | hexosaminidase B | 1.5 |
| [226421](http://bioinfo.vanderbilt.edu/webgestalt/llid_info.php?llid=226421) | 5430435G22Rik | RIKEN cDNA 5430435G22 gene | 1.5 |
| [15211](http://bioinfo.vanderbilt.edu/webgestalt/llid_info.php?llid=15211) | Hexa | hexosaminidase A | 1.5 |
| [16149](http://bioinfo.vanderbilt.edu/webgestalt/llid_info.php?llid=16149) | Cd74 | CD74 antigen | 1.5 |
| [13039](http://bioinfo.vanderbilt.edu/webgestalt/llid_info.php?llid=13039) | Ctsl | cathepsin L | 1.5 |
| [19025](http://bioinfo.vanderbilt.edu/webgestalt/llid_info.php?llid=19025) | Ppgb | protective protein for beta-galactosidase | 1.4 |
| [17159](http://bioinfo.vanderbilt.edu/webgestalt/llid_info.php?llid=17159) | Man2b1 | mannosidase 2, alpha B1 | 1.4 |
| [13030](http://bioinfo.vanderbilt.edu/webgestalt/llid_info.php?llid=13030) | Ctsb | cathepsin B | 1.4 |
| [11593](http://bioinfo.vanderbilt.edu/webgestalt/llid_info.php?llid=11593) | Aga | aspartylglucosaminidase | 1.4 |
| [83768](http://bioinfo.vanderbilt.edu/webgestalt/llid_info.php?llid=83768) | Dpp7 | dipeptidylpeptidase 7 | 1.4 |
| [19141](http://bioinfo.vanderbilt.edu/webgestalt/llid_info.php?llid=19141) | Lgmn | legumain | 1.4 |
| [16783](http://bioinfo.vanderbilt.edu/webgestalt/llid_info.php?llid=16783) | Lamp1 | lysosomal membrane glycoprotein 1 | 1.3 |
| [11605](http://bioinfo.vanderbilt.edu/webgestalt/llid_info.php?llid=11605) | Gla | galactosidase, alpha | 1.3 |
| [12751](http://bioinfo.vanderbilt.edu/webgestalt/llid_info.php?llid=12751) | Tpp1 | tripeptidyl peptidase I | 1.3 |
| [14667](http://bioinfo.vanderbilt.edu/webgestalt/llid_info.php?llid=14667) | Gm2a | GM2 ganglioside activator protein | 1.3 |
| [11886](http://bioinfo.vanderbilt.edu/webgestalt/llid_info.php?llid=11886) | Asah1 | N-acylsphingosine amidohydrolase 1 | 1.2 |
| [19063](http://bioinfo.vanderbilt.edu/webgestalt/llid_info.php?llid=19063) | Ppt1 | palmitoyl-protein thioesterase 1 | .04 |
