# Supplementary material for: Gene expression profiling in a mouse model of infantile neuronal ceroid lipofuscinosis reveals upregulation of immediate early genes and mediators of the inflammatory response
Source: BMC Neurosci. 2007 Nov 16;8:95. doi: 10.1186/1471-2202-8-95 (PMC2204004; doi:10.1186/1471-2202-8-95)
Supplement: Additional File 9 — Primer sequences used for validation of microarray data (A Microsoft Word Table). [file 1471-2202-8-95-S9.doc]

Additional File 9

Primer sequences for microarray validation by quantitative PCR

Primers Sequence Concentration

Actin forward 5' CGG TTC CGA TGC CCT G 3′ 100 nM

Actin reverse 5' TGG ATG CCA CAG GAT TCC AT 3′ 100 nM

Ctsd forward 5´CCG GCG TCT TGC TGC TCA 3′ 100 nM

Ctsd reverse 5´TTG CGC AGA GGG ATT CTG AT 3′ 100 nM

C1qa forward 5´AAA GGC AAT CCA GGC AAT ATC A 3′ 200 nM

C1qa reverse 5´TGG TTC TGG TAT GGA CTC TCC 3′ 200 nM

C4 forward 5´TGC CAA TGA AGA CTA CGA AGA C 3′ 200 nM

C4 reverse 5´TGC CAT TTC GCC AGA TAC ACA 3′ 200 nM

Serpina3n forward 5´TCC ATC TCC ACC GAC TAC AG 3′ 200 nM

Serpina3n reverse 5´CAG CCA CAT CCA GCA CAG 3′ 200 nM

Fos forward 5´CCC ATC CTT ACG GAC TCC C 3′ 200 nM

Fos reverse 5´GAG ATA GCT GCT CTA CTT TGC C 3′ 200 nM

Lzp-s forward 5´CCC CAA GGC ATT CGA GC 3′ 200 nM

Lzp-s reverse 5´TGG GAC AGA TCT CGG TTT TGA 3′ 200 nM

A2m forward 5´CGA GCA CCC CTT CTC TGT G 3′ 200 nM

A2m reverse 5´CTT CCC ATA GGT GTA TAT GCC AC 3′ 200 nM

Gp49a forward 5' AGT GTC GTC ACA AAA ATA AGG CT 3′ 300 nM

Gp49a reverse 5' CCT GGG CGT ACA CAA TTC CC 3′ 300 nM

Lgals3 forward 5´ATG ACC TGC CCT TGC CTG 3′ 200 nM

Lgals3 reverse 5´TCA CTG TGC CCA TGA TTG TGA 3′ 200 nM

Cap1 forward 5´TGA AGA TGA GTA AGG AGA TCG GG 3′ 200 nM

Cap1 reverse 5´AGG TGC CAA CAA ATC AGA AAG TT 3′ 200 nM

Gfap forward 5´CCA AGC CAA ACA CGA AGC TAA 3′ 200 nM

Gfap reverse 5´CAT TTG CCG CTC TAG GGA CTC 3′ 200 nM

Mid1 forward 5´ATA TCT TCA CGG TGA AGG CCA 3′ 300 nM

Mid1 reverse 5´TTC CGG GCT CGC TGC 3′ 300 nM

Ndufs-5 forward 5´GCC TGG ACC GGC ACT TT 3′ 300 nM

Ndufs-5 reverse 5´TGG CAC CGA GCG GC 3′ 300 nM

Erdr1 forward 5´GGT CAA GAT GTA TGT GCC ACC 3′ 200 nM

Erdr1 reverse 5´GCT TCT ACG TGT GTG CTT TCG 3′ 200 nM
